# Statistical quantification of confounding bias in machine learning models

**DOI:** 10.1093/gigascience/giac082

**Published:** 2022-08-26

**Authors:** Tamas Spisak

**Affiliations:** Center for Translational Neuro- and Behavioral Sciences, Institute for Diagnostic and Interventional Radiology and Neuroradiology, Center University Hospital Essen, Essen, D-45147, Germany

**Keywords:** machine learning, predictive modeling, confounding bias, confounder test, conditional independence, conditional permutation

## Abstract

**Background:**

The lack of nonparametric statistical tests for confounding bias significantly hampers the development of robust, valid, and generalizable predictive models in many fields of research. Here I propose the *partial confounder test*, which, for a given confounder variable, probes the null hypotheses of the model being *unconfounded*.

**Results:**

The test provides a strict control for type I errors and high statistical power, even for nonnormally and nonlinearly dependent predictions, often seen in machine learning. Applying the proposed test on models trained on large-scale functional brain connectivity data (*N*= 1,865) (i) reveals previously unreported confounders and (ii) shows that state-of-the-art confound mitigation approaches may fail preventing confounder bias in several cases.

**Conclusions:**

The proposed test (implemented in the package *mlconfound*; https://mlconfound.readthedocs.io) can aid the assessment and improvement of the generalizability and validity of predictive models and, thereby, fosters the development of clinically useful machine learning biomarkers.

Key points

- The lack of statistical tests for confounding bias hampers the development of machine learning–based biomarker candidates.
- The partial confounder test provides a model-agnostic approach for quantifying confounding bias.
- It provides strict control for type I errors and high statistical power with minimal assumptions.
- Deploying the test on functional brain connectivity data reveals that confounding bias can be problematic even if confound mitigation approaches are used.
- The test provides objective criteria to assess the specificity, generalizability, and biomedical validity of biomarker candidates.

## Background

Predictive modelling uses multivariate statistical learning to aggregate information from a set of features with the aim of predicting an unknown outcome. This approach has recently become increasingly important in biomedical research and holds promise for delivering biomarkers that substantially impact clinical practice and public health [1–4]. When evaluating the usefulness and applicability of such markers, predictive performance is far from being the only important consideration. Biomedical validity and generalizability across contexts and populations are also fundamental requirements for candidate biomarkers [5–7].

Spurious, out-of-interest associations between the predictor variables (features) and the prediction target can be detrimental to the model’s biomedical validity and generalizability. This phenomenon is often called confounding bias [8]. Confounding bias can be driven by various sources. For instance, measurement artifacts (e.g., motion artifacts in magnetic resonance imaging–based predictive models) are well known as a potential confounder that can bias the predictive model’s output in, among others, Alzheimer’s disease [9], attention-deficit/hyperactivity disorder [10, 11], or autism spectrum disorder (ASD) [12–14]). Confounding bias is, however, not restricted to measurement artifacts. Depending on the research question, several demographic and psychometric variables or the time of day of the data acquisition [15] can emerge as confounders. As a characteristic example, models trained to predict intelligence [16,17] might provide a statistically significant predictive performance by picking up solely on age-related variance [18,19]. Moreover, various types of systematic sampling bias, as well as stochastic group differences in the training sample, can result in confounded models (e.g., racially biased machine learning models [6, 20, 21]).

Confounding bias is especially problematic in population neuroscience studies. While large-scale multisite studies are of key importance for developing robust machine learning markers [22], most of the confounding effects are much more likely to occur in such big, longer-term studies [23], and batch and center effects may arise as additional sources of confounding bias [24, 25].

While various data-cleaning methods and dedicated prediction algorithms may help in mitigating confounding bias [9,13, 26–29], effects of confounders can potentially bleed through into predictions even if they are being attempted to control for in the prediction algorithm (see Supplementary Analysis 1 for an example), and it is often unclear which variables should be considered as confounders. In a number of cases, removing or controlling for a confounder can remove variance of interest and complicate model interpretations [24,29, 30], rendering the choice of confound mitigation strategy as one of the most difficult compromises in predictive model development.

Powerful and robust statistical tests for quantifying confounding bias in predictive models could substantially foster both the identification of confounders to correct for and the assessment of the effectiveness of various confound mitigation approaches. It is tempting to think about confounding bias as the *conditional dependence* of the model output on the observed confounder, given the target variable. However, the proper evaluation of conditional independence among these variables is challenging. Namely, even in the presence of a slight nonnormality and/or nonlinearity of the involved conditional distributions, the “conditional” analogs of the most popular bivariate nonparametric tests (like the partial Spearman correlation; see Fig. 3) are not valid measures of conditional independence. Although warnings about this issue were given from early on [31] and received a fair amount of attention recently [32–36], the magnitude of the problem may not be fully appreciated in case of predictive model diagnostics, where nonnormality and nonlinearity of the model output can be frequently seen (see Supplementary Figs. S10–S11), as a consequence of, for example, feature-set characteristics and model regularization [37,38].

Recently, 2 different approaches were proposed for quantifying confounding bias [39,40]. However, these methods either fail to control type I error (as known in the case of balanced permutations [41,42], used in Neto et al. [39]) or do not provide *P* values at all [40]. Moreover, without some modifications, they are only applicable for categorical variables and involve refitting the model, which may not be feasible for models with high computational cost (e.g., when trained with nested cross-validation).

This work aims to construct a statistical test for confounding bias that (i) guarantees valid type I error control for arbitrary models, even if nonnormal and/or nonlinear dependencies are involved; (ii) does not require refitting the model; and (iii) is applicable for classification as well as for prediction problems and both with numerical and categorical confounders.

## Methods

### Notation and background

In a predictive modeling setting, let $\boldsymbol{y}$ denote the target variable, $\mathbf {X}$ denote the feature variables, $\boldsymbol{\hat{y}}$ denote model output (i.e., the predictions for $\boldsymbol{y}$), and $\boldsymbol{c}$ denote a variable that is considered a confounder. Note that $\boldsymbol{y}$ and $\boldsymbol{c}$ must be observed during the experiment, whereas $\boldsymbol{\hat{y}}$ is provided by the predictive model. Confounding bias typically emerges in situations where $\mathbf {X} \leftarrow \boldsymbol{c}\rightarrow \boldsymbol{y}$ (arrows denoting dependence of $\mathbf {X}$ and $\boldsymbol{y}$ on $\boldsymbol{c}$), although $\boldsymbol{c}\rightarrow \boldsymbol{y}$ is not a prerequisite. After fitting the predictive model, we aim to construct predictions based on features unseen during the model training procedure: $\mathbf {X} \rightarrow \boldsymbol{\hat{y}}$ so that $\boldsymbol{y}\rightarrow \boldsymbol{\hat{y}}$. Obviously, a strong association between $\boldsymbol{\hat{y}}$ and $\boldsymbol{c}$ may indicate that the model is biased; its predictions are driven by the confounder rather than information about the target variable. Assessing the simple bivariate (unconditioned) dependence ($H0: \boldsymbol{\hat{y}}{\perp\!\!\!\perp} \boldsymbol{c}$) between $\boldsymbol{\hat{y}}$ and $\boldsymbol{c}$ (or any of the $\boldsymbol{y}$, $\boldsymbol{\hat{y}}$, $\boldsymbol{c}$ variables) is, however, insufficient for the proper characterization of confounding bias in predictive modeling. For instance, even if $\boldsymbol{\hat{y}}{\perp\!\!\!\perp} \boldsymbol{c}$ is false, $\boldsymbol{\hat{y}}$ might be only marginally dependent on $\boldsymbol{c}$, due to the dependence of both on $\boldsymbol{y}$. In other words, if the target variable $\boldsymbol{y}$ displays a true association to the confounder variable $\boldsymbol{c}$, a model that is completely blind to $\boldsymbol{c}$ (i.e., not confounded at all) might still provide outputs $\boldsymbol{\hat{y}}$ that are significantly associated with $\boldsymbol{c}$.

#### Conditional independence for testing confounding bias

Instead of focusing on the “unconditioned” independence between the confounder and the predictions, we shall consider the *conditional independence* between $\boldsymbol{\hat{y}}$ and $\boldsymbol{c}$ given $\boldsymbol{y}$ (written as $\boldsymbol{\hat{y}}{\perp\!\!\!\perp} \boldsymbol{c}| \boldsymbol{y}$), which, by definition [43], means that $\mathbb {P}(\boldsymbol{\hat{y}}, \boldsymbol{c}|\boldsymbol{y}) = \mathbb {P}(\boldsymbol{\hat{y}}|\boldsymbol{y})\mathbb {P}(\boldsymbol{c}|\boldsymbol{y})$. Testing whether $\boldsymbol{c}$ is independent from $\boldsymbol{\hat{y}}$, conditional on $\boldsymbol{y}$, is essentially checking whether the path $\boldsymbol{c}\rightarrow \mathbf {X} \rightarrow \boldsymbol{\hat{y}}$ has been blocked in the prediction algorithm. The statistical test with the null hypothesis $H0: \boldsymbol{\hat{y}}{\perp\!\!\!\perp} \boldsymbol{c}| \boldsymbol{y}$ will be referred to as the *partial confounder test*. Of note, although typically less useful in a predictive modeling context, one might also be interested in testing $\boldsymbol{\hat{y}}{\perp\!\!\!\perp} \boldsymbol{y}| \boldsymbol{c}$. We refer to the corresponding test as the *full confounder test*.

Conditional independence—in its general form—is a fundamental concept in statistics with numerous biomedical applications [33,34, 44, 45]. Recently, Shah and Peters [35] have raised important concerns regarding conditional independence testing. Their “no free lunch” theorem implies that, without placing some assumptions on the joint distribution of $(\boldsymbol{y}, \boldsymbol{\hat{y}}, \boldsymbol{c})$, conditional independence testing is effectively impossible. In other words, neither the full nor the partial confounder tests can be constructed so that—for all distributions—they provide a valid type I error control and, at the same time, a nontrivial statistical power.

This result stands in strong contrast to *unconditional* independence testing—where permutation tests [46, 47] provide a general, distribution-free solution—and it has important implications for confounder testing in predictive modeling where the distribution of the model outputs (conditioned on the target variable)—depending on the applied machine learning model—is unknown and often nonnormal and nonlinear. One of the trivial candidates for the task, partial correlation, for instance assumes that all involved variables are multivariate Gaussian and—as to be shown below in a simulated example—even its Spearman-based variant is unable to tolerate relatively small deviations from normality and linearity.

Recently, Candès et al. [33] and, based on their work, Berrett et al. [36] have demonstrated that valid and powerful conditional independence tests can be constructed with inputting distributional information about only 2 (out of the 3) variables. Specifically, the conditional permutation test (CPT) of Berrett and colleagues [36] samples from a nonuniform distribution over the set of possible permutations π of one of the variables, based on its conditional distribution of the other variable. Thereby, it incorporates the information available about the conditional distribution of interest into the permutation-based inference in a statistically valid manner.

**Figure 1:**
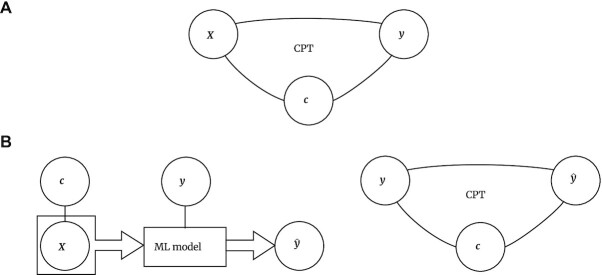
: Conditional permutation testing as a tool for predictive model diagnostics. (A) Conditional permutation testing (CPT) was originally proposed to be used on the feature variable *X*, target variable *Y*, and confounders *Z*, to perform statistical inference. (B) The proposed use of CPT in predictive modeling requires the model to be fitted first, to obtain the model’s prediction $\boldsymbol{\hat{y}}$ on $\boldsymbol{y}$. CPT is then utilized on the triplet $(\boldsymbol{y}, \boldsymbol{\hat{y}}, \boldsymbol{c})$, to test hypotheses $\boldsymbol{\hat{y}}{\perp\!\!\!\perp} \boldsymbol{c}| \boldsymbol{y}$ or $\boldsymbol{y}{\perp\!\!\!\perp} \boldsymbol{\hat{y}}| \boldsymbol{c}$. Using CPT this way allows lifting assumptions on the prediction target. However, as shown in Fig. 3, the original can still provide inflated *P* values in case of nonlinearity in the conditional distributions. False positives can be successfully eliminated by the proposed nonlinear techniques for conditional distribution modeling (Fig. 2).

Like many related papers, the work of Berrett et al. [36] was formalized as a (semi)supervised learning approach, where $\boldsymbol{X}$ is a set of predictors (features), $\boldsymbol{y}$ is the target variable, and $\boldsymbol{c}$ is a potential confounder (Fig. 1A). In this setting, testing the null hypothesis $\boldsymbol{X} {\perp\!\!\!\perp} \boldsymbol{y}| \boldsymbol{c}$ aims to determine whether the features $\boldsymbol{X}$ still affect $\boldsymbol{y}$, when controlling for $\boldsymbol{c}$. For instance, in genome-wide association studies, CPT can be used to determine whether a particular genetic variant $\boldsymbol{X}$ affects a response $\boldsymbol{y}$ such as disease status or some other phenotype, even after controlling for the rest of the genome, encoded in $\boldsymbol{c}$.

In this article, a different setting is considered, where the supervised learning model is already fitted (Fig. 1B) and we are focusing on model diagnostics by testing the triplet $(\boldsymbol{y},\boldsymbol{\hat{y}}, \boldsymbol{c})$, with the requirement of minimal assumptions on the conditional distribution of $\boldsymbol{\hat{y}}$ on $\boldsymbol{y}$ and $\boldsymbol{c}$ (Fig. 1C).

Within this setting, conditional independence testing and, specifically, the framework of conditional permutation testing allows investigating 3 different null hypotheses corresponding to the $(\boldsymbol{y}, \boldsymbol{\hat{y}}, \boldsymbol{c})$ triplet. As listed in Table 1, testing the null hypothesis $\boldsymbol{y}{\perp\!\!\!\perp} \boldsymbol{\hat{y}}| \boldsymbol{c}$ (option 1, full confounder testing) investigates whether the predictions are likely explainable solely with the confounder (i.e., whether the model is exclusively confounder driven). Testing $\boldsymbol{y}{\perp\!\!\!\perp} \boldsymbol{c}| \boldsymbol{\hat{y}}$ (option 2) addresses whether the model captures all the variance in *c* when predicting *y*. Testing the null hypothesis $\boldsymbol{\hat{y}}{\perp\!\!\!\perp} \boldsymbol{c}| \boldsymbol{y}$ (option 3, partial confounder testing) examines whether the dependence of the model output on the confounder can likely be explained by the confounder’s dependence on the target variable (i.e., whether there is any confounding bias in the model).

**Table 1.** Possibilities when testing conditional independence in potentially biased predictive models. The table lists the 3 possible null hypotheses (H0) and the variables where assumption about the joint/conditional distributions is required/not required ($\boldsymbol{y}$: prediction target, $\boldsymbol{\hat{y}}$: predictions, $\boldsymbol{c}$: confounder variable).

		H0	Assumption needed for	No assumptions about the distribution of
**1**.	$\boldsymbol{\hat{y}}{\perp\!\!\!\perp} \boldsymbol{y}| \boldsymbol{c}$	Full confounder test: model exclusively driven by the confounder	$Q(\boldsymbol{y}|\boldsymbol{c})$	$(\boldsymbol{\hat{y}},\boldsymbol{y}), (\boldsymbol{\hat{y}}, \boldsymbol{c})$
**2**.	$\boldsymbol{y}{\perp\!\!\!\perp} \boldsymbol{c}| \boldsymbol{\hat{y}}$	Model captures all variance in the confounder (not of interest)	$Q(\boldsymbol{c}|\boldsymbol{\hat{y}})$	$(\boldsymbol{y}, \boldsymbol{c}), (\boldsymbol{y}, \boldsymbol{\hat{y}})$
**3**.	$\boldsymbol{\hat{y}}{\perp\!\!\!\perp} \boldsymbol{c}| \boldsymbol{y}$	Partial confounder test: model not directly driven by the confounder	$Q(\boldsymbol{c}|\boldsymbol{y})$	$(\boldsymbol{\hat{y}}, \boldsymbol{c}), (\boldsymbol{\hat{y}}, \boldsymbol{y})$

Option 3 (i.e., partial confounder testing) is typically of interest when testing confounding bias of predictive models. Option 1 (i.e., full confounder testing) may be less useful in practice, although it might provide valuable insights in the exploratory phase of model construction. Option 2 does not seem appealing for model diagnostics, and importantly, in this case, the proposed variety of the CPT framework does not allow constructing a test that is nonparametric on $\boldsymbol{\hat{y}}$. We will therefore focus on option 3 (i.e., the partial confounder test).

**Figure 2:**
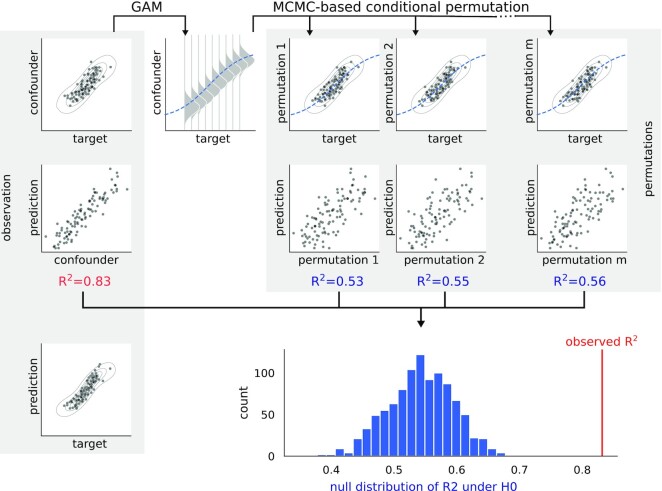
: Graphical representation of the proposed partial confounder test. The partial confounder test models the conditional distribution of the confounder, given the target variable, with a generalized additive model (GAM). The parallel-pairwise Markov chain Monte Carlo (MCMC) sampler draws permutations of the original confounder variable that comply with the GAM-based conditional distribution (permutation 1, 2,..., m). The test statistic (coefficient of determination, *R*^2^) is then computed between the model output and the original, as well as the permuted confounder variables. The original and the permuted test statistics construct the *P* value as the ratio of permuted test statistics more extreme than the original. Figure source code available as jupyter notebook: https://github.com/pni-lab/mlconfound-manuscript/blob/main/simulated/overview-fig.ipynb.

In the following section, CPT is adapted for *partial* confounder testing and extended with the general additive model [48] (GAM) and multinomial logistic regression [49,50] based conditional distribution estimations, in order to make it handle categorical data and nonlinear dependencies between the confounder and the target variable. (For an overview of the method, see Fig. 2.)

### The partial confounder test

The inner workings of the *partial confounder test* are summarized in Fig. 2. In short, the test models the conditional distribution between the confounder and the target variable with a GAM—or with an *mnlogit* regression, in case of a categorical confounder—and then uses a so-called parallel-pairwise Markov chain Monte Carlo sampler of Berrett et al. [36] that draws permutations of the original confounder, so that the permuted variables still comply with the estimated conditional distribution. As a result, the permuted “copies” of the confounder variable retain its correlation with the target variable but eliminate any “additional” relationship with the model output. The test statistic (coefficient of determination, *R*^2^) is then computed between the model output and the original, as well as the permuted confounder variables. The original and the permuted test statistics construct the *P* value as the ratio of permuted test statistics more extreme than the original.

In detail, the partial confounder test generates a null distribution for an arbitrary predefined test statistic $T(\boldsymbol{y},\boldsymbol{\hat{y}},\boldsymbol{c})$ by sampling permutation based “copies” of the original $\boldsymbol{c}$,

(1) \begin{equation*}
c_i^{(j)} \sim Q(\cdot |y_i) \end{equation*}

where, *Q*(.|*y*) denotes the conditional distribution of $\boldsymbol{c}$ given $\boldsymbol{y}=y_i$ and *j* = 1, …, *m* indexes the “copy” of $\boldsymbol{c}$ so that

\begin{equation*} \boldsymbol{c}^{(j)} = (c_1^{(j)}, \dots , c_n^{(j)}) = (c_{\pi _1^{(j)}}, \dots , c_{\pi _n^{(j)}}) = \boldsymbol{c}_{\boldsymbol{\pi }^{(j)}}
\end{equation*}

is a permutation of the original vector $\boldsymbol{c}= (c_1, \dots , c_n)$, with its elements reordered according to the permutation $\boldsymbol{\pi } \in S_n$, where *S_n_* denote the set of all permutations on the indices {1, …, *n*}.

As shown by Berrett et al. [36], to ensure that Equation 1 holds, the $\boldsymbol{c}_{\boldsymbol{\pi }^{(j)}}$ copies must be drawn so that

(2) \begin{equation*}
\mathbb {P}(\boldsymbol{ \pi }^{(j)} = \boldsymbol{ \pi } | \boldsymbol{y},\boldsymbol{\hat{y}},\boldsymbol{c}) = \frac{q^n(\boldsymbol{c}_{\boldsymbol{ \pi }} | \boldsymbol{y})}{\sum _{\boldsymbol{ \pi } ^{\prime } \in S_n} q^n(\boldsymbol{c}_{\boldsymbol{ \pi } ^{\prime } } | \boldsymbol{y})}
\end{equation*}

that is, according to the $q^n(\cdot |\boldsymbol{y}) := q(\cdot | y_1) \dots q(\cdot |y_n)$ product density corresponding to the conditional distribution $Q(\cdot |\boldsymbol{y})$. Note that Equation 2 does not necessarily assume a continuous distribution.

This mechanism creates copies $\boldsymbol{c}^{(1)}, \dots ,\boldsymbol{c}^{(m)}$ so that under the null hypothesis ($\boldsymbol{\hat{y}}{\perp\!\!\!\perp} \boldsymbol{c}| \boldsymbol{y}$), the triples

\begin{equation*} (\boldsymbol{y},\boldsymbol{\hat{y}},\boldsymbol{c}), (\boldsymbol{y}, \boldsymbol{\hat{y}}, \boldsymbol{c}^{(1)}),\dots , (\boldsymbol{y}, \boldsymbol{\hat{y}}, \boldsymbol{c}^{(m)}) \end{equation*}

are all identically distributed and so are the

\begin{equation*} T(\boldsymbol{y},\boldsymbol{\hat{y}},\boldsymbol{c}), T(\boldsymbol{y}, \boldsymbol{\hat{y}}, \boldsymbol{c}^{(1)}),\dots ,T(\boldsymbol{y}, \boldsymbol{\hat{y}}, \boldsymbol{c}^{(m)}) \end{equation*}

test statistics, as well.

As long as the numerator of Equation 2 is nonzero for all *c*_π_ ∈ *C* and *y* ∈ *Y*, the conditional permutations constitute an algebraic group; thus, as shown by Hemerik and Goeman [42], an unbiased estimate of the *P* value under the null can be obtained as

\begin{equation*} p= \frac{\sum _{j=1}^m \mathbb {1} \lbrace T(\boldsymbol{y}, \boldsymbol{\hat{y}}, \boldsymbol{c}^{(j)}) \ge T(\boldsymbol{y}, \boldsymbol{\hat{y}}, \boldsymbol{c}) \rbrace }{m}
\end{equation*}

While the group property of the conditioned permutations provides a straightforward proof for the validity of the approach, for an alternative verification, see the proof of Theorem 1 in Berrett et al. [36].

The required permutations could be theoretically sampled with a simple Metropolis–Hastings algorithm that draws uniformly from *S_n_* at random. However, this way, the acceptance ratio would be extremely low, even for moderate *n* (except there is very low dependence of $\boldsymbol{c}$ on $\boldsymbol{y}$), resulting in slow mixing times. The partial confounder test can be, however, efficiently implemented with the parallelized pairwise Markov chain Monte Carlo sampler of Berrett et al. [36] (Algorithm 1), which draws disjoint pairs in parallel and decides whether or not to swap them randomly, according to the odds ratio calculated from the conditional densities belonging to the original and swapped data. The acceptance odds ratio of swapping indices *i* and *j* is

(3) \begin{equation*}
\ln \frac{ q(c_j | y_i) q(c_i | y_j)}{q(c_i | y_i) q(c_j | y_j) } = \ell (c_j | y_i) + \ell (c_i | y_j) - \ell (c_i | y_i) - \ell (c_j | y_j) \end{equation*}

where ℓ denotes the log-likelihood.

In their Theorem 2, Berrett et al. [36] verify that the resulting Markov chain yields the desired stationary distribution, even if the number of steps is small.

#### Conditional log-likelihood

Obtaining a relatively accurate, independent estimate of $Q(\cdot |\boldsymbol{y})$ (of any shape) for CPT inference is important. Berrett and colleagues [36] recommend using a large independent sample to obtain the log-likelihood matrix that represents the conditional distribution *Q*( · |*Z*) or, alternatively, to reuse the data by fitting a least squares linear regression:

(4) \begin{equation*}
\boldsymbol{c}= \alpha + \beta \boldsymbol{y}+ \boldsymbol{e}
\end{equation*}

As the linear regression-based method, obviously, does not handle nonlinear relationships, I propose to apply a modeling approach that accounts for nonlinearity. Although several nonparametric techniques might be suitable for this purpose, many of these tend to be greedy for large sample sizes, may lack stability, or perform poorly with many potential predictors. Certain methods, such as kernel methods and smoothing splines, are also very difficult to interpret [51]—an important consideration when analyzing the source of a confounder effect.

Here, I propose to use the GAM of Hastie and Tibshirani [48]:

(5) \begin{equation*}
\boldsymbol{c}= \alpha + \beta f(\boldsymbol{y}) + \boldsymbol{e}
\end{equation*}

where the feature function *f* is built using penalized B-splines, which allow us to automatically model nonlinear relationships without having to manually try out many different transformations on each variable. The principal advantages of GAM are that (i) the complexity of the model can be effectively regularized trough its hyperparameters, (ii) it is able to model highly complex nonlinear relationships with a potentially large number of both numeric and categorical predictors, and (iii) it has computationally effective solver algorithms. The potential disadvantages of GAMs are not relevant for the problem at hand or can be easily overcome. Specifically, the possibly poor out-of-distribution generalization of GAM is not problematic, as in our approach, the model is not used for constructing out-of-distribution predictions. Moreover, as several other models, GAMs can easily overfit the data. However, in the proposed approach, the smoothness of the GAM model is optimized with a grid search by picking the model with the lowest generalized cross-validation score from the models defined by the default parameters as implemented in PyGAM [52] (v0.8.0).

If we write $\boldsymbol{\mu } = \alpha + \beta f(\boldsymbol{y})$ and σ denotes the standard deviation of the residual $\boldsymbol{e}$, then the conditional distribution of interest can be assumed to be normal with the parameters

\begin{equation*} (\boldsymbol{c}|\boldsymbol{y}=y_i) \sim \mathcal {N}\lbrace \mu _i, \sigma ^2\rbrace
\end{equation*}

and the log-likelihood, which is to be used in Equation 3, can be computed simply as the log of the corresponding probability density function:

\begin{equation*} \ell (c_i|y_j) = - \frac{1}{2} \Big (\frac{c_i-\mu _j}{\sigma }\Big )^2 - ln(2 \pi \sigma ) \end{equation*}

In the case of categorical $\boldsymbol{c}$, a multinomial logistic regression (*mnlogit*) model can be used to obtain $D(\cdot |\boldsymbol{y})$, with the extra assumption of *complete separation* if $\boldsymbol{y}$ is also categorical (in order to ensure an invertible Hessian, see, e.g., [49, 50]).

Importantly, both the GAM- and the *mnlogit*-based approaches guarantee that the numerator of Equation 2 is always greater than zero and the group property for the permutations holds.

Note that from the 3 options for conditional independence-based null hypotheses enumerated in Table 1, the proposed approach cannot provide a test for option 2 that is assumption free about $\boldsymbol{\hat{y}}$, as the variable, on which the independence is conditional, must be always the predictor variable in Equation 5. However, as discussed above, this option is of low practical relevance anyway. Pleasingly, the proposed Gaussian regression-based conditional likelihood estimation ensures that no assumptions on $\boldsymbol{\hat{y}}$ have to be made for the practically relevant options 1 and 3 (i.e., for the full and partial confounder tests).

In theory, any predefined test statistic *T* can be used with the proposed approach. The Python package *mlconfound*, implementing the proposed full and partial confounder tests, utilizes the coefficient of determination (*R*^2^ or pseudo-*R*^2^ in case of categorical confounder or classification [53]) as a test statistic: $T(\boldsymbol{y}, \boldsymbol{\hat{y}}, \boldsymbol{c}) = R^2(\boldsymbol{\hat{y}}, \boldsymbol{c})$ and $T(\boldsymbol{y}, \boldsymbol{\hat{y}}, \boldsymbol{c}^{(j)}) = R^2(\boldsymbol{\hat{y}}, \boldsymbol{c}^{(j)})$, which allows interpretable, 2-tailed inference.

### Validation on simulated data

Using CPT to test confounding bias in predictive modeling allows relaxing assumptions on $\boldsymbol{\hat{y}}$ but—in line with the “no free lunch” theorem, requires knowing—or putting assumptions on—the joint distribution of the other 2 variables ($\boldsymbol{y}$ and $\boldsymbol{c}$). Berrett et al. [36] give a detailed analysis of the robustness of their CPT approach when estimating the conditional distribution with reusing the tested data via linear regression and, also, against misspecifying the conditional distribution of interest to introduce nonlinearity.

Here I extend these results by performing simulations that evaluate the GAM- and *mnlogit*-based approaches, in a form that is accessible for power calculations in predictive modeling (considering various weights of the target signal in $\boldsymbol{c}$ and the confounder and the target signals in $\boldsymbol{\hat{y}}$). Moreover, I investigate the robustness of the tests against the violation of normality and linearity of the conditional distributions $D(\boldsymbol{c}|\boldsymbol{y})$ and $D(\boldsymbol{\hat{y}}|\boldsymbol{y})$.

Simulations are performed separately for the 2 proposed tests.

#### Simulation approach

As a first step, the target variable $\boldsymbol{y}$ is drawn randomly from a normal distribution:

\begin{equation*} \boldsymbol{y}\sim \mathcal {N}(0, 1) \end{equation*}

Next, the confounder signal is simulated as

\begin{equation*} \boldsymbol{c}| y_i \sim f_{\delta , \epsilon }(\mathcal {N}(0, 1)) + w_{yc} \, g(y_i) \end{equation*}

where *f* is a function to introduce nonnormality, namely, the *sinh-arcsinh* transformation of Jones and Pewsey [54], defined as

\begin{equation*} f_{\delta , \epsilon }(\boldsymbol{x}) = sinh(\delta sinh^{-1}(\boldsymbol{x}) - \epsilon ) \end{equation*}

where the parameters δ and ϵ control the kurtosis and skewness of the resulting *sinh-arcsinh* distribution, with δ = 1 and ϵ = 0 producing the identity function (i.e., no nonnormality introduced).

Moreover, nonlinearity can be introduced with the function *g*, which can be simply the identity function (no nonlinearity is introduced in this case) or, for instance, a sigmoid-shaped function, in our case:

\begin{equation*} g(\boldsymbol{x}) = tanh(\boldsymbol{x}) \end{equation*}

The simulated predicted values are constructed in a similar fashion but may depend on $\boldsymbol{c}$ as well:

\begin{equation*} \boldsymbol{\hat{y}}| y_i, c_i \sim f_{\delta , \epsilon }(\mathcal {N}(0, 1)) + w_{y\hat{y}} \: g(y_i) \, + \, w_{c\hat{y}} \: c_i
\end{equation*}

Note that simulations with $w_{c\hat{y}}=0$ produce data under the null hypothesis of no confounding bias.

To test the implementation for categorical variables, simulated $\boldsymbol{y}$, $\boldsymbol{\hat{y}}$, and $\boldsymbol{c}$ variables are binarized by thresholding at 0.

#### Simulations for comparison with partial Spearman correlation and linear CPT

**Figure 3:**
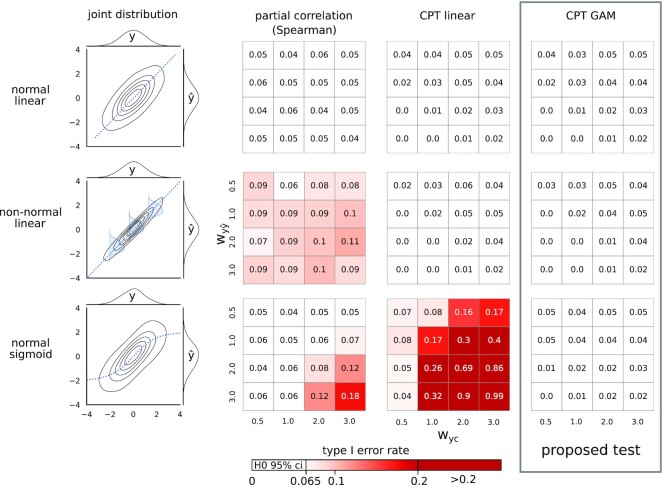
: Type I error control of partial Spearman correlation, linear and GAM-based conditional permutation test. Type I error control was investigated in 3 example cases: normal conditional distribution with linear dependency (first row), slightly nonnormal conditional distribution with linear dependency (second row), and normal conditional distribution with nonnormal (sigmoid) dependency (third row). Nonnormal conditional distribution on the second plot is illustrated with blue density diagrams (kurtosis: –0.8, skewness: –0.1). False-positive rates for confounder contributions (*w_yc_*, ranging from 0.5 to 3.0) and predictive performances ($w_{y\hat{y}}$, ranging from 0.5 to 3.0) are shown in heatmaps. The upper limit for the binomial confidence interval corresponding to alpha = 0.05 is 0.065. Values below this threshold (colored white) indicate a valid type I error control.

To demonstrate the need for the proposed GAM-based CPT approach for partial confounder testing (Fig. 3), its validity was contrasted to partial Spearman correlation and the linear variety of CPT (based on Equation 4, as described by Berrett et al. [36]) with the following simulation parameters: sample size *n* = 1,000, $w_{c\hat{y}} = 0$ (i.e., H0 simulations only), taking all combinations of *w_yc_* ∈ {0.5, 1, 2, 3} and $w_{y\hat{y}} \in \lbrace 0.5, 1, 2, 3\rbrace$. Furthermore, simulations cases with nonnormality (*f*_δ = 0.1, ϵ = 2_) and nonlinearity (sigmoid *g*) have also been investigated for all simulation cases.

For each parameter combination, 1,000 repetitions were performed and false-positive rates were calculated as the ratio of *P* values smaller than α = 0.05.

The simulation cases are exemplified (with $w_{yc} = w_{y\hat{y}} = 2$) on the left of Fig. 3.

#### Simulations for evaluating power

One hundred repetitions were performed of all combinations of the following parameter values: *w_yc_* ∈ {0.5, 1, 2, 3}, $w_{y\hat{y}} \in \lbrace 0.5, 1, 2, 3\rbrace$, $w_{c\hat{y}} \in \lbrace 0, 0.2, 0.4, 0.6\rbrace$, *n* ∈ {50, 100, 500, 1,000}. All simulations were performed with both linear and sigmoid dependence as well as with normal and nonnormal conditional distributions: (δ, ϵ) = {(0.1, 2), (1, 0), (1.05, −3), (1.5, −5), (5, −10)}.

The partial confounder tests, as implemented in version 0.20.0 of the package “*mlconfound*,” were run with default parameters (1,000 permutations and 50 Markov chain Monte Carlo steps to generate the conditioned permutations) and by implying categorical variables, where needed.

All code used for the simulations is available on GitHub (https://github.com/pni-lab/mlconfound-manuscript/tree/main/simulated).

### Application on functional brain connectivity data

The usefulness of the proposed confounder tests is demonstrated by applying them for predictive classification and regression models based on functional brain connectivity data, processed with different confound mitigation approaches.

Partial confounder testing was performed with 10,000 permutations and 50 Markov chain Monte Carlo steps, as implemented in version 0.20.0 of the package “*mlconfound*.” Unconditional dependence across the involved variables was investigated with conventional permutation tests on the *R*^2^ values, with 1,000 permutations.

All empirical analyses are available as jupyter notebooks on GitHub (https://github.com/pni-lab/mlconfound-manuscript/tree/main/empirical).

#### HCP: testing age and acquisition batch bias in fluid intelligence prediction

The Human Connectome Project dataset contains imaging and behavioral data of approximately 1,200 healthy subjects [55]. Preprocessed resting state functional magnetic resonance imaging (fMRI) connectivity data (partial correlation matrices) [56] as published with the HCP1200 release (*N*= 999 participants with functional connectivity data) were used to build models that predict individual fluid intelligence scores (*G_f_*), measured with Penn Progressive Matrices [57].

To ensure normality of the target variable for the partial correlation-based analyses, *G_f_* was nonlinearly transformed to normal distribution with the quantile transformation [58] as implemented in *scikit-learn* [59] (see Supplementary Fig. S8 for details).

Features (functional connectivities across 100 group-independent component analysis–based regions) were either (i) considered in their raw form or were subject to confound mitigation approaches by (ii) feature regression [9] or (iii) COMBAT [28, 60]. The feature mitigation strategies were separately applied for acquisition batch and age group as confounder variable.

Each of the 5 types of features (raw, regressing out acquisition batch, regressing out age group, COMBAT with acquisition batch, COMBAT with age group) was independently incorporated into a *scikit-learn*–based [59] machine learning procedure aiming to predict the individual fluid intelligence scores with a ridge regression [61]. The α parameter of the ridge model was considered a hyperparameter (α ∈ {0.00001, 0.0001, 0.001, 0.01, 0.1, 1, 10, 100, 1000, 10000, 100000}) and optimized in a nested cross-validation with 10 folds in both the inner and the outer loops and with mean squared error as an optimization metric. Confound mitigation was performed inside of the outer cross-validation loop, to avoid leakage.

#### ABIDE: testing motion and center bias in predictive models of ASD diagnosis

The proposed tests were applied to provide evidence of center and motion bias in diagnostic predictive models of ASD, trained on the Autism Brain Imaging Data Exchange (ABIDE) dataset [62] involving 866 participants (ASD: 402, neurotypical control: 464). Preprocessed regional time-series data were obtained as shared (https://osf.io/hc4md) by Dadi et al. [63], which were based on preprocessed image data provided by the Preprocessed Connectome Project [64].

Tangent correlation across the time series of the *n*= 122 regions of the BASC (Multi-level bootstrap analysis of stable clusters) [65] brain parcellation was computed with nilearn (http://nilearn.github.io/) [66,67].

The resulting functional connectivity estimates were considered features either (i) in their raw form or after applying (ii) feature regression [9] or (iii) COMBAT [28, 60]. The investigated confounder variables were “imaging center” and “in-scanner motion,” as measured by the mean framewise displacement (FD), as defined by Power et al. [68]. Mean FD was nonlinearly transformed to normal distribution with the quantile transformation [58] as implemented in *scikit-learn* [59] (see Supplementary Fig. S9 for details).

As COMBAT is not able to handle continuous variables (since it was primarily designed to remove categorical “batch effects”), motion was binned into 10 groups, based on equidistant data quantiles ranging from 0 to 1.

A total of 5 types (raw, feature regression of site, feature regression of motion, COMBAT with site, COMBAT with motion) of features were independently incorporated into a *scikit-learn*–based [59] machine learning procedure aiming to predict the diagnosis (DX: ASD vs. neurotypical controls) with an L2-regularized logistic regression, as previously recommended [63]. The *C* parameter of the model was considered a hyperparameter (*C* ∈ {0.1, 1, 10}) and optimized in a nested cross-validation with 10 folds both in the inner and the outer cross-validation loop and with area under the receiver operator curve (AUC under ROC) as the optimization metric. Confound mitigation was performed inside of the outer cross-validation loop, to avoid leakage. Confounder testing was performed on the predicted class probabilities.

## Results

### Partial confounder tests

The proposed *partial confounder tests* have been implemented in the Python package *mlconfound*(https://mlconfound.readthedocs.io) (biotools:mlconfound, RRID:SCR_022545).

### Simulations

#### Type I error

As suggested by theory (see Methods for details) and shown by the simulations with a wide range of settings, both of the proposed tests provide a valid type I error control (Fig. 4 and Supplementary Figs. S1–S3), even in case of nonlinearity and nonnormality (Figs. 3, 5 and Supplementary Figs. S4–S7), except when nonnormality is extreme (purple distribution on Fig. 5, kurtosis: 42, skewness: –6).

**Figure 4:**
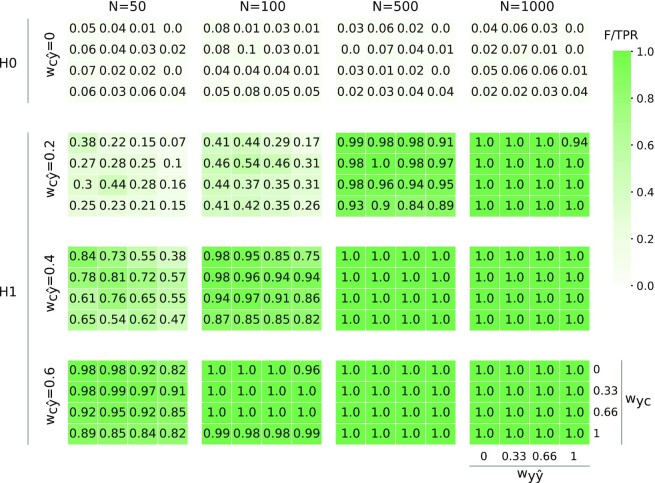
The partial confounder test provides a strict control for type I errors and a high statistical power in simulated data. Heatmaps depict positive rates (ratio of *P* values lower than 0.05, color coded as shown by the palette on the right) in various simulation settings (100 simulations per tile) with different simulation weights $w_{y\hat{y}}$ (predictive performance; horizontal axis on each heatmap), *w_yc_* (confounder–target association; vertical axis on each heatmap), $w_{c\hat{y}}$ (degree of confounder bias; rows), and for different sample sizes (*N*, columns). Weights 0.2, 0.33, 0.4, 0.6, 0.66, and 1.0 can be assigned to the following approximate explained variance values: 4%, 10%, 12%, 25%, 30%, and 50%, respectively. First row contains simulations under the null hypothesis (H0, no confounding bias), and rows 2–4 represent simulations from the alternative hypothesis (H1, confounding bias). Positive rates for the simulations under the null and the alternative hypotheses can be interpreted as type I error rate and statistical power, respectively. The higher 95% confidence limit for a positive rate of alpha = 0.05 is 0.11 for each tile.

**Figure 5:**
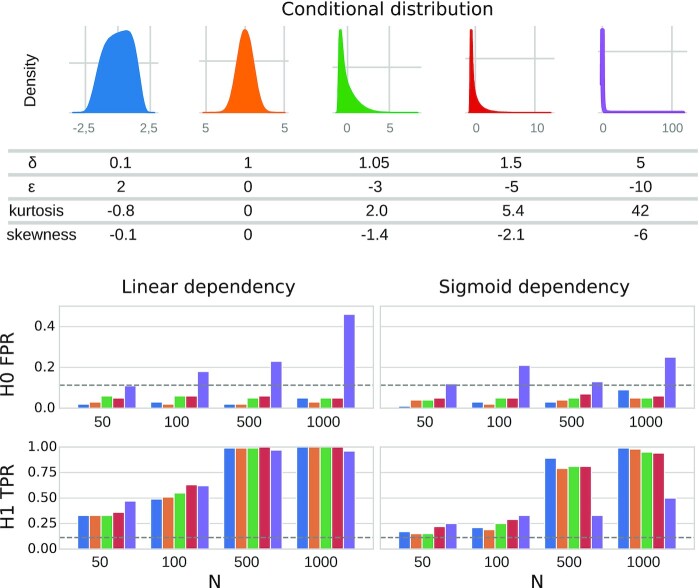
The partial confounder test is robust to nonnormality and nonlinearity. Simulations included variables with 5 different degrees of nonnormality (top panel), as introduced with various δ and ϵ values of the *sinh-arcsinh* transformation (yellow: normally distributed). Fisher’s kurtosis and skewness are given for each distribution. False- and true-positive rates in the simulations under H0 and H1, respectively, for each investigated sample size (*N*), are depicted by barplots for both linear and sigmoid dependency structure. Upper 95% binomial confidence limit corresponding to alpha = 0.05 is shown with a vertical dashed line.

#### Power

Estimates of statistical power for the partial confounder test (with normal and linear simulations, for a wide range of parameters) were found to be virtually identical to those of Pearson’s partial correlation (see Fig. 4 and Supplementary Fig. S12). Notably, with sample sizes as large as 1,000, a confounder contributing only $\sim 4\%$ to the variance of the predictions ($w_{c\hat{y}} = 0.2$) can already be robustly detected with a power of 94–100%. With a sample size of 500, the same confounding bias is still detected with a power greater than 84–100% in all of the simulation cases. A sample size of 100 requires a somewhat stronger bias with approximately 12% of explained variance ($w_{c\hat{y}}=0.4$) to achieve a reasonable level of power (75–98%). Finally, even with a relatively low sample size of 50, the same amount of confounder variance is detected with a power of at least 50%. If the confounder explains more than 25% of variance, it is almost certainly detected even with a low sample size of *n* ≥ 50.

Simulations show that nonnormality has a minimal effect on the power of the tests, except in case of extreme nonnormality (Fig. 5). Simulations with sigmoid dependence resulted in an apparent loss of statistical power, but this is simply a consequence of the simulation methodology: with the same parameters, the sigmoid-transformed confounder explains only approximately half the variance as compared to linear simulations. Type I error control was valid in case of categorical variables, as well (Supplementary Figs. S1, S3, S5, S7).

### Neuroimaging data

To demonstrate the usefulness of the proposed tests in detecting various types of confounding bias, they have been deployed in 2 typical research scenarios—a regression and a classification problem—where confounder effects are known to hamper the development of biomedically useful predictive models. The empirical analyses confirmed the presence of nonlinearity and nonnormality in the output of the predictive models (see Supplementary Fig. S11 for more details).

#### HCP dataset

Functional connectivity data from the Human Connectome Project [55] (HCP) were used to build predictive models of fluid intelligence (*G_f_*) and to test for the previously discussed confounding effect of age [18, 19] and, additionally, the—somewhat underdiscussed—batch-like effect of acquisition date of the data within the course of the data acquisition process.

Both acquisition batch and age group were statistically significantly associated with *G_f_* (*R*^2^ = 0.032 and 0.011 and *P* < 0.001 and *P* = 0.001, respectively; see also Table 2). The model trained on the raw (unadjusted) connectivity features predicted fluid intelligence with a medium effect size (*R*^2^ = 0.095, *P* < 0.001).

**Table 2.** Coefficients of determination (*R*^2^), the corresponding *P* values, and the *P* values of the partial confounder tests, for all investigated datasets, confounders (conf.), and confounder mitigation methods (method). Bold numbers denote significant confounding bias identified by the partial confounder test.

Dataset	Conf.	Method	$R^2_{y, c}$	*p_y, c_*	$R^2_{\hat{y}, c}$	$p_{\hat{y}, c}$	$R^2_{\hat{y}, y}$	$p_{\hat{y}, y}$	Partial confounder test
HCP	acq.	raw	0.032	<0.001	0.071	<0.001	0.095	<0.001	**<0.0001**
		f.reg.			0.0	1.0	0.114	<0.001	1.0
		COMBAT			0.013	0.4	0.122	<0.001	0.65
	age	raw	0.011	0.001	0.034	<0.001	0.095	<0.001	**<0.0001**
		f.reg.			0.0	0.92	0.118	<0.001	0.95
		COMBAT			0.005	0.048	0.121	<0.001	0.16
ABIDE	center	raw	0.019	<0.001	0.169	<0.001	0.126	<0.001	**<0.0001**
		f.reg.			0.004	1.0	0.179	<0.001	1.0
		COMBAT			0.05	0.001	0.17	<0.001	**0.009**
	motion	raw	0.028	<0.001	0.111	<0.001	0.126	<0.001	**<0.0001**
		f.reg.			0.002	0.16	0.098	<0.001	0.51
		COMBAT			0.002	0.19	0.111	<0.001	0.59

**Figure 6:**
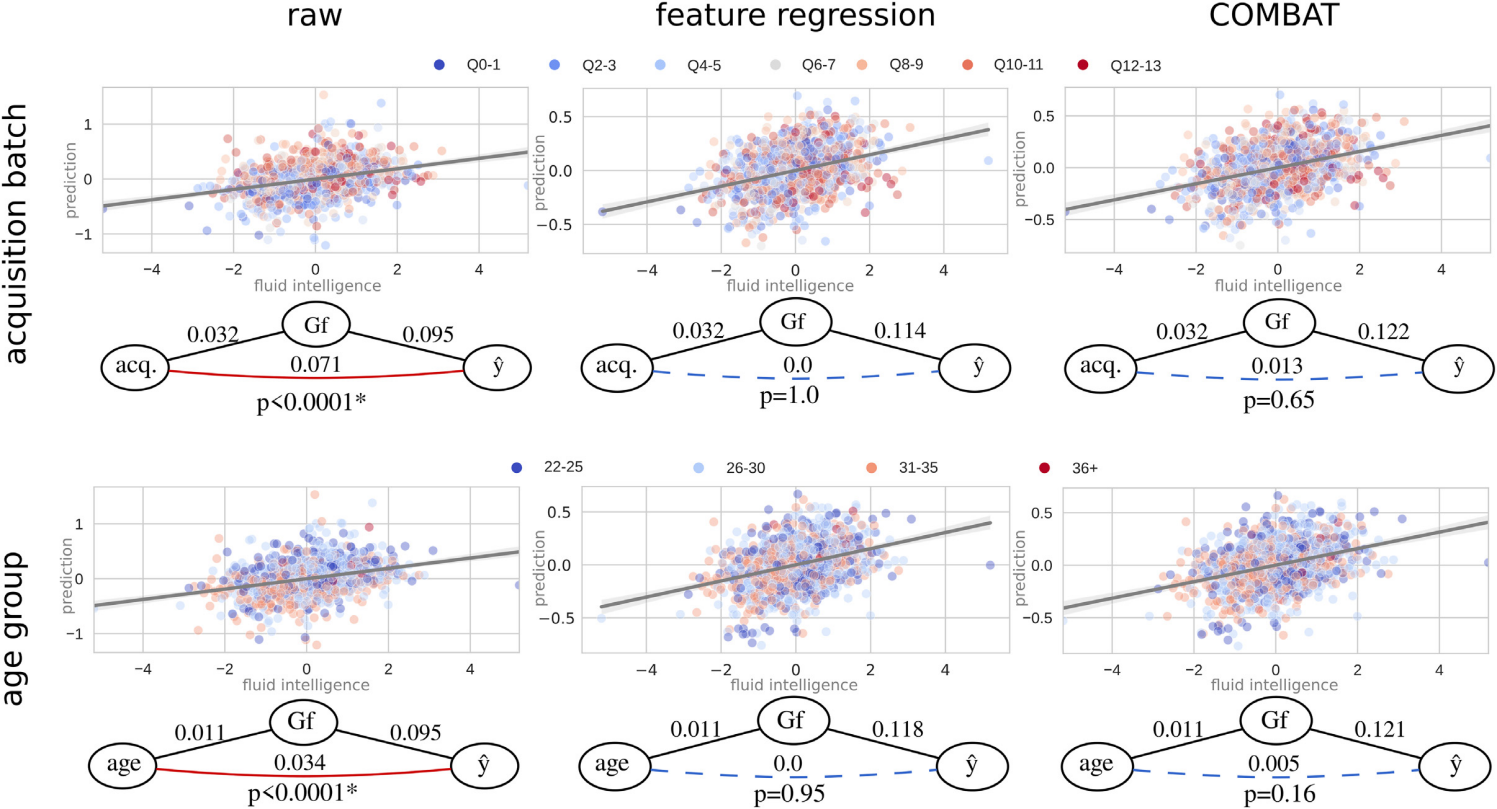
: The partial confounder test reveals that acquisition batch and age bias in predictive models of fluid intelligence can be effectively attenuated by confounder mitigation approaches in the HCP dataset. Scatterplots and regression lines (with 95% confidence intervals) show the association of the observed (horizontals axis) and predicted (vertical axis) fluid intelligence scores with various confound regression strategies. Color-coding of the confounder variables (top: acquisition batch, bottom: age group, as shown by the corresponding legends) reveals confounding bias for both acquisition and age in the models trained on the raw data. This bias is robustly detected by the partial confounder test (*P* < 0.0001) and seems to be effectively mitigated by both feature regression and COMBAT. Relation between the observed (*G_*f*_*) and predicted ($\hat{y}$) intelligence scores and the confounder variables is given on the graphs via *R*^2^ values. Both confound mitigation techniques, but especially COMBAT, improve the predictive performance. Solid red line between the confounder and the prediction means significant confounding bias, whereas blue dashed line denotes that confounder testing provided no evidence for bias. *P*values are determined with the partial confounder test.

The partial confounder test revealed that the “raw” model (without confounder mitigation) was significantly biased by both age group and acquisition batch (both *P* < 0.0001, first column of Fig. 6), with later phases of the acquisition and lower age being associated with larger predicted values.

After applying confound mitigation approaches (feature regression or COMBAT), the partial confounder test did not provide evidence for confounding bias anymore (*P* > 0.05 for all; shown in the second and third columns of Fig. 6), neither for acquisition batch nor for age. Both feature regression and COMBAT increased the predictive performance, with COMBAT providing the overall best performances (*R*^2^ = 0.122 and 0.121 when applied to remove the effect of acquisition and age, respectively).

#### ABIDE dataset

Functional connectivity data from the ABIDE [62] database were used to investigate the potential motion and center bias (as previously reported, e.g., by [13,14] or [12]) when training models that aim to predict ASD diagnosis.

Imaging center and in-scanner motion (normalized mean framewise displacement) were statistically significantly associated with ASD diagnosis (*R*^2^ = 0.019 and 0.028, respectively; *P* < 0.001 for both; see also Table 2). The model trained on the raw (unadjusted) connectivity features predicted diagnosis with a medium effect size (*R*^2^ = 0.126, ROC AUC = 0.71, *P* < 0.001).

**Figure 7:**
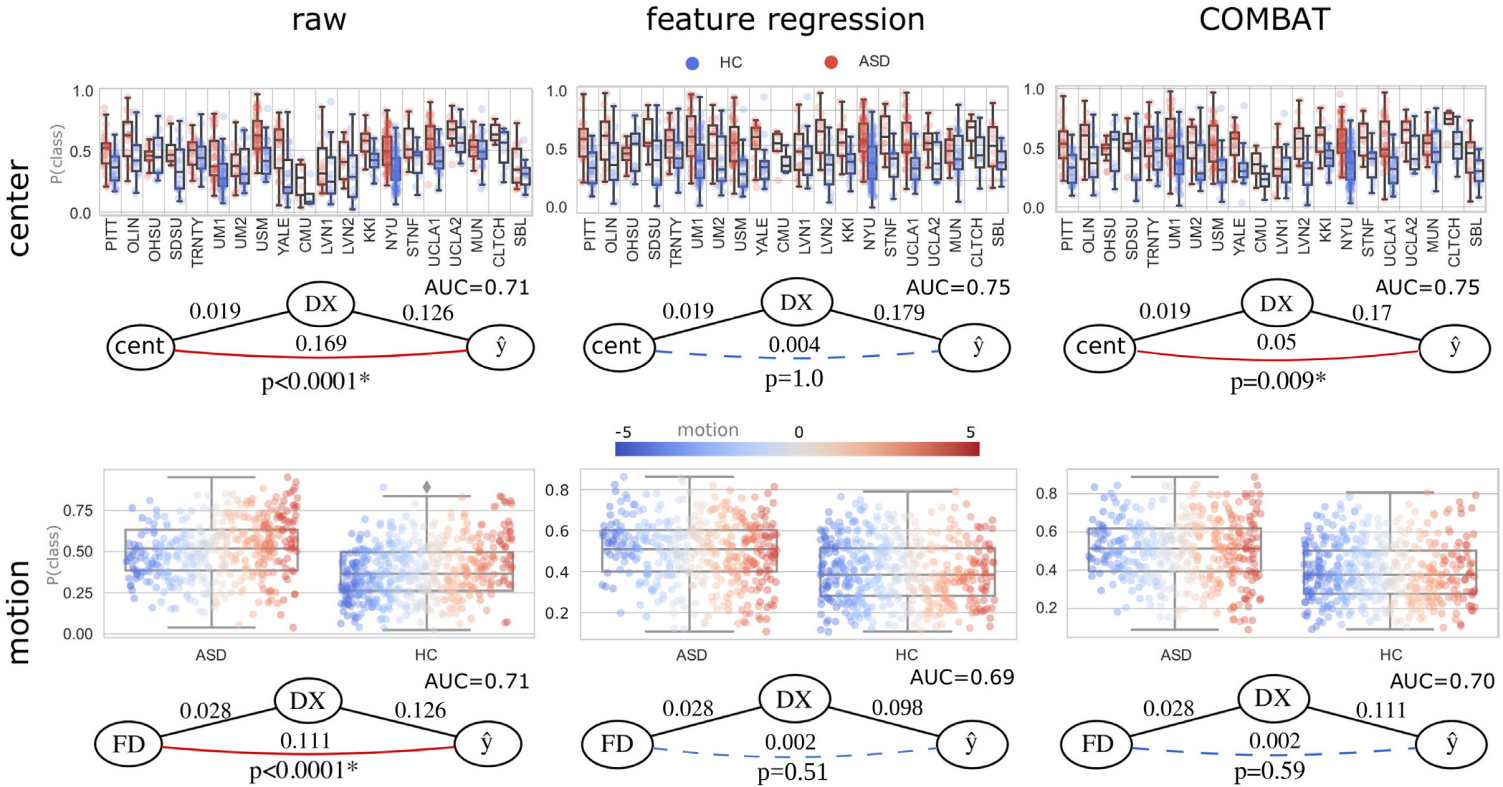
: The partial confounder test identifies an efficient mitigation strategy for motion bias in predictive models of autism spectrum disorder and reveals residual center bias after COMBAT in the ABIDE dataset. Boxplots and points show the predicted class probabilities (0: Healthy Control (HC), 1: ASD), separately for the HC and ASD groups. In the top panel, predictions are plotted for each center separately. Color indicates the true diagnosis (DX). At the bottom plot, color indicates the normalized index of in-scanner motion (normalized FD). The proposed confounder test reveals significant center and motion bias in the model trained on the raw data (*P* < 0.0001). While both motion and center bias were effectively mitigated by both feature regression and COMBAT, the proposed partial confounder test revealed COMBAT was not able to fully remove center bias and resulted in significant “residual bias” (*P*< 0.05). Relation between the true ($\hat{y}$) and predicted diagnosis scores and the confounder variables is shown by the graphs as *R*^2^ values. Solid red line between the confounder and the prediction means significant confounding bias, whereas blue dashed line denotes that confounder testing provided no evidence for bias. *P*values are determined with the partial confounder test.

The partial confounder test revealed that the raw model was significantly biased for both age group and acquisition batch (both *P* < 0.0001; see first column in Fig. 7). Predictions for several sites (e.g., Carnegie Mellon University, University of Leuven, Social Brain Lab UMC Groningen) were severely miscalibrated, and higher motion was associated with a higher probability for ASD diagnosis.

Both feature regression and COMBAT seemed to significantly attenuate center bias, but with COMBAT, the partial confounder test still provided evidence for a significant residual bias (0.009, third columns of the first row in Fig. 7).

When trying to mitigate the effect of in-scanner motion (bottom row in Fig. 7), both confounder mitigation approaches seemed to effectively mitigate motion bias, as suggested by the partial confounder test (*P* > 0.05, middle and right panels in the bottom row of Fig. 7).

Both feature regression and COMBAT considerably improved the predictive performance when mitigating center effects (AUC = 0.71 without correction and 0.75 with both feature regression and combat). With both feature regression and COMBAT, however, the effort of mitigating motion effects happened at the cost of a drop in predictive performance (AUC = 0.69 and 0.70, for feature regression and COMBAT, respectively).

## Discussion

The concept of conditional independence provides a straightforward framework for assessing confounding bias in predictive models, assuming that both the target variable and the potential confounder have been observed for the validation dataset. However, handling the nonnormal and/or nonlinear conditional dependencies often seen in predictive models [37, 38] (Supplementary Figs. S10–S11) poses a great challenge. In fact, as recently shown by Shah and Peters in their “no free lunch” theorem [35], it is effectively impossible to establish a *fully nonparametric* conditional independence test with a valid type I error control and a nontrivial power. Indeed, perhaps somewhat surprisingly, but not totally unexpectedly [31], partial correlation–like analogs of a widely used bivariate nonparametric test, like partial Spearman correlation, exhibit inflated type I errors even with slight violations of normality and/or linearity (as clearly demonstrated with simulated data in Fig. 3). While the magnitude of this problem may not be fully appreciated in case of predictive model diagnostics, such tests are, in general, poor choices for testing confounding bias in machine learning. Conditional independence-based confounding bias testing must, therefore, be designed so that its suitability for the particular problem may be judged easily.

These tests place no assumptions on the conditional distributions of the model output, ensuring valid model diagnostics even in cases of nonnormally and nonlinearly dependent predictions. This property distinguishes the approach from other alternatives as it guarantees a valid type I error control even in cases of nonnormally and nonlinearly dependent predictions (i.e., in cases where Pearson and Spearman partial correlations and many other methods fail).

The proposed tests are based on solid theoretical foundations, underpinned by mathematical proofs. The main purpose of the simulated and empirical experiments was, therefore, not to justify the validity of the approach but to (i) test the software implementation, (ii) estimate statistical power in various situations, and (iii) exemplify how the partial confounder test can be used with real experimental data. The validity of the type I error control and was confirmed by our simulations, even if both the predictions and the confounder are nonnormally and/or nonlinearly dependent on the target variable (except by extreme nonnormality). While different biomedical applications may consider different amounts of bias to be relevant, in most cases, it is possible to set an upper bound for confounding bias that is still tolerable in certain applications. The simulation results can serve as a basis for power calculations in these cases, in order to identify the necessary sample size for proper model diagnostics.

A characteristic example for the potential areas of applications is the novel field of population neuroscience, where applying predictive modeling and machine learning on large-scale functional neuroimaging data holds great potential for both revolutionizing our understanding of the physical basis of mind and delivering clinically useful tools for diagnostics or therapeutic decision-making [3, 5, 23, 25]. However, the presence of confounders that are typical for biomedical research (e.g., sample demographics, center effects) or specific to the data acquisition and processing approach (e.g., imaging artifacts) presents a great challenge to these efforts [29]. The usefulness of the proposed tests is demonstrated in 2 such examples, using the HCP [55] and the ABIDE [62] datasets.

In the case of the HCP dataset, the statistically significant age bias of the “raw” model for predicting fluid intelligence is in line with previous findings [18, 19] and could likely exaggerate to a serious bias when testing the model on data of participants outside of the—relatively narrow—age range of the HCP sample. In this case, the bias would likely significantly harm the out-of-sample generalizability of this model. The bias of the same model for acquisition batch can also be problematic, especially as it has not yet been thoroughly discussed in case of the HCP dataset. There can be manifold reasons for the observed acquisition bias. Fluid intelligence of the included participants might be, for instance, affected by a changing selection bias during participant recruitment (e.g., as a consequence of the HCP receiving an increasing degree of public interest during its course).

In the ABIDE dataset, neither the center bias nor the age bias is surprising in the case of the “raw” model, but both would be obviously severely problematic for a diagnostic biomarker candidate of ASD. For instance, the model trained on the raw (unadjusted) features—depending on the calibration of the predicted class probabilities—might classify all participants from, for example, the Carnegie Mellon University center, as neurotypical control participants. Similarly, the models biased by motion—next to having questionable neuroscientific validity—might systematically fail in populations with a tendency for higher in-scanner motion (as known for many conditions, among others, attention-deficit/hyperactivity disorder [10] or Alzheimer’s disease [9]).

The partial confounder test provided quantitative, statistically rigorous metrics for assessing the effectiveness of the investigated confounder mitigation techniques. In the HCP data, it revealed that both the acquisition bias and the age bias were very effectively removed by both feature regression and COMBAT (*P*> 0.05 for all). Given the high power of the test at the sample sizes of the HCP dataset (*N* = 999), any remaining confounding bias is most probably very safely negligible and well out of the range of practical relevance.

The confound mitigation approaches performed well in attenuating motion bias in the ABIDE dataset, as well, as no residual bias was detected by the proposed test. However, the success of COMBAT in eliminating motion bias is not to be taken without any objections. As COMBAT was originally developed for harmonizing effects of categorical variables (e.g., center or batch), its application for continuous confounder variables is not trivial. Inputting discretized versions of continuous variables into COMBAT might be suboptimal and raises further questions, for example, regarding the optimal number of bins used during the discretization.

Importantly, the partial confounder test revealed that the center bias of the classification in the massively multicenter ABIDE dataset was, although mitigated, not successfully removed by COMBAT. While determining the relevance of the remaining bias is out of the scope of this article, the example demonstrates the need for checking confounder bias even if state-of-the-art confounder mitigation approaches have been applied. If the proposed test provides evidence for residual confounding bias, the researcher might consider the use of another mitigation approach (e.g., feature regression in the given case) or the evaluation of confound-free performance (e.g., via “confound-isolating cross-validation”) [29].

In sum, the application of the partial confounder test on the real data examples suggests that confounding bias must be always carefully investigated and reported in studies utilizing predictive modeling and machine learning as (i) variables as trivial as the date of the acquisition can cause significant confounding bias, and (ii) in certain situations, state-of-the-art confounder mitigation techniques may not provide sufficient mitigation of confounding bias, and (iii) unnecessary confounder correction may eliminate variance of interest. As the proposed test is a model-agnostic post hoc test, it can be used to benchmark different machine learning models and to further characterize already trained models in external validation samples, where a larger set of potential confounder variables is available (Supplementary Fig. S13). The partial confounder test can be considered a useful, objective benchmark to guide the search for a suitable confounder mitigation approach for every dataset.

## Conclusion

The lack of rigorous statistical tests for confounding bias significantly hampers the development of predictive models in many fields of research, including population neuroscience, where handling confounding effects is especially challenging [23].

To fill this critical gap in predictive model development, here I proposed 2 novel tests, the *partial* and the *full confounder tests*, which probe the null hypotheses of “no confounding bias” and “full confounding bias,” respectively. The tests are distinguished from alternative approaches by their robustness to nonnormally and nonlinearly dependent predictions, rendering them applicable with a wide variety of machine learning models. The tests have, moreover, a minimal computational overhead, as refitting the model is not required.

As demonstrated on functional brain connectivity-based predictive models of fluid intelligence and ASD, the tests can guide the optimization of confound mitigation strategies and allow quantitative statistical assessment of the robustness, generalizability, and neurobiological validity of predictive models in biomedical research. Given their simplicity, robustness, wide applicability, high statistical power, and computationally effective implementation (available in the Python package *mlconfound*; https://mlconfound.readthedocs.io), the partial and full confounder tests emerge as novel tools in the methodological arsenal of predictive modeling and may largely accelerate the development of clinically useful machine learning biomarkers.

## Data Availability

Empirical analysis was based on preprocessed data provided by the Human Connectome Project, WU-Minn Consortium [55] (principal investigators: D. Van Essen and K. Ugurbil; 1U54MH091657), funded by the 16 National Institutes of Health (NIH) institutes and centers that support the NIH Blueprint for Neuroscience Research, and by the McDonnell Center for Systems Neuroscience at Washington University and the ABIDE consortium [62].

All data used in the present study are available for download from the Human Connectome Project (www.humanconnectome.org). Users must agree to data use terms for the HCP before being allowed access to the data and ConnectomeDB; details are provided at https://www.humanconnectome.org/study/hcp-young-adult/data-use-terms. Python implementation of the “mlconfound” package is available on GitHub. All analysis code is available at GitHub and via the GigaScience database GigaDB [69].

## Additional Files

**Supplemental Figure S1**. Heatmaps showing the positive rates of the “partial” confounder test, with categorical variables, normal conditional distribution, and linear dependence.

**Supplemental Figure S2**. Heatmaps showing the positive rates of the “full” confounder test, with numerical variables, normal conditional distribution, and linear dependence.

**Supplemental Figure S3**. Heatmaps showing the positive rates of the “full” confounder test, with categorical variables, normal conditional distribution, and linear dependence.

**Supplemental Figure S4**. Heatmaps showing the positive rates of the “partial” confounder test, with numerical variables, normal conditional distribution, and sigmoid dependence.

**Supplemental Figure S5**. Heatmaps showing the positive rates of the “partial” confounder test, with categorical variables, normal conditional distribution, and sigmoid dependence.

**Supplemental Figure S6**. Heatmaps showing the positive rates of the “full” confounder test, with numerical variables, normal conditional distribution, and sigmoid dependence.

**Supplemental Figure S7**. Heatmaps showing the positive rates of the “full” confounder test, with categorical variables, normal conditional distribution, and sigmoid dependence.

**Supplemental Figure S8**. Histogram of fluid intelligence score in the HPC dataset, before (left) and after (right) quantile transformation.

**Supplemental Figure S9**. Histogram of mean framewise displacement in the ABIDE dataset, before (left) and after (right) quantile transformation.

**Supplemental Figure S10**. Example of nonlinearity of model predictions as a consequence of regularization. The same 4 (simulated) features may result in nonlinear predictions as the regularization (alpha) of the Ridge model is increased. Model coefficients are shown above the lines connecting the features and the prediction. The full analysis is available at https://github.com/pni-lab/mlconfound- manuscript/blob/main/simulated/normalityandlinearityviolation.ipynb.

**Supplemental Figure S11**. Example of nonnormality of the conditional distributions $\hat{y}|y$ and $\hat{y}|c$. (A) Example from the analysis of the HCP dataset, as presented in the previous version of the manuscript. (B) No evidence of confounder bias with the partial confounder test. (C) Presumably false-positive observations by Pearson’s and Spearman’s partial correlations, due to invalid *P* values with nonnormal conditional distributions. Prediction target: age. Confounder: age. Confound mitigation: age regression. Nonnormality was frequently observed in the other cases, as well. The full analysis is available at https://github.com/pni-lab/mlconfound- manuscript/blob/main/empirical/supplement/check_assumptions.ipynb.

**Supplemental Figure S12**. In case of linearity and normality, the power of the proposed test is virtually equal to that of Pearson’s partial correlation. Blue: partial confounder test; orange: Pearson’s partial correlation. Boxplots are based on the simulation cases from Fig. 4 of the article.

**Supplemental Figure S13**. The partial confounder test can be used at any phase of model validation. The NYU site from the ABIDE dataset has been used as a “discovery sample” to train a model predicting ASD diagnosis. The partial confounder test found no evidence for motion bias. The finalized model has been externally validated in data from the University of Utah School of Medicine (USM). Next to the repeated testing of motion bias, the proposed test is used here for testing another potential confounder (fluid intelligence, *G_f_*) and, additionally, to test if the model generalizes to the SRS (Social Response Scale). Source code available at https://github.com/pni- lab/mlconfound-manuscript/blob/main/empirical/supplement/external_validation.ipynb.

giac082_GIGA-D-22-00097_Original_Submission

giac082_GIGA-D-22-00097_Revision_1

giac082_GIGA-D-22-00097_Revision_2

giac082_Response_to_Reviewer_Comments_Original_Submission

giac082_Response_to_Reviewer_Comments_Revision_1

giac082_Reviewer_1_Report_Original_Submission Bertrand Thirion -- 5/30/2022 Reviewed

giac082_Reviewer_2_Report_Original_Submission Jean-Baptiste Poline -- 7/2/2022 Reviewed

giac082_Supplemental_File

## Abbreviations

ABIDE: Autism Brain Imaging Data Exchange; ASD: autism spectrum disorder; AUC: area under the curve; COMBAT: “combating batch effects” data harmonization approach; CPT: conditional permutation testing; DX: diagnosis; FD: framewise displacement; GAM: generalized additive model; *G_f_*: fluid intelligence; HCP: Human Connectome Project; MCMC: Markov chain Monte Carlo; ROC: receiver operator curve.

## Competing Interests

The authors declare that they have no competing interests.

## Funding

This research was supported by the Deutsche Forschungsgemeinschaft (DFG, German Research Foundation)–Projektnummer 316803389–SFB 1280 and TRR 289 Treatment Expectation—Projektnummer 422744262.
