# Supplementary material for: Statistical quantification of confounding bias in machine learning models
Source: Gigascience. 2022 Aug 26;11:giac082. doi: 10.1093/gigascience/giac082 (PMC9412867; doi:10.1093/gigascience/giac082)
Supplement: giac082_GIGA-D-22-00097_Revision_2 [file giac082_giga-d-22-00097_revision_2.pdf]

GigaScience

Statistical quantification of confounding bias in machine learning models

--Manuscript Draft--

**Manuscript Number:**	GIGA-D-22-00097R2
**Full Title:**	Statistical quantification of confounding bias in machine learning models
**Article Type:**	Research
**Funding Information:**	Deutsche Forschungsgemeinschaft (316803389) — Dr. Tamás Spisák<br>Deutsche Forschungsgemeinschaft (422744262) — Dr. Tamás Spisák
**Abstract:**	Background: The lack of non-parametric statistical tests for confounding bias significantly hampers the development of robust, valid and generalizable predictive models in many fields of research.<br>Here I propose the partial confounder test , which, for a given confounder variable, probes the null hypotheses of the model being unconfounded .<br>Results: The test provides a strict control for Type I errors and high statistical power, even for non-normally and non-linearly dependent predictions, often seen in machine learning. Applying the proposed test on models trained on large-scale functional brain connectivity data (N=1865) (i) reveals previously unreported confounders and (ii) shows that state-of-the-art confound mitigation approaches may fail preventing confounder bias in several cases.<br>Conclusions: The proposed test (implemented in the package mlconfound) can aid the assessment and improvement of the generalizability and validity of predictive models and, thereby, foster the development of clinically useful machine learning biomarkers.
**Corresponding Author:**	Tamás Spisák, Ph.D.<br>Universitatsklinikum Essen<br>Essen, NRW GERMANY
**Corresponding Author Secondary Information:**	
**Corresponding Author's Institution:**	Universitatsklinikum Essen
**Corresponding Author's Secondary Institution:**	
**First Author:**	Tamás Spisák, Ph.D.
**First Author Secondary Information:**	
**Order of Authors:**	Tamás Spisák, Ph.D.
**Order of Authors Secondary Information:**	
**Response to Reviewers:**	Dear Dr. Nogoy,<br><br>Thank you for considering my manuscript "Statistical quantification of confounding bias in machine learning models" (GIGA-D-22-00097R1) for publication in GigaScience.<br><br>This revision includes the following changes:<br>- I have added the GigaDB DOI to the references and cited it under "availability of supporting data" (l. 730).<br>- I have included the RRID (Research Resource Identification Initiative ID) and biotoolsID identifiers in the manuscript (l 455).<br>- I have uploaded the latest latex files into the system<br><br>Please don't hesitate to contact me with any further questions/remarks.<br><br>Best wishes,<br>Tamas Spisak

Powered by Editorial Manager® and ProduXion Manager® from Aries Systems Corporation

Additional Information:	
Question	Response
Are you submitting this manuscript to a special series or article collection?	No
**Experimental design and statistics**<br><br>Full details of the experimental design and statistical methods used should be given in the Methods section, as detailed in our Minimum Standards Reporting Checklist. Information essential to interpreting the data presented should be made available in the figure legends.<br><br>Have you included all the information requested in your manuscript?	Yes
**Resources**<br><br>A description of all resources used, including antibodies, cell lines, animals and software tools, with enough information to allow them to be uniquely identified, should be included in the Methods section. Authors are strongly encouraged to cite Research Resource Identifiers (RRIDs) for antibodies, model organisms and tools, where possible.<br><br>Have you included the information requested as detailed in our Minimum Standards Reporting Checklist?	Yes
**Availability of data and materials**<br><br>All datasets and code on which the conclusions of the paper rely must be either included in your submission or deposited in publicly available repositories (where available and ethically appropriate), referencing such data using a unique identifier in the references and in the "Availability of Data and Materials" section of your manuscript.	Yes

Powered by Editorial Manager® and ProduXion Manager® from Aries Systems Corporation

Have you have met the above requirement as detailed in our Minimum Standards Reporting Checklist?	

LaTeX - Main Document (TeX file)

Click here to access/download;LaTeX - Main Document (TeX file);main.tex

Placeholder for OUP logo oup.pdf

GigaScience, 2022, 1–14

doi: xx.xxxx/xxxx
Manuscript in Preparation
Paper

PAPER

Statistical quantification of confounding bias in machine learning models

Tamas Spisak [1,*]

[1]Center for Translational Neuro- and Behavioral Sciences, Institute for Diagnostic and Interventional Radiology and Neuroradiology, Center , University Hospital Essen

*tamas.spisak@uk-essen.de

Abstract

Background: The lack of non-parametric statistical tests for confounding bias significantly hampers the development of robust, valid and generalizable predictive models in many fields of research. Here I propose the *partial confounder test*, which, for a given confounder variable, probes the null hypotheses of the model being *unconfounded*. **Results:** The test provides a strict control for Type I errors and high statistical power, even for non-normally and non-linearly dependent predictions, often seen in machine learning. Applying the proposed test on models trained on large-scale functional brain connectivity data (N=1865) (i) reveals previously unreported confounders and (ii) shows that state-of-the-art confound mitigation approaches may fail preventing confounder bias in several cases. **Conclusions:** The proposed test (implemented in the package *mlconfound*[1]) can aid the assessment and improvement of the generalizability and validity of predictive models and, thereby, fosters the development of clinically useful machine learning biomarkers.

Key words: machine learning; predictive modelling; confounding bias; confounder test; conditional independence; conditional permutation

Background

Predictive modelling has recently become increasingly important in biomedical research and holds promise for delivering biomarkers that substantially impact clinical practice and public health [1, 2, 3, 4]. When evaluating the usefulness and applicability of such markers, predictive performance is far from being the only important consideration. Biomedical validity and generalizability across contexts and populations are also fundamental requirements for candidate biomarkers [5, 6, 7].

Spurious, out-of-interest associations between the predictor variables (features) and the prediction target can be detrimental to the model's biomedical validity and generalizability. This phenomenon is often called confounding bias [8]. Confounding bias can be driven by various sources. For instance, measurement artifacts, e.g motion artifacts in magnetic resonance imaging-based predictive models, are well known as a potential confounder that can bias the predictive model's output in, among others, Alzheimer's [9], attention deficit hyperactivity disorder [10, 11] or Autism Spectrum Disorder (ASD) [12, 13, 14]). Confounding bias is however not restricted to measurement artifacts. Depending on the research question, several demographic and psychometric variables, or the time of day of the data acquisition [15] can emerge as confounders. As a characteristic example, models trained to predict intelligence [16, 17] might provide a statistically significant predictive performance by picking up solely on age-related variance [18, 19]). Moreover, various types of systematic sampling bias, as well as stochastic group differences in the training sample, can result in confounded models (e.g. racially biased machine learning models [6, 20, 21]).

Confounding-bias is especially problematic in population neuroscience studies. While large-scale multi-site studies are of key importance for developing robust machine learning markers [22], most of the confounding effects are much more likely to occur in such big, longer-term studies [23] and batch and center effects may arise as additional sources of confounding bias [24, 25].

Compiled on: July 27, 2022.
Draft manuscript prepared by the author.

Key Points

- The lack of statistical tests for confounding bias hampers the development of machine learning based biomarker candidates
- The 'partial confounder test' provides a model-agnostic approach for quantifying confounding bias
- It provides strict control for type-I errors and high statistical power with minimal assumptions
- Deploying the test on functional brain connectivity data reveals that confounding bias can be problematic even if confound mitigation approaches are used
- The test provides objective criteria to assess the specificity, generalizability and biomedical validity of biomarker candidates

While various data cleaning methods and dedicated prediction algorithms may help in mitigating confounding bias [9, 26, 13, 27, 28, 29], effects of confounders can potentially bleed through into predictions even if they are being attempted to control for in the prediction algorithm (see Supplementary Analysis 1 for an example) and it is often unclear which variables should be considered as confounders. In a number of cases, removing or controlling for a confounder can remove variance-of-interest and complicate model interpretations [24, 29, 30], rendering the choice of confound mitigation strategy as one of the most difficult compromises in predictive model development.

Powerful and robust statistical tests for quantifying confounding bias in predictive models could substantially foster both the identification of confounders to correct for and the assessment of the effectiveness of various confound-mitigation approaches. It is tempting to think about confounding bias as the *conditional dependence* of the model output on the observed confounder, given the target variable. However, the proper evaluation of conditional independence among these variables is challenging. Namely, even in the presence of a slight non-normality and/or non-linearity of the involved conditional distributions, the 'conditional' analogs of the most popular bivariate non-parametric tests (like the partial Spearman correlation, see Fig. 3) are not valid measures of conditional independence. Although warnings about this issue were given from early on [31], and received a fair amount of attention recently [32, 33, 34, 35, 36], the magnitude of the problem may not be fully appreciated in case of predictive model diagnostics, where non-normality and non-linearity of the model output can be frequently seen (see Supplementary Figures S10-11), as a consequence of e.g. feature-set characteristics and model regularization [37, 38].

Recently, two different approaches were proposed for quantifying confounding bias [39, 40]. However, these methods either fail to control type I error (as known in the case of balanced permutations [41, 42], used in ref. [39]), or do not provide p-values at all [40]. Moreover, without some modifications, they are only applicable for categorical variables and involve re-fitting the model, which may not be feasible for models with high computational cost (e.g. when trained with nested cross-validation).

This work aims to construct a statistical test for confounding bias that (i) guarantees valid type-I error control for arbitrary models, even if non-normal and/or non-linear dependencies are involved (ii) does not require re-fitting the model, (iii) is applicable for classification as well as for prediction problems and both with numerical and categorical confounders.

Methods

Notation and Background

In a predictive modelling setting, let y denote the target variable, $\mathbf{X}$ denote the feature variables, $\hat{y}$ denote model output, i.e. the predictions for y and let c denote a variable which is considered as a confounder. Note, that y and c must be observed during the experiment, whereas $\hat{y}$ is provided by the predictive model. Confounding bias typically emerges in situations where $\mathbf{X} \leftarrow c \rightarrow y$ (arrows denoting dependence of $\mathbf{X}$ and y on c), although $c \rightarrow y$ is not a prerequisite. After fitting the predictive model, we aim to construct predictions based on features unseen during the model training procedure: $\mathbf{X} \rightarrow \hat{y}$ so that $y \rightarrow \hat{y}$. Obviously, a strong association between $\hat{y}$ and c may indicate that the model is biased; its predictions are driven by the confounder rather than information about the target variable. Assessing the simple bivariate (unconditioned) dependence ($H0 : \hat{y} \perp\!\!\!\perp c$) between $\hat{y}$ and c (or any of the $y, \hat{y}, c$ variables) is, however, insufficient for the proper characterization of confounding bias in predictive modelling. For instance, even if $\hat{y} \perp\!\!\!\perp c$ is false, $\hat{y}$ might be only marginally dependent on c, due to the dependence of both on y. In other words, if the target variable y displays a true association to the confounder variable c, a model that is completely blind to c (i.e not confounded at all) might still provide outputs $\hat{y}$ that are significantly associated with c.

Conditional independence for testing confounding bias

Instead of focusing on the 'unconditioned' independence between the confounder and the predictions, we shall consider the *conditional independence* between $\hat{y}$ and c given y (written as $\hat{y} \perp\!\!\!\perp c|y$) which, by definition [43], means that $\mathbb{P}(\hat{y}, c|y) = \mathbb{P}(\hat{y}|y)\mathbb{P}(c|y)$. Testing whether c is independent from $\hat{y}$, conditional on y, is essentially checking whether the path $c \rightarrow \mathbf{X} \rightarrow \hat{y}$ has been blocked in the prediction algorithm. The statistical test with the null hypothesis $H0 : \hat{y} \perp\!\!\!\perp c|y$ will be referred to as the *partial confounder test*. Of note, although typically less useful in a predictive modelling context, one might also be interested in testing $\hat{y} \perp\!\!\!\perp y|c$. We refer to the corresponding test as the *full confounder test*.

Conditional independence - in its general form - is a fundamental concept in statistics with numerous biomedical applications [44, 45, 34, 33]. Recently, [35] have raised important concerns regarding conditional independence testing. Their "no free lunch" theorem implies that, without placing some assumptions on the joint distribution of $(y, \hat{y}, c)$, conditional independence testing is effectively impossible. In other words, neither the full nor the partial confounder tests can be constructed so that - for all distributions - they provide a valid type I error control and, at the same time, a non-trivial statistical power.

This result stands in strong contrast to *unconditional* independence testing - where permutation tests [46, 47], provide a general, distribution-free solution - and it has important implications for confounder testing in predictive modelling where the distribution of the model outputs (conditioned on the target

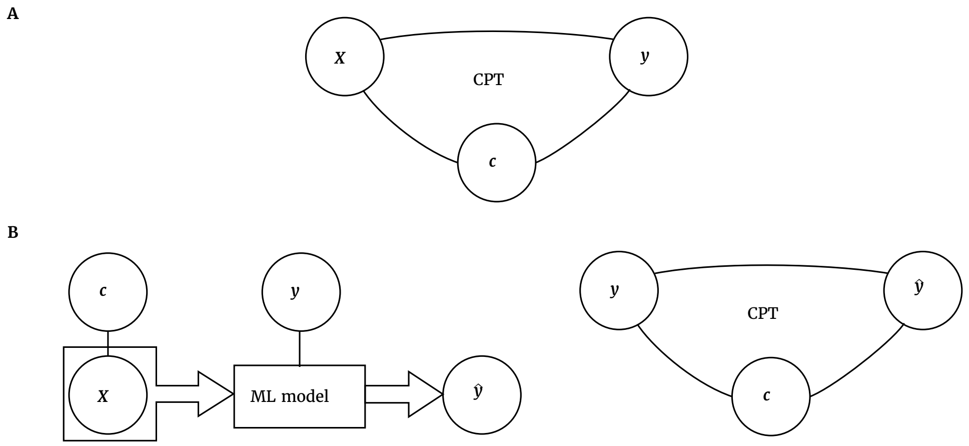

Figure 1. Conditional permutation testing as a tool for predictive model diagnostics.
(A) Conditional Permutation testing (CPT) was originally proposed to be used on the feature variable X, target variable Y and confounders Z, to perform statistical inference. **(B)** The proposed use of CPT in predictive modelling requires the model to be fitted first, to obtain the model's prediction $\hat{y}$ on y. CPT is then utilized on the triplet $(y, \hat{y}, c)$, to test hypotheses $\hat{y} \perp\!\!\!\perp c|y$ or $y \perp\!\!\!\perp \hat{y}|c$. using CPT this way allows lifting assumptions on the prediction target. However, as shown on Fig. 3 the original, can still provide inflated p-values in case of non-linearity in the conditional distributions. False positives can be successfully eliminated by the proposed non-linear techniques for conditional distribution modelling (Fig. 2.)

variable) - depending on the applied machine learning model – is unknown and often non-normal and non-linear. One of the trivial candidates for the task, partial correlation, for instance assumes that all involved variables are multivariate Gaussian and - as to be shown below in a simulated example - even its Spearman-based variant is unable to tolerate relatively small deviations from normality and linearity.

Recently, Candès et al. [33], and, based on their work, Berrett et al. [36], have demonstrated that valid and powerful conditional independence tests can be constructed with inputting distributional information about only two (out of the three) variables. Specifically, the conditional permutation test (CPT) of Berrett and colleagues samples from a non-uniform distribution over the set of possible permutations π of one of the variables, based on its conditional distribution of the other variable. Thereby, it incorporates the information available about the conditional distribution of interest into the permutation-based inference in a statistically valid manner.

Like many related papers, the work of Berrett et al. was formalized as a (semi-)supervised learning approach, where X is a set of predictors (features), y is the target variable and c is a potential confounder (Figure 1A). In this setting, testing the null hypothesis $X \perp\!\!\!\perp y|c$ aims to determine, whether the features X still affect y, when controlling for c. For instance, in genome-wide association studies, CPT can be used to determine whether a particular genetic variant X affects a response y such as disease status or some other phenotype, even after controlling for the rest of the genome, encoded in c.

In this paper, a different setting is considered, where the supervised learning model is already fitted (Figure 1B) and we are focusing on model diagnostics, by testing the triplet $(y, \hat{y}, c)$, with the requirement of minimal assumptions on the conditional distribution of $\hat{y}$ on y and c (Figure 1C).

Within this setting, conditional independence testing and, specifically, the framework of conditional permutation testing allows investigating three different null hypotheses corresponding to the $(y, \hat{y}, c)$ triplet. As listed in Table 1, testing the null hypothesis $y \perp\!\!\!\perp \hat{y}|c$ (option 1, full confounder testing) investigates whether the predictions are likely explainable solely with the confounder, i.e. whether the model is exclusively confounder-driven. Testing $y \perp\!\!\!\perp c|\hat{y}$ (option 2) addresses the question whether the model captures all the variance in c when predicting y. Testing the null hypothesis $\hat{y} \perp\!\!\!\perp c|y$ (option 3, partial confounder testing) examines, whether the dependence of the model output on the confounder can likely be explained by the confounder's dependence on the target variable, i.e. whether there is any confounding bias in the model.

Option 3, i.e. partial confounder testing is typically of interest when testing confounding bias of predictive models. Option 1, i.e. full confounder testing may be less useful in practice, although it might provide valuable insights in the exploratory phase of model construction. Option 2 does not seem appealing for model diagnostics and importantly, in this case the proposed variety of the CPT framework does not allow constructing a test which is non-parametric on $\hat{y}$. We will therefore focus on option 3, i.e. the partial confounder test.

In the following section, CPT is adapted for *partial* confounder testing and extended with general additive model [48] (GAM) and multinomial logistic regression [49, 50] based conditional distribution estimations, in order to make it handle categorical data and non-linear dependencies between the confounder and the target variable. (For an overview of the method, see Fig. 2).

The partial confounder test

The inner workings of the *partial confounder test* are summarized on Fig. 2. In short, the test models the conditional distribution between the confounder and the target variable with a GAM - or with an *mnlogit* regression, in case of categorical confounder - and then uses a so-called parallel-pairwise Markov-chain Monte-Carlo sampler of [36] that draws permutations of the original confounder, so that the permuted variables still comply with the estimated conditional distribution. As a result, the permuted "copies" of the confounder variable retain its correlation with the target variable but eliminate any "additional" relationship with the model output. The test statistic (coefficient of determination, R^2) is then computed between the model output and the original, as well as the permuted confoudner variables. The original and the permuted test statistics construct the p-value as the ratio of permuted test statistics more extreme than the original.

In detail, the partial confounder test generates a null-distribution for an arbitrary predefined test statistic $T(y, \hat{y}, c)$ by sampling permutation based 'copies' of the original c,

$$c_i^{(j)} \sim Q(\cdot|y_i) \tag{1}$$

	H0		assumption needed for:	no assumptions about the distribution of:
**1.**	$\hat{y} \perp\!\!\!\perp y \vert c$	full confounder test: model exclusively driven by the confounder	$Q(y\vert c)$	$(\hat{y}, y), (\hat{y}, c)$
**2.**	$y \perp\!\!\!\perp c \vert \hat{y}$	model captures all variance in the confounder (not of interest)	$Q(c\vert\hat{y})$	$(y, c), (y, \hat{y})$
**3.**	$\hat{y} \perp\!\!\!\perp c \vert y$	partial confounder test: model not directly driven by the confounder	$Q(c\vert y)$	$(\hat{y}, c), (\hat{y}, y)$

Table 1. Possibilities when testing conditional independence in potentially biased predictive models.
The table lists the three possible null hypotheses (H0), and the variables where assumption about the joint/conditional distributions is required/not required. (y: prediction target, $\hat{y}$: predictions, c: confounder variable)

where, $Q(.|y)$ denotes the conditional distribution of c given $y = y_i$ and $j = 1, \ldots, m$ indexes the 'copy' of c so that

$$c^{(j)} = (c_1^{(j)}, \ldots, c_n^{(j)}) = (c_{\pi_1^{(j)}}, \ldots, c_{\pi_n^{(j)}}) = c_{\pi^{(j)}}$$

is a permutation of the original vector $c = (c_1, \ldots, c_n)$, with its elements reordered according to the permutation $\pi \in S_n$ where S_n denote the set of all permutations on the indices $\{1, \ldots, n\}$.

As shown by [36], to ensure that Eq. 1 holds, the $c_{\pi^{(j)}}$ copies must be drawn so that:

$$\mathbb{P}(\pi^{(j)} = \pi | y, \hat{y}, c) = \frac{q^n(c_\pi | y)}{\sum_{\pi' \in S_n} q^n(c_{\pi'} | y)} \qquad (2)$$

that is, according to the $q^n(\cdot|y) := q(\cdot|y_1) \ldots q(\cdot|y_n)$ product density corresponding to the conditional distribution $Q(\cdot|y)$. Note that Eq. 2 does not necessarily assume a continuous distribution.

This mechanism creates copies $c^{(1)}, \ldots, c^{(m)}$ so that under the null hypothesis ($\hat{y} \perp\!\!\!\perp c|y$), the triples

$$(y, \hat{y}, c), (y, \hat{y}, c^{(1)}), \ldots, (y, \hat{y}, c^{(m)})$$

are all identically distributed and so are the

$$T(y, \hat{y}, c), T(y, \hat{y}, c^{(1)}), \ldots, T(y, \hat{y}, c^{(m)})$$

test statistics, as well.

As long as the numerator of Eq. 2 is non-zero for all $c_\pi \in C$ and $y \in Y$, the conditional permutations constitute an algebraic group, thus, as shown by Hemerik and Goeman [42], an unbiased estimate of the p-value under the null can be obtained as:

$$p = \frac{\sum_{j=1}^{m} \mathbb{1}\{T(y, \hat{y}, c^{(j)}) \geq T(y, \hat{y}, c)\}}{m}$$

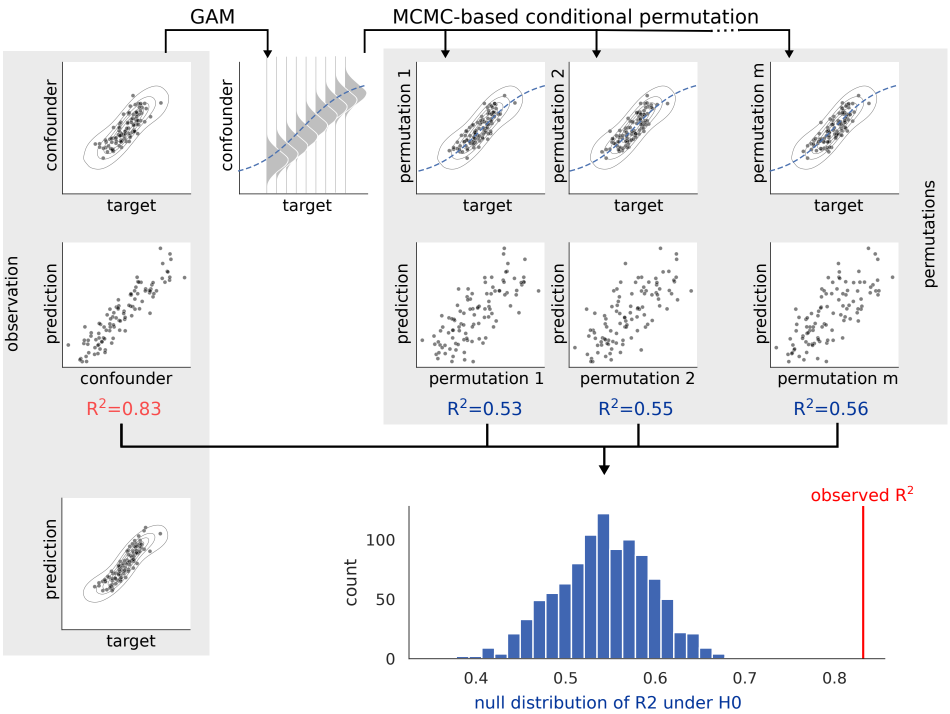

Figure 2. Graphical representation of the proposed partial confounder test.
The partial confounder test models the conditional distribution of the confounder, given the target variable, with a generalized additive model (GAM). The parallel-pairwise Markov-chain Monte-Carlo (MCMC) sampler draws permutations of the original confounder variable that comply with the GAM-based conditional distribution (permutation 1, 2, ..., m). The test statistic (coefficient of determination, R^2) is then computed between the model output and the original, as well as the permuted confounder variables. The original and the permuted test statistics construct the p-value as the ratio of permuted test statistics more extreme than the original. Figure source code available as jupyter notebook: https://github.com/pni-lab/mlconfound-manuscript/blob/main/simulated/overview-fig.ipynb

While the group property of the conditioned permutations provides a straightforward proof for the validity of the approach, for an alternative verification see the proof of Theorem 1 in [36].

The required permutations could be theoretically sampled with a simple Metropolis-Hastings algorithm that draws uniformly from S_n at random. However, this way the acceptance ratio would be extremely low, even for moderate n (except there is very low dependence of $\boldsymbol{c}$ on y), resulting in slow mixing times. The partial confounder test can be, however, efficiently implemented with the parallelized pairwise Markov-Chain Monte Carlo sampler of [36] (Algorithm 1), that draws disjoint pairs in parallel and decides whether or not to swap them randomly, according to the odds ratio calculated from the conditional densities belonging to the original and swapped data. The acceptance odds ratio of swapping indices i and j is:

$$\ln \frac{q(c_j|y_i)q(c_i|y_j)}{q(c_i|y_i)q(c_j|y_j)} = \ell(c_j|y_i) + \ell(c_i|y_j) - \ell(c_i|y_i) - \ell(c_j|y_j) \quad (3)$$

where ℓ denotes the log-likelihood.

In their Theorem 2, [36] verify that the resulting Markov Chain yields the desired stationary distribution, even if the number of steps is small.

Conditional log-likelihood

Obtaining a relatively accurate, independent estimate of $Q(\cdot|y)$ (of any shape) for CPT inference is important. Berrett and colleagues recommend to use a large independent sample to obtain the log-likelihood matrix that represents the conditional distribution $Q(\cdot|Z)$ or, alternatively, to re-use the data by fitting a least squares linear regression:

$$\boldsymbol{c} = \alpha + \beta \boldsymbol{y} + \boldsymbol{e} \quad (4)$$

As the linear regression-based method, obviously, does not handle non-linear relationships, I propose to apply a modelling approach that accounts for non-linearity. Although several nonparametric techniques might be suitable for this purpose, many of these tend to be greedy for large sample sizes, may lack stability or perform poorly with many potential predictors. Certain methods, such as kernel methods and smoothing splines, are also very difficult to interpret [51]; an important consideration when analyzing the source of a confounder effect.

Here, I propose to use the generalized additive model (GAM) of [48]:

$$\boldsymbol{c} = \alpha + \beta f(y) + \boldsymbol{e} \quad (5)$$

where the feature functions f is built using penalized B-splines, which allow us to automatically model non-linear relationships without having to manually try out many different transformations on each variable. The principal advantages of GAM are that (i) the complexity of the model can be effectively regularized trough its hyperparameters, (ii) it is able to model highly complex nonlinear relationships with a potentially large number of both numeric and categorical predictors and (iii) it has computationally effective solver algorithms. The potential disadvantages of GAMs are not relevant for the problem at hand or can be easily overcame. Specifically, the possibly poor out-of-distribution generalization of GAM is not problematic, as in our approach the model is not used for constructing out-of-distribution predictions. Moreover, as several other models, GAMs can easily overfit the data. However, in the proposed approach, the smoothness of the GAM model is optimized with a grid-search by picking the model with the lowest generalized cross-validation score from the models defined by the default parameters as implemented in PyGAM [52] (v0.8.0).

If we write $\mu = \alpha + \beta f(y)$ and σ denotes the standard deviation of the residual $\boldsymbol{e}$, then the conditional distribution of interest can be assumed to be normal with the parameters:

$$(\boldsymbol{c}|\boldsymbol{y} = y_i) \sim \mathcal{N}\{\mu_i, \sigma^2\}$$

and the log-likelihood, that is to be used in Eq. 3, can be computed simply as the log of the corresponding probability density function:

$$\ell(c_i|y_j) = -\frac{1}{2}\left(\frac{c_i - \mu_j}{\sigma}\right)^2 - ln(2\pi\sigma)$$

In the case of categorical $\boldsymbol{c}$, a multinomial logistic regression (*mnlogit*) model can be used to obtain $D(\cdot|y)$, with the extra assumption of *complete separation* if $\boldsymbol{y}$ is also categorical (in order to ensure an invertible Hessian, see e.g. [49, 50]).

Importantly, both the GAM- and the *mnlogit*-based approaches guarantee that the numerator of Eq. 2 is always greater than zero and the group property for the permutations holds.

Note that from the three options for conditional independence-based null hypotheses enumerated in Table 1, the proposed approach can not provide a test for option 2 that is assumption-free about $\hat{y}$, as the variable, on which the independence is conditional, must be always the predictor variable in Eq. 5. However, as discussed above, this option is of low practical relevance, anyway. Pleasingly, the proposed Gaussian regression-based conditional likelihood estimation ensures that no assumptions on $\hat{y}$ have to be made for the practically relevant options 1 and 3, i.e. for the full and partial confounder tests.

In theory, any predefined test statistic T can be used with the proposed approach. The python package *mlconfound*, implementing the proposed full and partial confounder tests, utilizes the coefficient of determination (R^2 or pseudo R^2 in case of categorical confounder or classification [53]) as a test statistic: $T(y, \hat{y}, c) = R^2(\hat{y}, c)$ and $T(y, \hat{y}, c^{(j)}) = R^2(\hat{y}, c^{(j)})$ which allows interpretable, two tailed inference.

Validation on simulated data

Using CPT to test confounding bias in predictive modelling allows relaxing assumptions on $\hat{y}$ but - in line with the "no free lunch" theorem, requires knowing - or putting assumptions on - the joint distribution of the other two variables (y and c). [36] give a detailed analysis of the robustness of their CPT approach when estimating the conditional distribution with re-using the tested data via linear regression and, also, against misspecifying the conditional distribution of interest to introduce non-linearity.

Here I extend these results by performing simulations that evaluate the GAM- and *mnlogit*-based approaches, in a form that is accessible for power calculations in predictive modelling (considering various weights of the target signal in $\boldsymbol{c}$ and the confounder and the target signals in $\hat{y}$). Moreover, I investigate the robustness of the tests against the violation of normality and linearity of the conditional distributions $D(\boldsymbol{c}|\boldsymbol{y})$ and $D(\hat{\boldsymbol{y}}|\boldsymbol{y})$.

Simulations are performed separately for the two proposed tests.

Simulation approach

As a first step, the target variable y is drawn randomly from a normal distribution:

$$y \sim \mathcal{N}(0,1)$$

Next, the confounder signal is simulated as:

$$c|y_i \sim f_{\delta,\epsilon}(\mathcal{N}(0,1)) + w_{yc}\, g(y_i)$$

where f is a function to introduce non-normality, namely the *sinh-arcsinh* transformation of [54], defined as:

$$f_{\delta,\epsilon}(x) = sinh(\delta sinh^{-1}(x) - \epsilon)$$

where the parameters δ and ϵ control the kurtosis and skewness of the resulting *sinh-arcsinh* distribution, with $\delta = 1$ and $\epsilon = 0$ producing the identity function (i.e. no non-normality introduced).

Moreover, non-linearity can be introduced with the function g, which can be simply the identity function (no non-linearity is introduced in this case) or, for instance, a sigmoid-shaped function, in our case:

$$g(x) = tanh(x)$$

The simulated predicted values are constructed in a similar fashion, but may depend on c as well:

$$\hat{y}|y_i, c_i \sim f_{\delta,\epsilon}(\mathcal{N}(0,1)) + w_{y\hat{y}}\, g(y_i) + w_{c\hat{y}}\, c_i$$

Note that simulations with $w_{c\hat{y}} = 0$ produce data under the null hypothesis of no confounding bias.

To test the implementation for categorical variables, simulated y, $\hat{y}$ and c variables are binarized by thresholding at 0.

Simulations for comparison with partial Spearman correlation and linear CPT

To demonstrate the need for the proposed GAM-based CPT approach for partial confounder testing (Fig. 3), its validity was contrasted to partial Spearman correlation and the linear variety of CPT (based on eq. 4, as described by [36]) with the following simulation parameters: sample size $n = 1000$, $w_{c\hat{y}} = 0$ (i.e. H0 simulations only), taking all combinations of $w_{yc} \in \{0.5, 1, 2, 3\}$ and $w_{y\hat{y}} \in \{0.5, 1, 2, 3\}$. Furthermore, simulations cases with non-normality ($f_{\delta=0.1,\epsilon=2}$) and non-linearity (sigmoid g) has also been investigated for all simulation cases.

For each parameter combination, 1000 repetitions were performed and false positive rates were calculated as the ratio of p-values smaller than $\alpha = 0.05$.

The simulation cases are exemplified (with $w_{yc} = w_{y\hat{y}} = 2$) on the left of Figure 3.

Simulations for evaluating power.

100 repetitions were performed of all combination of the following parameter values: $w_{yc} \in \{0.5, 1, 2, 3\}$, $w_{y\hat{y}} \in \{0.5, 1, 2, 3\}$, $w_{c\hat{y}} \in \{0, 0.2, 0.4, 0.6\}$, $n \in \{50, 100, 500, 1000\}$. All simulations were performed with both linear and sigmoid dependence as well as with normal and non-normal conditional distributions: $(\delta, \epsilon) = \{(0.1, 2), (1, 0), (1.05, -3), (1.5, -5), (5, -10)\}$.

The partial confounder tests, as implemented in version 0.20.0 of the package *'mlconfound'* was run with default parameters (1000 permutations and 50 Markov-chain Monte-Carlo steps to generate the conditioned permutations) and by implying categorical variables, where needed.

All code used for the simulations is available on github[2].

Application on functional brain connectivity data

The usefulness of the proposed confounder tests is demonstrated by applying them for predictive classification and regression models based on functional brain connectivity data, processed with different confound-mitigation approaches.

Partial confounder testing was performed with 10000 permutations and 50 Markov-chain Monte Carlo steps, as implemented in version 0.20.0 of the package *'mlconfound'*. Unconditional dependence across the involved variables was investigated with conventional permutation tests on the R^2 values, with 1000 permutations.

All empirical analyses are available as jupyter notebooks on github[3].

HCP: testing age and acquisition batch bias in fluid intelligence prediction

The Human Connectome Project dataset contains imaging and behavioral data of approximately 1200 healthy subjects [55]. Preprocessed resting state fMRI connectivity data (partial correlation matrices) [56] as published with the HCP1200 release (N=999 participants with functional connectivity data) were used to build models that predict individual fluid intelligence scores (G_f), measured with Penn Progressive Matrices [57].

To ensure normality of the target variable for the partial correlation-based analyses, G_f was non-linearly transformed to normal distribution with the quantile transformation [58] as implemented in *scikit-learn* [59] (see Supplementary Figure S8 for details).

Features (functional connectivities across 100 group-independent component analysis based regions) were either (i) considered in their raw form or were subject to confound mitigation approaches by (ii) feature regression [9] or (iii) COMBAT [28, 60]. The feature mitigation strategies were separately applied for acquisition batch and age group as confounder variable.

Each of the 5 types of features (raw, regressing out acquisition batch, regressing out age group, COMBAT with acquisition batch, COMBAT with age group) was independently incorporated into a scikit-learn-based [59] machine learning procedure aiming to predict the individual fluid intelligence scores with a ridge regression [61]. The α parameter of the ridge model was considered as a hyperparameter ($\alpha \in \{0.00001, 0.0001, 0.001, 0.01, 0.1, 1, 10, 100, 1000, 10000, 100000\}$) and optimized in a nested cross-validation with 10 folds both in the inner and the outer loop and with mean squared error as optimization metric. Confound mitigation was performed inside of the outer cross-validation loop, to avoid leakage.

ABIDE: testing motion- and center-bias in predictive models of autism spectrum disorder diagnosis

The proposed tests were applied to provide evidence of center- and motion-bias in diagnostic predictive models of autism spectrum disorder (ASD), trained on the Autism Brain Imaging Data Exchange (ABIDE) dataset [62] involving 866 participants (ASD: 402, neurotypical control: 464). Preprocessed regional timeseries data was obtained as shared [4] with the by Dadi et al. [63] which was based on preprocessed image data provided by

2 https://github.com/pni-lab/mlconfound-manuscript/tree/main/simulated
3 https://github.com/pni-lab/mlconfound-manuscript/tree/main/empirical
4 https://osf.io/hc4md

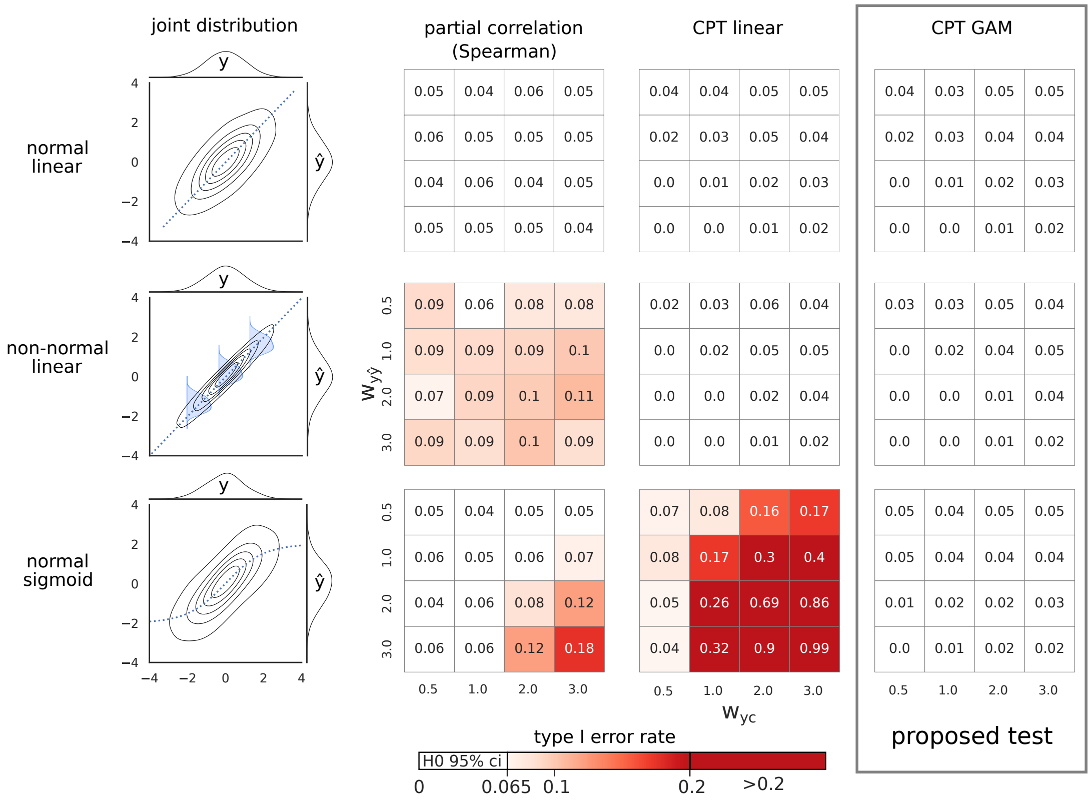

Figure 3. Type I error control of partial Spearman correlation, linear and GAM-based conditional permutation test.
Type I error control was investigated in three example cases: normal conditional distribution with linear dependency (first row), slightly non-normal conditional distribution with linear dependency (second row) and normal conditional distribution with non-normal (sigmoid) dependency (third row). Non-normal conditional distribution on the second plot is illustrated with blue density diagrams (kurtosis: -0.8, skewness: -0.1). False positive rates for confounder contributions (w_{yc}, ranging from 0.5 to 3.0) and predictive performances ($w_{y\hat{y}}$, ranging from 0.5 to 3.0) is shown in heatmaps. The upper limit for the binomial confidence interval corresponding to *alpha* = 0.05 is 0.065. Values below this threshold (colored white) indicate a valid type I error control.

the Preprocessed Connectome Project [64].

Tangent correlation across the timeseries of the n=122 regions of the BASC [65] brain atlas was computed with nilearn[5] [66, 67].

The resulting functional connectivity estimates were considered as features either (i) in their raw form or after applying (ii) feature regression [9] or (iii) COMBAT [28, 60]. The investigated confounder variables were 'imaging center' and 'in-scanner motion', as measured by the mean framewise displacement (FD), as defined by [68]. Mean FD was non-linearly transformed to normal distribution with the quantile transformation [58] as implemented in *scikit-learn* [59] (see Supplementary Figure S9 for details).

As COMBAT is not able to handle continuous variables (since it was primarily designed to remove categorical "batch-effects"), motion was binned into 10 groups, based on equidistant data quantiles ranging from 0 to 1.

The total of five types (raw, feature regression of site, feature regression of motion, COMBAT with site, COMBAT with motion) of features were independently incorporated into a scikit-learn-based [59] machine learning procedure aiming to predict the diagnosis (DX: ASD vs. neurotypical controls) with a L2-regularized logistic regression, as previously recommended [63]. The C parameter of the model was considered as a hyper-parameter ($C \in \{0.1, 1, 10\}$) and optimized in a nested cross-validation with 10 folds both in the inner and the outer cv-s and with area under the receiver operator curve (AUC under ROC) as optimization metric. Confound mitigation was performed inside of the outer cross-validation loop, to avoid leakage. Confounder testing was performed on the predicted class probabilities.

Results

The partial confounder tests

The proposed *partial confounder tests* has been implemented in the python package *mlconfound*[6] (biotools:mlconfound, RRID:SCR_022545).

Simulations

Type I error

As suggested by theory (see Methods for details) and shown by the simulations with a wide range of settings, both of the proposed tests provide a valid Type I error control (Fig. 4 and Supplementary Figures S1-3), even in case of non-linearity and non-normality (Figs. 3, 5 and Supplementary Figures S4-7), except when non-normality is extreme (purple distribution on Fig. 5, kurtosis: 42, skewness: -6).

Power

Estimates of statistical power for the partial confounder test (with normal and linear simulations, for a wide range of pa-

5 http://nilearn.github.io/

6 https://mlconfound.readthedocs.io

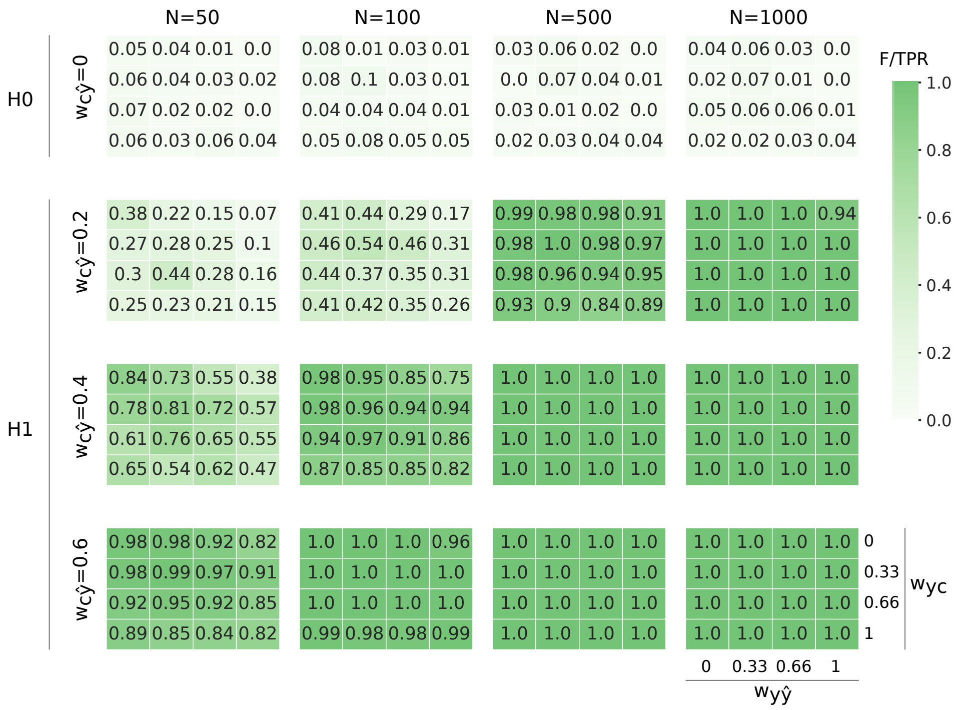

Figure 4. The partial confounder test provides a strict control for Type I errors and a high statistical power in simulated data.
Heatmaps depict positive rates (ratio of p-values lower than 0.05, color coded as shown by the palette on the right) in various simulations settings (100 simulations per tile) with different simulation weights $w_{y\hat{y}}$ (predictive performance; horizontal axis on each heatmap), w_{yc} (confounder-target association; vertical axis on each heatmap), $w_{c\hat{y}}$ (degree of confounder bias; rows) and for different sample sizes (N, columns). Weights 0.2, 0.33, 0.4, 0.6, 0.66, 1.0 can be assigned to the following approximate explained variance values: 4%, 10%, 12%, 25%, 30%, 50%, respectively. First row contains simulations under the null hypothesis (H0, no confounding bias), rows 2-4 represent simulations from the alternative hypothesis (H1, confounding bias). Positive rates for the simulations under the null and the alternative hypotheses can be interpreted as type I error rate and statistical power, respectively. The higher 95% confidence limit for a positive rate of *alpha* = 0.05 is 0.11 for each tile.

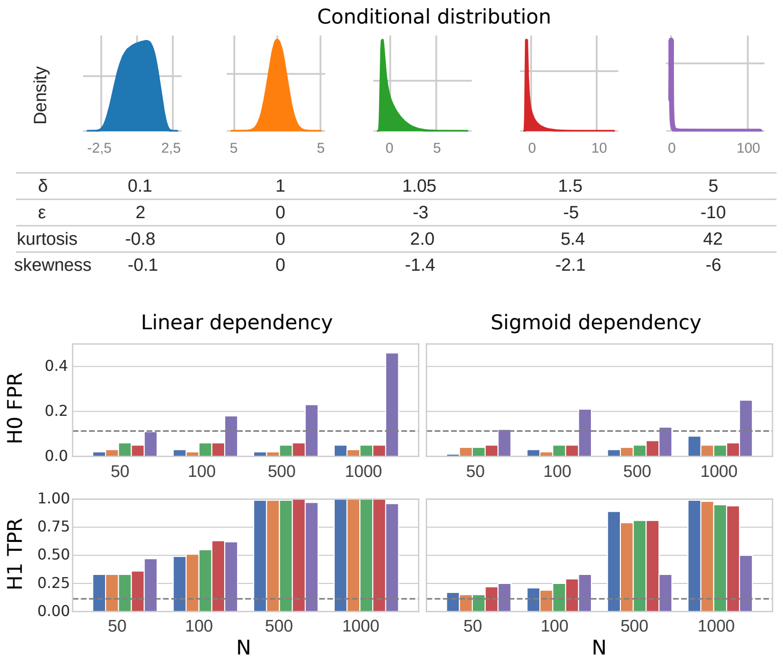

Figure 5. The partial confoudner test is robust to non-normality and non-linearity.
Simulations included variables with five different degrees of non-normality (top panel), as introduced with various δ and ϵ values of the *sinh-arcsinh* transformation (yellow: normally distributed). Fisher's kurtosis and skewness is given for each distribution. False and true positive rates in the simulations under H0 and H1, respectively, for each investigated sample size (N), are depicted by barplots for both linear and sigmoid dependency structure. Upper 95% binomial confidence limit corresponding to *alpha* = 0.05 is shown with a vertical dashed line.

rameters) were found to be virtually identical to those of Pearson's partial correlation. (see Figure 4 and Supplementary Figure S12). Notably, with sample sizes as large as 1000, a confounder contributing only ∼ 4% to the variance of the predic-

tions ($w_{c\hat{y}}$ = 0.2) can already be robustly detected with a power of 94-100%. With a sample size of 500, the same confounding bias is still detected with a power greater than 84-100% in all of the simulation cases. A sample size of 100 requires a somewhat stronger bias with approximately 12% of explained variance ($w_{c\hat{y}}$ = 0.4) to achieve a reasonable level of power (75-98%). Finally, even with a relatively low sample size of 50, the same amount of confounder variance is detected with a power of at least 50%. If the confounder explains more than 25% of variance, it is almost certainly detected even with a low sample size of $n \geq 50$.

Simulations show that non-normality has minimal effect on the power of the tests, except in case of extreme non-normality. (Fig. 5). Simulations with sigmoid dependence resulted in an apparent loss of statistical power, however this is simply a consequence of the simulation methodology: with the same parameters, the sigmoid transformed confounder explains only approximately half the variance as compared to linear simulations. Type I error control was valid in case of categorical variables, as well (Supplementary Figures 1,3,5,7).

Neuroimaging data

To demonstrate the usefulness of the proposed tests in detecting various types of confounding bias, they have been deployed in two typical research scenarios - a regression and a classification problem - where confounder effects are known to hamper the development of biomedically useful predictive models. The empirical analyses confirmed the presence of non-linearity and non-normality in the output of the predictive models (see Supplementary Figure S11 for more details).

HCP dataset

Functional connectivity data from the Human Connectome Project [55] (HCP) was used to build predictive models of fluid intelligence (G_f) and to test for the previously discussed confounding effect of age [19, 18] and, additionally, the - somewhat underdiscussed - batch-like effect of acquisition date of the data within the course of the data acquisition process.

Both acquisition batch and age group were statistically significantly associated with G_f (R^2 = 0.032 and 0.011 and $p <$ 0.001 and p = 0.001, respectively, see also Table 2). The model trained on the raw (unadjusted) connectivity features predicted fluid intelligence with a medium effect size (R^2 = 0.095, $p <$ 0.001).

The partial confounder test revealed that the 'raw' model (without confounder mitigation) was significantly biased both by age group and acquisition batch (both $p < 0.0001$, first column of Fig. 6) with later phases of the acquisition and lower age being associated to larger predicted values.

After applying confound mitigation approaches (feature regression or COMBAT) the partial confounder test did not provide evidence for confounding bias anymore ($p > 0.05$ for all; shown in the second and third columns of Fig. 6), neither for acquisition batch nor for age. Both feature regression and COMBAT increased the predictive performance, with COMBAT providing the overall best performances (R^2 = 0.122 and 0.121 when applied to remove the effect of acquisition and age, respectively).

ABIDE dataset

Functional connectivity data from the ABIDE [62] database was used to investigate the potential motion and center bias (as previously reported e.g. by [13, 14] or [12]) when training models that aim to predict ASD diagnosis.

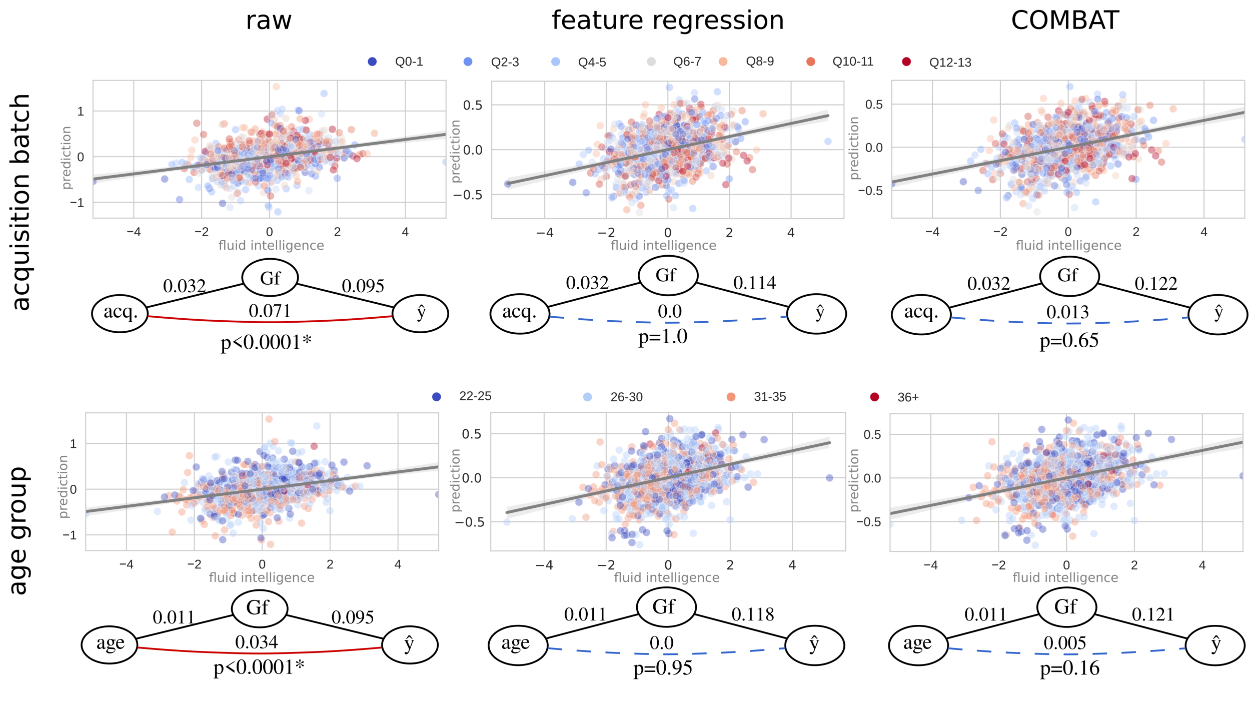

Figure 6. The partial confoudner test reveals that acquisition batch- and age-bias in predictive models of fluid intelligence can be effectively attenuated by confounder mitigation approaches in the HCP dataset.
Scatter plots and regression lines (with 95% confidence intervals) show the association of the observed (horizontals axis) and predicted (vertical axis) fluid intelligence scores with various confound regression strategies. Color-coding of the confounder variables (top: acquisition batch, bottom: age group, as shown by the corresponding legends) reveals confounding bias both for acquisition and age in the models trained on the raw data. This bias is robustly detected by the partial confounder test ($p < 0.0001$) and seems to be effectively mitigated by both feature regression and COMBAT. Relation between the observed (Gf) and predicted ($\hat{y}$) intelligence scores and the confounder variables is given on the graphs via R^2*values*. Both confound mitigation techniques, but especially COMBAT, improve the predictive performance. Solid red line between the confounder and the prediction means significant confounding bias, whereas blue dashed line denotes that confounder testing provided no evidence for bias. P-values are determined with the partial confounder test.

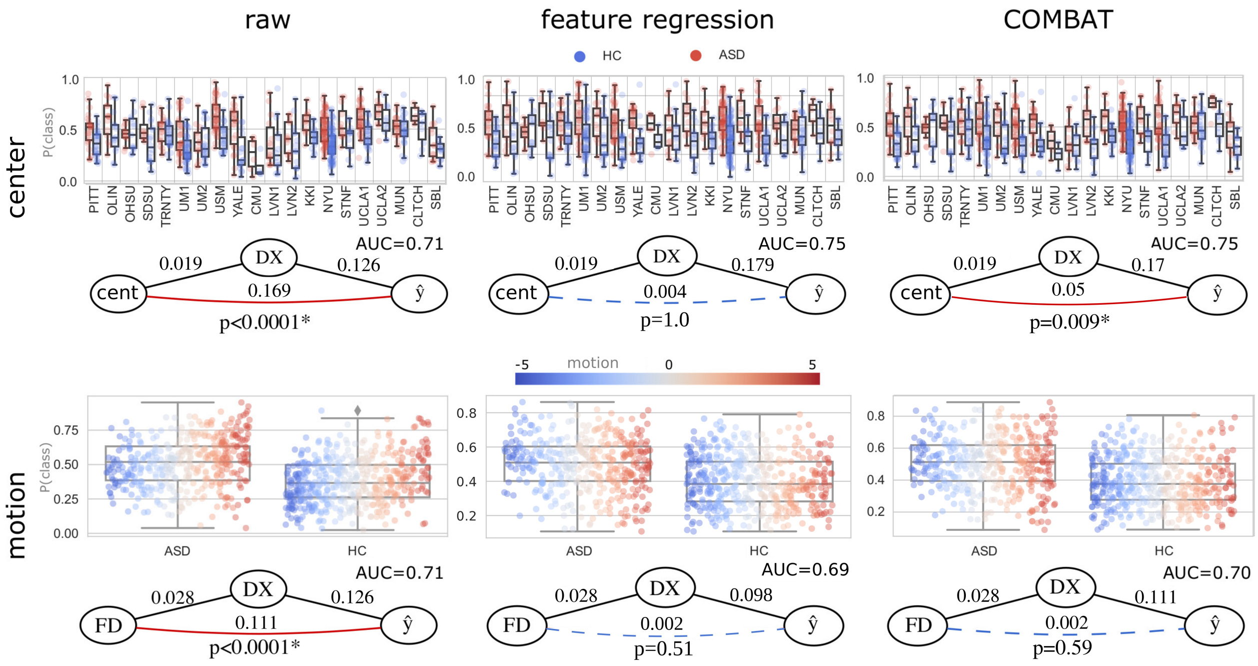

Figure 7. The partial confoudner test identifies an efficient mitigation strategy for motion-bias in predictive models of autism spectrum disorder and reveals residual center-bias after COMBAT in the ABIDE dataset.
Boxplots and points show the predicted class probabilities (0: HC, 1: ASD), separately for the HC and ASD groups. In the top panel, predictions are plotted for each center separately. Color indicates the true diagnosis (DX). AT the bottom plot, color indicates the normalized index of in-scanner motion (normalized FD). The proposed confounder test reveals significant center and motion bias in the model trained on the raw data (p<0.0001). While both motion and center bias was effectively mitigated by both feature regression and COMBAT, the proposed partial confounder test revealed COMBAT was not able to fully remove center bias and resulted in significant "residual bias" (p<0.05). Relation between the true ($\hat{y}$) and predicted diagnosis scores and the confounder variables is shown by the graphs as R^2 values. Solid red line between the confounder and the prediction means significant confounding bias, whereas blue dashed line denotes that confounder testing provided no evidence for bias. P-values are determined with the partial confounder test.

Imaging center and in-scanner motion (normalized mean framewise displacement) were statistically significantly associated with ASD diagnosis (R^2 = 0.019 and 0.028, respectively, $p < 0.001$ for both, see also Table 2). The model trained on the raw (unadjusted) connectivity features predicted diagnosis with a medium effect size (R^2 = 0.126, $ROCAUC$ = 0.71, $p < 0.001$).

The partial confounder test revealed that the raw model was significantly biased both for age group and acquisition batch (both $p < 0.0001$, see first column on Fig. 7). Predictions for several sites (e.g. Carnegine Mellon University, University of Leuven, Social Brain Lab UMC Groningen) were severely miscalibrated and higher motion was associated to a higher probability for ASD diagnosis.

Both feature regression and COMBAT seemed to significantly attenuate center bias, however, with COMBAT, the partial confounder test still provided evidence for a significant residual bias (0.009, third columns of the first row on Fig 7).

When trying to mitigate the effect of in-scanner motion (bottom row on Fig 7), both confounder mitigation approaches seemed to effectively mitigate motion-bias, as suggested by the partial confounder test ($p > 0.05$, middle and right panel in the bottom row of Fig 7).

Both feature regression and COMBAT considerably improved the predictive performance when mitigating center-effects (AUC = 0.71 without correction and 0.75 with both feature regression and combat). With both feature regression and COMBAT, however, the effort of mitigating motion effects happened at the cost of a drop in predictive performance (AUC = 0.69 and 0.70, for feature regression and COMBAT, respectively)

Discussion

The concept of conditional independence provides a straightforward framework for assessing confounding bias in predictive models , assuming that both the target variable and the potential confounder has been observed for the validation dataset. However, handling the non-normal and/or non-linear conditional dependencies often seen in predictive models [37, 38] (Supplementary Figures S10-11) poses a great challenge. In fact, as recently shown by Shah and Peters in their 'no free lunch' theorem [35], it is effectively impossible to establish a *fully non-parametric* conditional independence test with a valid type I error control and a non-trivial power. Indeed, perhaps somewhat surprisingly, but not totally unexpectedly [31] – partial correlation-like analogs of widely used bivariate non-parametric test, like partial Spearman correlation, exhibit inflated type I errors even with slight violations of normality and/or linearity (as clearly demonstrated with simulated data on Fig. 3). While the magnitude of this problem may not be fully appreciated in case of predictive model diagnostics, such tests are, in general, poor choices for testing confounding bias in machine learning. Conditional independence-based confounding bias testing must, therefore, be designed so that its suitability for the particular problem may be judged easily.

These tests place no assumptions on the conditional distributions of the model output, ensuring valid model diagnostics even in cases of non-normally and non-linearly dependent predictions. This property distinguishes the approach from other alternatives as it guarantees a valid type-I error control even in cases of non-normally and non-linearly dependent predictions, i.e. in cases where Pearson and Spearman partial correlations, and many other methods fail.

The proposed tests are based on solid theoretical foundations, underpinned by mathematical proofs. The main purpose

dataset	conf.	method	$R^2_{y,c}$	$p_{y,c}$	$R^2_{\hat{y},c}$	$p_{\hat{y},c}$	$R^2_{\hat{y},y}$	$p_{\hat{y},y}$	partial confounder test
HCP	acq.	raw	0.032	<0.001	0.071	<0.001	0.095	<0.001	**<0.0001**
		f.reg.			0.0	1.0	0.114	<0.001	1.0
		COMBAT			0.013	0.4	0.122	<0.001	0.65
	age	raw	0.011	0.001	0.034	<0.001	0.095	<0.001	**<0.0001**
		f.reg.			0.0	0.92	0.118	<0.001	0.95
		COMBAT			0.005	0.048	0.121	<0.001	0.16
ABIDE	center	raw	0.019	<0.001	0.169	<0.001	0.126	<0.001	**<0.0001**
		f.reg.			0.004	1.0	0.179	<0.001	1.0
		COMBAT			0.05	0.001	0.17	<0.001	**0.009**
	motion	raw	0.028	<0.001	0.111	<0.001	0.126	<0.001	**<0.0001**
		f.reg.			0.002	0.16	0.098	<0.001	0.51
		COMBAT			0.002	0.19	0.111	<0.001	0.59

Table 2. Coefficients-of-determination (R^2), the corresponding p-values and the p-values of the partial confounder tests, for all investigated datasets, confounders (conf.) and confounder-mitigation methods (method). Bold numbers denote significant confounding bias identified by the partial confounder test.

of the simulated and empirical experiments was, therefore, not to justify the validity of the approach but to (i) test the software implementation, (ii) estimate statistical power in various situations and (ii) exemplify how the partial confounder test can be used with real experimental data. The validity of the type I error control and was confirmed by our simulations, even if both the predictions and the confounder are non-normally and/or non-linearly dependent on the target variable (except by extreme non-normality). While different biomedical applications may consider different amounts of bias to be relevant, in most cases it is possible to set an upper bound for confounding bias that is still tolerable in certain applications. The simulation results can serve as a basis for power calculations in these cases, in order to identify the necessary sample size for proper model diagnostics.

A characteristic example for the potential areas of applications is the novel field of population neuroscience, where applying predictive modelling and machine learning on large-scale functional neuroimaging data holds great potential for both revolutionizing our understanding of the physical basis of mind and delivering clinically useful tools for diagnostics or therapeutic decision making [5, 23, 3, 25]. However, the presence of confounders that are typical for biomedical research (e.g. sample demographics, center-effects) or specific to the data acquisition and processing approach (e.g. imaging artifacts) presents a great challenge to these effort [29]. The usefulness of the proposed tests is demonstrated in two such examples, using the HCP [55] and the ABIDE [62] datasets.

In case of the HCP dataset, the statistically significant age bias of the 'raw' model for predicting fluid intelligence is in line with previous findings [18, 19] and could likely exaggerate to a serious bias when testing the model on data of participants outside of the - relatively narrow - age range of the HCP sample. In this case, the bias would likely significantly harm the out-of-sample generalizability of this model. The bias of the same model for acquisition batch can also be problematic, especially as it has not yet been thoroughly discussed in case of the HCP dataset. There can be manifold reasons for the observed acquisition bias. Fluid intelligence of the included participants might be, for instance, affected by a changing selection bias during participant recruitment (e.g. as a consequence of the human connectome project receiving an increasing degree of public interest during its course).

In the ABIDE dataset, neither the center bias nor the age bias is surprising in the case of the 'raw' model but both would be obviously severely problematic for a diagnostic biomarker candidate of ASD. For instance the model trained on the raw (unadjusted) features - depending on the calibration of the predicted class probabilities - might classify all participants form e.g. the CMU (Carnegie Mellon University) center as neurotypical control participants. Similarly, the models biased by motion - next to having questionable neuroscientific validity - might systematically fail in populations with a tendency for higher in-scanner motion (as known for many conditions, among others ADHD [10] or Alzheimer's disease [9]).

The partial confounder test provided quantitative, statistically rigorous metrics for assessing the effectiveness of the investigated confounder mitigation techniques. In the HCP data, it revealed that both the acquisition bias and the age bias was very effectively removed by both feature regression and COMBAT ($p>0.05$ for all). Given the high power of the test at the sample sizes of the HCP dataset ($N = 999$), any remaining confounding bias is most probably very safely negligible and well out of the range of practical relevance.

The confound mitigation approaches performed well in attenuating motion bias in the ABIDE dataset, as well, as no residual bias was detected by the proposed test. However, the success of COMBAT in eliminating motion bias is not to be taken without any objections. As COMBAT was originally developed for harmonizing effects of categorical variables (e.g. center or batch), its application for continuous confounder variables is not trivial. Inputting discretized versions of continuous variables into COMBAT might be sub-optimal and raises further questions e.g regarding the optimal number of bins used during the discretization.

Importantly, the partial confounder test revealed, that the center bias of the classification in the massively multi-center ABIDE dataset was, although mitigated, but not successfully removed by COMBAT. While determining the relevance of the remaining bias is out of the scope of this paper, the example demonstrates the need for checking confounder bias even if state-of-the-art confounder mitigation approaches have been applied. If the proposed test provides evidence for residual confounding bias, the researcher might consider the use of another mitigation approach (e.g. feature regression in the given case) or the evaluation of confound-free performance e.g. via 'confound-isolating cross-validation' [29].

In sum, the application of the partial confounder test on the real data examples suggests that confounding bias must be always carefully investigated and reported in studies utilizing predictive modelling and machine learning as (i) variables as trivial as the date of the acquisition can cause significant confounding bias and (ii) in certain situations, state-of-the-art confoudner mitigation techniques may not provide suffi-

cient mitigation of confounding bias and (iii) unnecessary confounder correction may eliminate variance-of-interest. As the proposed test is a model-agnostic post-hoc test, it can be used to benchmark different machine learning models and to further characterize already trained models in external validation samples, where a larger set of potential confounder variables is available (Supplementary Figure 13). The partial confounder test can be considered as a useful, objective benchmark to guide the search for a suitable confounder mitigation approach for every dataset.

Conclusion

The lack of rigorous statistical tests for confounding bias significantly hampers the development of predictive models in many fields of research, including population neuroscience, where handling confounding effects is especially challenging [23].

To fill this critical gap in predictive model development, here I proposed two novel tests, the *partial* and the *full confounder tests*, which probe the null hypotheses of 'no confounding bias' and 'full confounding bias', respectively. The tests are distinguished from alternative approaches by their robustness to non-normally and non-linearly dependent predictions, rendering them applicable with a wide variety of machine learning models. The tests have, moreover, a minimal computational overhead, as re-fitting the model is not required.

As demonstrated on functional brain connectivity-based predictive models of fluid intelligence and autism spectrum disorder, the tests can guide the optimization of confound mitigation strategies and allow quantitative statistical assessment of the robustness, generalizability and neurobiological validity of predictive models in biomedical research. Given their simplicity, robustness, wide applicability, high statistical power and computationally effective implementation (available in the python package *mlconfound*[7]), the partial and full confounder tests emerge as novel tools in the methodological arsenal of predictive modelling and may largely accelerate the development of clinically useful machine learning biomarkers.

Declarations

Data Availability

Empirical analysis was based on preprocessed data provided by the Human Connectome Project, WU-Minn Consortium [55] (principal investigators: D. Van Essen and K. Ugurbil; 1U54MH091657) funded by the 16 NIH institutes and centers that support the NIH Blueprint for Neuroscience Research; and by the McDonnell Center for Systems Neuroscience at Washington University and the Autism Brain Imaging Data Exchange (ABIDE) consortium [62].

All data used in the present study are available for download from the Human Connectome Project (www.humanconnectome.org). Users must agree to data use terms for the HCP before being allowed access to the data and ConnectomeDB; details are provided at https://www.humanconnectome.org/study/hcp-young-adult/data-use-terms. Python implementation of the 'mlconfound' package is available on github. All analysis code is available at github and via the GigaScience database GigaDB [69].

7 https://mlconfound.readthedocs.io

Funding

This research was supported by the Deutsche Forschungsgemeinschaft (DFG, German Research Foundation) – Projektnummer 316803389 – SFB 1280 and TRR 289 Treatment Expectation - Projektnummer 422744262.

Competing Interests

The authors declare that they have no competing interests.

Acknowledgement

I am thankful to Ulrike Bingel (University Hospital Essen, Germany) and Robert Englert (University Hospital Essen, Germany) for their valuable insights and comments on the manuscript. I show appreciation to the contributors of Human Connectome Project and the Autism Brain Imaging Exchange study for collecting and sharing the quality data to researchers.

List of abbreviations

- ABIDE: Autism Brain Imaging Data Exchange
- ASD: Autism Spectrum Disorder
- AUC: Area under the curve
- COMBAT: "Combatting batch effects" data harmonization approach
- CPT: conditional permutation testing
- DX: diagnosis
- FD: framewise displacement
- GAM: generalized additive model
- Gf: fluid intelligence
- HCP: Human Connectome Project
- MCMC: Markov-chain Monte-Carlo
- ROC: receiver operator curve

References

1. Vogt N. Machine learning in neuroscience. Nature Methods 2018;15(1):33–33.
2. Kent DM, Steyerberg E, van Klaveren D. Personalized evidence based medicine: predictive approaches to heterogeneous treatment effects. Bmj 2018;363.
3. Spisak T, Kincses B, Schlitt F, Zunhammer M, Schmidt-Wilcke T, Kincses ZT, et al. Pain-free resting-state functional brain connectivity predicts individual pain sensitivity. Nature communications 2020;11(1):1–12.
4. Walsh I, Fishman D, Garcia-Gasulla D, Titma T, Pollastri G, Harrow J, et al. DOME: recommendations for supervised machine learning validation in biology. Nature methods 2021;p. 1–6.
5. Woo CW, Chang LJ, Lindquist MA, Wager TD. Building better biomarkers: brain models in translational neuroimaging. Nature neuroscience 2017;20(3):365–377.
6. Obermeyer Z, Powers B, Vogeli C, Mullainathan S. Dissecting racial bias in an algorithm used to manage the health of populations. Science 2019;366(6464):447–453.
7. Mehrabi N, Morstatter F, Saxena N, Lerman K, Galstyan A. A survey on bias and fairness in machine learning. ACM Computing Surveys (CSUR) 2021;54(6):1–35.
8. Prosperi M, Guo Y, Sperrin M, Koopman JS, Min JS, He X, et al. Causal inference and counterfactual prediction in machine learning for actionable healthcare. Nature Machine Intelligence 2020;2(7):369–375.
9. Rao A, Monteiro JM, Mourao-Miranda J, Initiative AD, et al.

Predictive modelling using neuroimaging data in the presence of confounds. NeuroImage 2017;150:23–49.

10. Eloyan A, Muschelli J, Nebel MB, Liu H, Han F, Zhao T, et al. Automated diagnoses of attention deficit hyperactive disorder using magnetic resonance imaging. Frontiers in systems neuroscience 2012;6:61.
11. Couvy-Duchesne B, Ebejer JL, Gillespie NA, Duffy DL, Hickie IB, Thompson PM, et al. Head motion and inattention/hyperactivity share common genetic influences: implications for fMRI studies of ADHD. PloS one 2016;11(1):e0146271.
12. Gotts SJ, Saad ZS, Jo HJ, Wallace GL, Cox RW, Martin A. The perils of global signal regression for group comparisons: a case study of Autism Spectrum Disorders. Frontiers in human neuroscience 2013;7:356.
13. Spisak T, Jakab A, Kis SA, Opposits G, Aranyi C, Berenyi E, et al. Voxel-wise motion artifacts in population-level whole-brain connectivity analysis of resting-state FMRI. PloS one 2014;9(9):e104947.
14. Spisak T, Kincses B, Bingel U. Optimal choice of parameters in functional connectome-based predictive modelling might be biased by motion: comment on Dadi et al. bioRxiv 2019;p. 710731.
15. Orban C, Kong R, Li J, Chee MW, Yeo BT. Time of day is associated with paradoxical reductions in global signal fluctuation and functional connectivity. PLoS biology 2020;18(2):e3000602.
16. Cole MW, Yarkoni T, Repovš G, Anticevic A, Braver TS. Global connectivity of prefrontal cortex predicts cognitive control and intelligence. Journal of Neuroscience 2012;32(26):8988–8999.
17. He T, Kong R, Holmes AJ, Nguyen M, Sabuncu MR, Eickhoff SB, et al. Deep neural networks and kernel regression achieve comparable accuracies for functional connectivity prediction of behavior and demographics. NeuroImage 2020;206:116276.
18. Dubois J, Galdi P, Paul LK, Adolphs R. A distributed brain network predicts general intelligence from resting-state human neuroimaging data. Philosophical Transactions of the Royal Society B: Biological Sciences 2018;373(1756):20170284.
19. Lohmann G, Lacosse E, Ethofer T, Kumar VJ, Scheffler K, Jost J. Predicting intelligence from fMRI data of the human brain in a few minutes of scan time. bioRxiv 2021;.
20. Lwowski B, Rios A. The risk of racial bias while tracking influenza-related content on social media using machine learning. Journal of the American Medical Informatics Association 2021;28(4):839–849.
21. Li J, Bzdok D, Holmes A, Yeo T, Genon S. Not one model fits all: unfairness in RSFC-based prediction of behavioral data in African American. Helmholtz AI kick-off meeting 2020;.
22. Paulus MP, Thompson WK. Computational approaches and machine learning for individual-level treatment predictions. Psychopharmacology 2021;238(5):1231–1239.
23. Smith SM, Nichols TE. Statistical challenges in "big data" human neuroimaging. Neuron 2018;97(2):263–268.
24. Wachinger C, Rieckmann A, Pölsterl S, Initiative ADN, et al. Detect and correct bias in multi-site neuroimaging datasets. Medical Image Analysis 2021;67:101879.
25. Nunes A, Schnack HG, Ching CR, Agartz I, Akudjedu TN, Alda M, et al. Using structural MRI to identify bipolar disorders–13 site machine learning study in 3020 individuals from the ENIGMA Bipolar Disorders Working Group. Molecular psychiatry 2020;25(9):2130–2143.
26. Dukart J, Schroeter ML, Mueller K, Initiative ADN. Age correction in dementia–matching to a healthy brain. PloS one 2011;6(7):e22193.
27. Abdulkadir A, Ronneberger O, Tabrizi SJ, Klöppel S. Reduction of confounding effects with voxel-wise Gaussian process regression in structural MRI. In: 2014 International Workshop on Pattern Recognition in Neuroimaging IEEE; 2014. p. 1–4.
28. Johnson WE, Li C, Rabinovic A. Adjusting batch effects in microarray expression data using empirical Bayes methods. Biostatistics 2007;8(1):118–127.
29. Chyzhyk D, Varoquaux G, Milham M, Thirion B. How to remove or control confounds in predictive models, with applications to brain biomarkers. GigaScience 2022;11.
30. Dockès J, Varoquaux G, Poline JB. Preventing dataset shift from breaking machine-learning biomarkers. GigaScience 2021;10(9):giab055.
31. Korn EL. The ranges of limiting values of some partial correlations under conditional independence. The American Statistician 1984;38(1):61–62.
32. Bergsma W. Nonparametric testing of conditional independence by means of the partial copula. Available at SSRN 1702981 2010;.
33. Candès E, Fan Y, Janson L, Lv J. Panning for gold: Model-X knockoffs for high-dimensional controlled variable selection. arXiv preprint arXiv:161002351 2016;.
34. Peters J, Bühlmann P, Meinshausen N. Causal inference by using invariant prediction: identification and confidence intervals. Journal of the Royal Statistical Society Series B (Statistical Methodology) 2016;p. 947–1012.
35. Shah RD, Peters J. The hardness of conditional independence testing and the generalised covariance measure. The Annals of Statistics 2020;48(3):1514–1538.
36. Berrett TB, Wang Y, Barber RF, Samworth RJ. The conditional permutation test for independence while controlling for confounders. Journal of the Royal Statistical Society: Series B (Statistical Methodology) 2020;82(1):175–197.
37. García S, Fernández A, Luengo J, Herrera F. A study of statistical techniques and performance measures for genetics-based machine learning: accuracy and interpretability. Soft Computing 2009;13(10):959.
38. Kristensen SB, Sandberg K. Is whole-brain functional connectivity a neuromarker of sustained attention? Comment on Rosenberg & al.(2016). bioRxiv 2017;p. 216697.
39. Neto CE, Pratap A, Perumal TM, Tummalacherla M, Bot BM, Mangravite L, et al. A permutation approach to assess confounding in machine learning applications for digital health. In: Proceedings of the 25th ACM SIGKDD International Conference on Knowledge Discovery & Data Mining; 2019. p. 54–64.
40. Ferrari E, Retico A, Bacciu D. Measuring the effects of confounders in medical supervised classification problems: the Confounding Index (CI). Artificial intelligence in medicine 2020;103:101804.
41. Southworth LK, Kim SK, Owen AB. Properties of balanced permutations. Journal of Computational Biology 2009;16(4):625–638.
42. Hemerik J, Goeman J. Exact testing with random permutations. Test 2018;27(4):811–825.
43. Dawid AP. Conditional independence in statistical theory. Journal of the Royal Statistical Society: Series B (Methodological) 1979;41(1):1–15.
44. Spirtes P, Glymour CN, Scheines R, Heckerman D. Causation, prediction, and search. MIT press; 2000.
45. Fiedler K, Schott M, Meiser T. What mediation analysis can (not) do. Journal of Experimental Social Psychology 2011;47(6):1231–1236.
46. Pitman EJ. Significance tests which may be applied to samples from any populations. Supplement to the Journal of the Royal Statistical Society 1937;4(1):119–130.
47. Fisher R. The Theory of Confounding in Factorial Experi-

ments in Relation to the Theory of Groups. Contributions to Mathematical Statistics 1942;.
48. Hastie T, Tibshirani R. Generalized additive models: some applications. Journal of the American Statistical Association 1987;82(398):371–386.
49. Bennett B. Multiple Regression Analysis of Binary and Multinomial Variates. Sankhyā: The Indian Journal of Statistics, Series A 1966;p. 301–304.
50. Jones RH. Proability estimation usind a multinominal logistic function. Journal of Statistical Computation and Simulation 1975;3(4):315–329.
51. Chambers M, Dinsmore TW. Advanced analytics methodologies: Driving business value with analytics. Pearson Education; 2014.
52. Servén D, Brummitt C, Abedi H. pyGAM: Generalized Additive Models in Python. Zenodo 2018;.
53. Starkweather J, Moske AK, Multinomial logistic regression; 2011.
54. Jones MC, Pewsey A. Sinh-arcsinh distributions. Biometrika 2009;96(4):761–780.
55. Van Essen DC, Smith SM, Barch DM, Behrens TE, Yacoub E, Ugurbil K, et al. The WU-Minn human connectome project: an overview. Neuroimage 2013;80:62–79.
56. Glasser MF, Sotiropoulos SN, Wilson JA, Coalson TS, Fischl B, Andersson JL, et al. The minimal preprocessing pipelines for the Human Connectome Project. Neuroimage 2013;80:105–124.
57. Duncan J, Seitz RJ, Kolodny J, Bor D, Herzog H, Ahmed A, et al. A neural basis for general intelligence. Science 2000;289(5478):457–460.
58. Beasley TM, Erickson S, Allison DB. Rank-based inverse normal transformations are increasingly used, but are they merited? Behavior genetics 2009;39(5):580–595.
59. Pedregosa F, Varoquaux G, Gramfort A, Michel V, Thirion B, Grisel O, et al. Scikit-learn: Machine learning in Python. the Journal of machine Learning research 2011;12:2825–2830.
60. Fortin JP, Cullen N, Sheline YI, Taylor WD, Aselcioglu I, Cook PA, et al. Harmonization of cortical thickness measurements across scanners and sites. Neuroimage 2018;167:104–120.
61. Hoerl AE, Kennard RW. Ridge regression: applications to nonorthogonal problems. Technometrics 1970;12(1):69–82.
62. Di Martino A, Yan CG, Li Q, Denio E, Castellanos FX, Alaerts K, et al. The autism brain imaging data exchange: towards a large-scale evaluation of the intrinsic brain architecture in autism. Molecular psychiatry 2014;19(6):659–667.
63. Dadi K, Rahim M, Abraham A, Chyzhyk D, Milham M, Thirion B, et al. Benchmarking functional connectome-based predictive models for resting-state fMRI. NeuroImage 2019;192:115–134.
64. Craddock C, Benhajali Y, Chu C, Chouinard F, Evans A, Jakab A, et al. The neuro bureau preprocessing initiative: open sharing of preprocessed neuroimaging data and derivatives. Frontiers in Neuroinformatics 2013;7.
65. Bellec P, Rosa-Neto P, Lyttelton OC, Benali H, Evans AC. Multi-level bootstrap analysis of stable clusters in resting-state fMRI. Neuroimage 2010;51(3):1126–1139.
66. Huntenburg J, Abraham A, Loula J, Liem F, Dadi K, Varoquaux G. Loading and plotting of cortical surface representations in Nilearn. Research Ideas and Outcomes 2017;3:e12342.
67. Estève L. Big data in practice: the example of nilearn for mining brain imaging data. In: Scipy 2015; 2015. .
68. Power JD, Mitra A, Laumann TO, Snyder AZ, Schlaggar BL, Petersen SE. Methods to detect, characterize, and remove motion artifact in resting state fMRI. Neuroimage 2014;84:320–341.
69. Spisak T. Supporting data for "Statistical quantification of confounding bias in machine learning models". GigaScience Database 2022;http://dx.doi.org/10.5524/102244.

Manuscript

Click here to access/download;Manuscript;mlconfound_revised.pdf

OXFORD (GIGA)^n SCIENCE

GigaScience, 2022, 1–14

doi: xx.xxxx/xxxx
Manuscript in Preparation
Paper

PAPER

Statistical quantification of confounding bias in machine learning models

Tamas Spisak [1,*]

[1]Center for Translational Neuro- and Behavioral Sciences, Institute for Diagnostic and Interventional Radiology and Neuroradiology, Center , University Hospital Essen

*tamas.spisak@uk-essen.de

Abstract

Background: The lack of non-parametric statistical tests for confounding bias significantly hampers the development of robust, valid and generalizable predictive models in many fields of research. Here I propose the *partial confounder test*, which, for a given confounder variable, probes the null hypotheses of the model being *unconfounded*. **Results:** The test provides a strict control for Type I errors and high statistical power, even for non-normally and non-linearly dependent predictions, often seen in machine learning. Applying the proposed test on models trained on large-scale functional brain connectivity data (N=1865) (i) reveals previously unreported confounders and (ii) shows that state-of-the-art confound mitigation approaches may fail preventing confounder bias in several cases. **Conclusions:** The proposed test (implemented in the package *mlconfound*[1]) can aid the assessment and improvement of the generalizability and validity of predictive models and, thereby, fosters the development of clinically useful machine learning biomarkers.

Key words: machine learning; predictive modelling; confounding bias; confounder test; conditional independence; conditional permutation

Background

Predictive modelling has recently become increasingly important in biomedical research and holds promise for delivering biomarkers that substantially impact clinical practice and public health [1, 2, 3, 4]. When evaluating the usefulness and applicability of such markers, predictive performance is far from being the only important consideration. Biomedical validity and generalizability across contexts and populations are also fundamental requirements for candidate biomarkers [5, 6, 7].

Spurious, out-of-interest associations between the predictor variables (features) and the prediction target can be detrimental to the model's biomedical validity and generalizability. This phenomenon is often called confounding bias [8]. Confounding bias can be driven by various sources. For instance, measurement artifacts, e.g motion artifacts in magnetic resonance imaging-based predictive models, are well known as a potential confounder that can bias the predictive model's output in, among others, Alzheimer's [9], attention deficit hyperactivity disorder [10, 11] or Autism Spectrum Disorder (ASD) [12, 13, 14]). Confounding bias is however not restricted to measurement artifacts. Depending on the research question, several demographic and psychometric variables, or the time of day of the data acquisition [15] can emerge as confounders. As a characteristic example, models trained to predict intelligence [16, 17] might provide a statistically significant predictive performance by picking up solely on age-related variance [18, 19]). Moreover, various types of systematic sampling bias, as well as stochastic group differences in the training sample, can result in confounded models (e.g. racially biased machine learning models [6, 20, 21]).

Confounding-bias is especially problematic in population neuroscience studies. While large-scale multi-site studies are of key importance for developing robust machine learning markers [22], most of the confounding effects are much more likely to occur in such big, longer-term studies [23] and batch and center effects may arise as additional sources of confounding bias [24, 25].

While various data cleaning methods and dedicated prediction algorithms may help in mitigating confounding bias [9, 26, 13, 27, 28, 29], effects of confounders can potentially bleed through into

Compiled on: July 27, 2022.
Draft manuscript prepared by the author.

Key Points

- The lack of statistical tests for confounding bias hampers the development of machine learning based biomarker candidates
- The 'partial confounder test' provides a model-agnostic approach for quantifying confounding bias
- It provides strict control for type-I errors and high statistical power with minimal assumptions
- Deploying the test on functional brain connectivity data reveals that confounding bias can be problematic even if confound mitigation approaches are used
- The test provides objective criteria to assess the specificity, generalizability and biomedical validity of biomarker candidates

predictions even if they are being attempted to control for in the prediction algorithm (see Supplementary Analysis 1 for an example) and it is often unclear which variables should be considered as confounders. In a number of cases, removing or controlling for a confounder can remove variance-of-interest and complicate model interpretations [24, 29, 30], rendering the choice of confound mitigation strategy as one of the most difficult compromises in predictive model development.

Powerful and robust statistical tests for quantifying confounding bias in predictive models could substantially foster both the identification of confounders to correct for and the assessment of the effectiveness of various confound-mitigation approaches. It is tempting to think about confounding bias as the *conditional dependence* of the model output on the observed confounder, given the target variable. However, the proper evaluation of conditional independence among these variables is challenging. Namely, even in the presence of a slight non-normality and/or non-linearity of the involved conditional distributions, the 'conditional' analogs of the most popular bivariate non-parametric tests (like the partial Spearman correlation, see Fig. 3) are not valid measures of conditional independence. Although warnings about this issue were given from early on [31], and received a fair amount of attention recently [32, 33, 34, 35, 36], the magnitude of the problem may not be fully appreciated in case of predictive model diagnostics, where non-normality and non-linearity of the model output can be frequently seen (see Supplementary Figures S10-11), as a consequence of e.g. feature-set characteristics and model regularization [37, 38].

Recently, two different approaches were proposed for quantifying confounding bias [39, 40]. However, these methods either fail to control type I error (as known in the case of balanced permutations [41, 42], used in ref. [39]), or do not provide p-values at all [40]. Moreover, without some modifications, they are only applicable for categorical variables and involve re-fitting the model, which may not be feasible for models with high computational cost (e.g. when trained with nested cross-validation).

This work aims to construct a statistical test for confounding bias that (i) guarantees valid type-I error control for arbitrary models, even if non-normal and/or non-linear dependencies are involved (ii) does not require re-fitting the model, (iii) is applicable for classification as well as for prediction problems and both with numerical and categorical confounders.

Methods

Notation and Background

In a predictive modelling setting, let y denote the target variable, $\mathbf{X}$ denote the feature variables, $\hat{y}$ denote model output, i.e. the predictions for y and let c denote a variable which is considered as a confounder. Note, that y and c must be observed during the experiment, whereas $\hat{y}$ is provided by the predictive model. Confounding bias typically emerges in situations where $\mathbf{X} \leftarrow c \rightarrow y$ (arrows denoting dependence of $\mathbf{X}$ and y on c), although $c \rightarrow y$ is not a prerequisite. After fitting the predictive model, we aim to construct predictions based on features unseen during the model training procedure: $\mathbf{X} \rightarrow \hat{y}$ so that $y \rightarrow \hat{y}$. Obviously, a strong association between $\hat{y}$ and c may indicate that the model is biased; its predictions are driven by the confounder rather than information about the target variable. Assessing the simple bivariate (unconditioned) dependence ($H0 : \hat{y} \perp\!\!\!\perp c$) between $\hat{y}$ and c (or any of the y, $\hat{y}$, c variables) is, however, insufficient for the proper characterization of confounding bias in predictive modelling. For instance, even if $\hat{y} \perp\!\!\!\perp c$ is false, $\hat{y}$ might be only marginally dependent on c, due to the dependence of both on y. In other words, if the target variable y displays a true association to the confounder variable c, a model that is completely blind to c (i.e not confounded at all) might still provide outputs $\hat{y}$ that are significantly associated with c.

Conditional independence for testing confounding bias

Instead of focusing on the 'unconditioned' independence between the confounder and the predictions, we shall consider the *conditional independence* between $\hat{y}$ and c given y (written as $\hat{y} \perp\!\!\!\perp c|y$) which, by definition [43], means that $\mathbb{P}(\hat{y}, c|y) = \mathbb{P}(\hat{y}|y)\mathbb{P}(c|y)$. Testing whether c is independent from $\hat{y}$, conditional on y, is essentially checking whether the path $c \rightarrow \mathbf{X} \rightarrow \hat{y}$ has been blocked in the prediction algorithm. The statistical test with the null hypothesis $H0 : \hat{y} \perp\!\!\!\perp c|y$ will be referred to as the *partial confounder test*. Of note, although typically less useful in a predictive modelling context, one might also be interested in testing $\hat{y} \perp\!\!\!\perp y|c$. We refer to the corresponding test as the *full confounder test*.

Conditional independence - in its general form - is a fundamental concept in statistics with numerous biomedical applications [44, 45, 34, 33]. Recently, [35] have raised important concerns regarding conditional independence testing. Their "no free lunch" theorem implies that, without placing some assumptions on the joint distribution of $(y, \hat{y}, c)$, conditional independence testing is effectively impossible. In other words, neither the full nor the partial confounder tests can be constructed so that - for all distributions - they provide a valid type I error control and, at the same time, a non-trivial statistical power.

This result stands in strong contrast to *unconditional* independence testing - where permutation tests [46, 47], provide a general, distribution-free solution - and it has important implications for confounder testing in predictive modelling where the distribution of the model outputs (conditioned on the target variable) - depending on the applied machine learning model - is unknown and often non-normal and non-linear. One of the trivial candidates for the task, partial correlation, for instance assumes that all involved variables are multivariate Gaussian and - as to be shown below in a simulated example - even its Spearman-based variant is unable to tolerate relatively small deviations from normality and linearity.

Recently, Candès et al. [33], and, based on their work, Berrett et al. [36], have demonstrated that valid and powerful conditional independence tests can be constructed with inputting distributional information about only two (out of the three) variables. Specifically, the conditional permutation test (CPT) of Berrett and colleagues samples from a non-uniform distribution over the set of possible permutations π of one of the variables, based on its conditional distribution of the other variable. Thereby, it incorporates the infor-

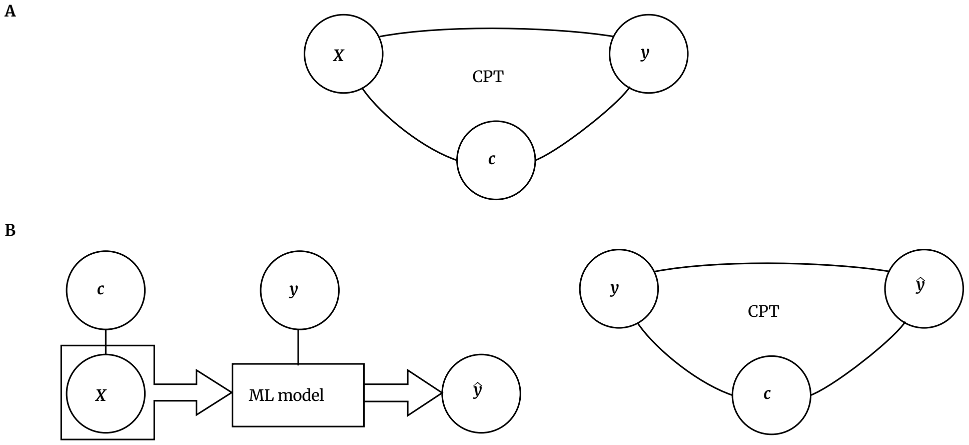

Figure 1. Conditional permutation testing as a tool for predictive model diagnostics.
(A) Conditional Permutation testing (CPT) was originally proposed to be used on the feature variable X, target variable Y and confounders Z, to perform statistical inference. **(B)** The proposed use of CPT in predictive modelling requires the model to be fitted first, to obtain the model's prediction $\hat{y}$ on y. CPT is then utilized on the triplet $(y, \hat{y}, c)$, to test hypotheses $\hat{y} \perp\!\!\!\perp c|y$ or $y \perp\!\!\!\perp \hat{y}|c$. using CPT this way allows lifting assumptions on the prediction target. However, as shown on Fig. 3 the original, can still provide inflated p-values in case of non-linearity in the conditional distributions. False positives can be successfully eliminated by the proposed non-linear techniques for conditional distribution modelling (Fig. 2.)

		H0	assumption needed for:	no assumptions about the distribution of:
**1.**	$\hat{y} \perp\!\!\!\perp y\|c$	full confounder test: model exclusively driven by the confounder	$Q(y\|c)$	$(\hat{y}, y), (\hat{y}, c)$
**2.**	$y \perp\!\!\!\perp c\|\hat{y}$	model captures all variance in the confounder (not of interest)	$Q(c\|\hat{y})$	$(y, c), (y, \hat{y})$
**3.**	$\hat{y} \perp\!\!\!\perp c\|y$	partial confounder test: model not directly driven by the confounder	$Q(c\|y)$	$(\hat{y}, c), (\hat{y}, y)$

Table 1. Possibilities when testing conditional independence in potentially biased predictive models.
The table lists the three possible null hypotheses (H0), and the variables where assumption about the joint/conditional distributions is required/not required. (y: prediction target, $\hat{y}$: predictions, c: confounder variable)

mation available about the conditional distribution of interest into the permutation-based inference in a statistically valid manner.

Like many related papers, the work of Berrett et al. was formalized as a (semi-)supervised learning approach, where X is a set of predictors (features), y is the target variable and c is a potential confounder (Figure 1A). In this setting, testing the null hypothesis $X \perp\!\!\!\perp y|c$ aims to determine, whether the features X still affect y, when controlling for c. For instance, in genome-wide association studies, CPT can be used to determine whether a particular genetic variant X affects a response y such as disease status or some other phenotype, even after controlling for the rest of the genome, encoded in c.

In this paper, a different setting is considered, where the supervised learning model is already fitted (Figure 1B) and we are focusing on model diagnostics, by testing the triplet $(y, \hat{y}, c)$, with the requirement of minimal assumptions on the conditional distribution of $\hat{y}$ on y and c (Figure 1C).

Within this setting, conditional independence testing and, specifically, the framework of conditional permutation testing allows investigating three different null hypotheses corresponding to the $(y, \hat{y}, c)$ triplet. As listed in Table 1, testing the null hypothesis $y \perp\!\!\!\perp \hat{y}|c$ (option 1, full confounder testing) investigates whether the predictions are likely explainable solely with the confounder, i.e. whether the model is exclusively confounder-driven. Testing $y \perp\!\!\!\perp c|\hat{y}$ (option 2) addresses the question whether the model captures all the variance in c when predicting y. Testing the null hypothesis $\hat{y} \perp\!\!\!\perp c|y$ (option 3, partial confounder testing) examines, whether the dependence of the model output on the confounder can likely be explained by the confounder's dependence on the target variable, i.e. whether there is any confounding bias in the model.

Option 3, i.e. partial confounder testing is typically of interest when testing confounding bias of predictive models. Option 1, i.e. full confounder testing may be less useful in practice, although it might provide valuable insights in the exploratory phase of model construction. Option 2 does not seem appealing for model diagnostics and importantly, in this case the proposed variety of the CPT framework does not allow constructing a test which is non-parametric on $\hat{y}$. We will therefore focus on option 3, i.e. the partial confounder test.

In the following section, CPT is adapted for *partial* confounder testing and extended with general additive model [48] (GAM) and multinomial logistic regression [49, 50] based conditional distribution estimations, in order to make it handle categorical data and non-linear dependencies between the confounder and the target variable. (For an overview of the method, see Fig. 2).

The partial confounder test

The inner workings of the *partial confounder test* are summarized on Fig. 2. In short, the test models the conditional distribution between the confounder and the target variable with a GAM - or with an *mnlogit* regression, in case of categorical confounder - and then uses a so-called parallel-pairwise Markov-chain Monte-Carlo sampler of [36] that draws permutations of the original confounder, so that the permuted variables still comply with the estimated conditional distribution. As a result, the permuted "copies" of the confounder variable retain its correlation with the target variable but eliminate any "additional" relationship with the model output. The test statistic (coefficient of determination, R^2) is then computed between the model output and the original, as well as the permuted confoudner variables. The original and the permuted test statistics construct the p-value as the ratio of permuted test

statistics more extreme than the original.

In detail, the partial confounder test generates a null-distribution for an arbitrary predefined test statistic $T(y, \hat{y}, c)$ by sampling permutation based 'copies' of the original c,

$$c_i^{(j)} \sim Q(\cdot|y_i) \tag{1}$$

where, $Q(.|y)$ denotes the conditional distribution of c given $y = y_i$ and $j = 1, \ldots, m$ indexes the 'copy' of c so that

$$c^{(j)} = (c_1^{(j)}, \ldots, c_n^{(j)}) = (c_{\pi_1^{(j)}}, \ldots, c_{\pi_n^{(j)}}) = c_{\pi^{(j)}}$$

is a permutation of the original vector $c = (c_1, \ldots, c_n)$, with its elements reordered according to the permutation $\pi \in S_n$ where S_n denote the set of all permutations on the indices $\{1, \ldots, n\}$.

As shown by [36], to ensure that Eq. 1 holds, the $c_{\pi^{(j)}}$ copies must be drawn so that:

$$\mathbb{P}(\pi^{(j)} = \pi | y, \hat{y}, c) = \frac{q^n(c_\pi | y)}{\sum_{\pi' \in S_n} q^n(c_{\pi'} | y)} \tag{2}$$

that is, according to the $q^n(\cdot|y) := q(\cdot|y_1) \ldots q(\cdot|y_n)$ product density corresponding to the conditional distribution $Q(\cdot|y)$. Note that Eq. 2 does not necessarily assume a continuous distribution.

This mechanism creates copies $c^{(1)}, \ldots, c^{(m)}$ so that under the null hypothesis $(\hat{y} \perp\!\!\!\perp c | y)$, the triples

$$(y, \hat{y}, c), (y, \hat{y}, c^{(1)}), \ldots, (y, \hat{y}, c^{(m)})$$

are all identically distributed and so are the

$$T(y, \hat{y}, c), T(y, \hat{y}, c^{(1)}), \ldots, T(y, \hat{y}, c^{(m)})$$

test statistics, as well.

As long as the numerator of Eq. 2 is non-zero for all $c_\pi \in C$ and $y \in Y$, the conditional permutations constitute an algebraic group, thus, as shown by Hemerik and Goeman [42], an unbiased estimate of the p-value under the null can be obtained as:

$$p = \frac{\sum_{j=1}^{m} \mathbb{1}\{T(y, \hat{y}, c^{(j)}) \geq T(y, \hat{y}, c)\}}{m}$$

While the group property of the conditioned permutations provides a straightforward proof for the validity of the approach, for an alternative verification see the proof of Theorem 1 in [36].

The required permutations could be theoretically sampled with a simple Metropolis-Hastings algorithm that draws uniformly from S_n at random. However, this way the acceptance ratio would be extremely low, even for moderate n (except there is very low dependence of c on y), resulting in slow mixing times. The partial confounder test can be, however, efficiently implemented with the parallelized pairwise Markov-Chain Monte Carlo sampler of [36] (Algorithm 1), that draws disjoint pairs in parallel and decides whether or not to swap them randomly, according to the odds ratio calculated from the conditional densities belonging to the original and swapped data. The acceptance odds ratio of swapping indices i and j is:

$$\ln \frac{q(c_j|y_i)q(c_i|y_j)}{q(c_i|y_i)q(c_j|y_j)} = \ell(c_j|y_i) + \ell(c_i|y_j) - \ell(c_i|y_i) - \ell(c_j|y_j) \tag{3}$$

where ℓ denotes the log-likelihood.

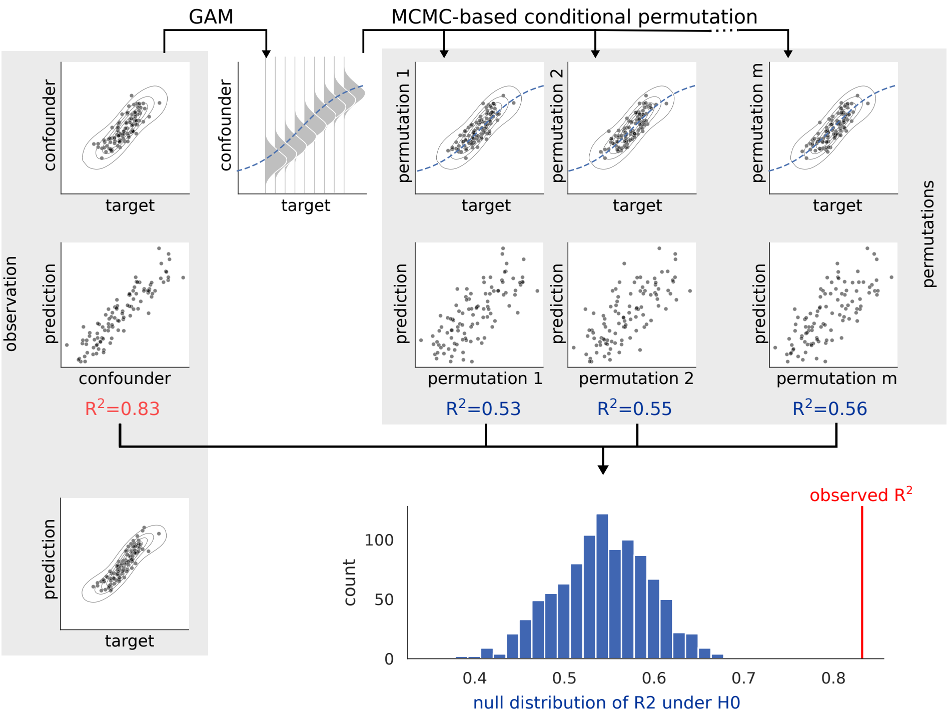

Figure 2. Graphical representation of the proposed partial confounder test.
The partial confounder test models the conditional distribution of the confounder, given the target variable, with a generalized additive model (GAM). The parallel-pairwise Markov-chain Monte-Carlo (MCMC) sampler draws permutations of the original confounder variable that comply with the GAM-based conditional distribution (permutation 1, 2, ..., m). The test statistic (coefficient of determination, R^2) is then computed between the model output and the original, as well as the permuted confounder variables. The original and the permuted test statistics construct the p-value as the ratio of permuted test statistics more extreme than the original. Figure source code available as jupyter notebook: https://github.com/pni-lab/mlconfound-manuscript/blob/main/simulated/overview-fig.ipynb

In their Theorem 2, [36] verify that the resulting Markov Chain yields the desired stationary distribution, even if the number of steps is small.

Conditional log-likelihood

Obtaining a relatively accurate, independent estimate of $Q(\cdot|\boldsymbol{y})$ (of any shape) for CPT inference is important. Berrett and colleagues recommend to use a large independent sample to obtain the log-likelihood matrix that represents the conditional distribution $Q(\cdot|Z)$ or, alternatively, to re-use the data by fitting a least squares linear regression:

$$\boldsymbol{c} = \alpha + \beta\boldsymbol{y} + \boldsymbol{e} \quad (4)$$

As the linear regression-based method, obviously, does not handle non-linear relationships, I propose to apply a modelling approach that accounts for non-linearity. Although several nonparametric techniques might be suitable for this purpose, many of these tend to be greedy for large sample sizes, may lack stability or perform poorly with many potential predictors. Certain methods, such as kernel methods and smoothing splines, are also very difficult to interpret [51]; an important consideration when analyzing the source of a confounder effect.

Here, I propose to use the generalized additive model (GAM) of [48]:

$$\boldsymbol{c} = \alpha + \beta f(\boldsymbol{y}) + \boldsymbol{e} \quad (5)$$

where the feature functions f is built using penalized B-splines, which allow us to automatically model non-linear relationships without having to manually try out many different transformations on each variable. The principal advantages of GAM are that (i) the complexity of the model can be effectively regularized trough its hyperparameters, (ii) it is able to model highly complex nonlinear relationships with a potentially large number of both numeric and categorical predictors and (iii) it has computationally effective solver algorithms. The potential disadvantages of GAMs are not relevant for the problem at hand or can be easily overcame. Specifically, the possibly poor out-of-distribution generalization of GAM is not problematic, as in our approach the model is not used for constructing out-of-distribution predictions. Moreover, as several other models, GAMs can easily overfit the data. However, in the proposed approach, the smoothness of the GAM model is optimized with a grid-search by picking the model with the lowest generalized cross-validation score from the models defined by the default parameters as implemented in PyGAM [52] (v0.8.0).

If we write $\mu = \alpha + \beta f(\boldsymbol{y})$ and σ denotes the standard deviation of the residual $\boldsymbol{e}$, then the conditional distribution of interest can be assumed to be normal with the parameters:

$$(\boldsymbol{c}|\boldsymbol{y} = y_i) \sim \mathcal{N}\{\mu_i, \sigma^2\}$$

and the log-likelihood, that is to be used in Eq. 3, can be computed simply as the log of the corresponding probability density function:

$$\ell(c_i|y_j) = -\frac{1}{2}\left(\frac{c_i - \mu_j}{\sigma}\right)^2 - ln(2\pi\sigma)$$

In the case of categorical $\boldsymbol{c}$, a multinomial logistic regression (*mnlogit*) model can be used to obtain $D(\cdot|\boldsymbol{y})$, with the extra assumption of *complete separation* if $\boldsymbol{y}$ is also categorical (in order to ensure an invertible Hessian, see e.g. [49, 50]).

Importantly, both the GAM- and the *mnlogit*-based approaches guarantee that the numerator of Eq. 2 is always greater than zero and the group property for the permutations holds.

Note that from the three options for conditional independence-based null hypotheses enumerated in Table 1, the proposed approach can not provide a test for option 2 that is assumption-free about $\hat{\boldsymbol{y}}$, as the variable, on which the independence is conditional, must be always the predictor variable in Eq. 5. However, as discussed above, this option is of low practical relevance, anyway. Pleasingly, the proposed Gaussian regression-based conditional likelihood estimation ensures that no assumptions on $\hat{\boldsymbol{y}}$ have to be made for the practically relevant options 1 and 3, i.e. for the full and partial confounder tests.

In theory, any predefined test statistic T can be used with the proposed approach. The python package *mlconfound*, implementing the proposed full and partial confounder tests, utilizes the coefficient of determination (R^2 or pseudo R^2 in case of categorical confounder or classification [53]) as a test statistic: $T(\boldsymbol{y}, \hat{\boldsymbol{y}}, \boldsymbol{c}) = R^2(\hat{\boldsymbol{y}}, \boldsymbol{c})$ and $T(\boldsymbol{y}, \hat{\boldsymbol{y}}, \boldsymbol{c}^{(j)}) = R^2(\hat{\boldsymbol{y}}, \boldsymbol{c}^{(j)})$ which allows interpretable, two tailed inference.

Validation on simulated data

Using CPT to test confounding bias in predictive modelling allows relaxing assumptions on $\hat{\boldsymbol{y}}$ but - in line with the "no free lunch" theorem, requires knowing - or putting assumptions on - the joint distribution of the other two variables ($\boldsymbol{y}$ and $\boldsymbol{c}$). [36] give a detailed analysis of the robustness of their CPT approach when estimating the conditional distribution with re-using the tested data via linear regression and, also, against misspecifying the conditional distribution of interest to introduce non-linearity.

Here I extend these results by performing simulations that evaluate the GAM- and *mnlogit*-based approaches, in a form that is accessible for power calculations in predictive modelling (considering various weights of the target signal in $\boldsymbol{c}$ and the confounder and the target signals in $\hat{\boldsymbol{y}}$). Moreover, I investigate the robustness of the tests against the violation of normality and linearity of the conditional distributions $D(\boldsymbol{c}|\boldsymbol{y})$ and $D(\hat{\boldsymbol{y}}|\boldsymbol{y})$.

Simulations are performed separately for the two proposed tests.

Simulation approach

As a first step, the target variable $\boldsymbol{y}$ is drawn randomly from a normal distribution:

$$\boldsymbol{y} \sim \mathcal{N}(0, 1)$$

Next, the confounder signal is simulated as:

$$\mathrm{c}|y_i \sim f_{\delta,\epsilon}(\mathcal{N}(0,1)) + w_{yc}\, g(y_i)$$

where f is a function to introduce non-normality, namely the *sinh-arcsinh* transformation of [54], defined as:

$$f_{\delta,\epsilon}(\boldsymbol{x}) = sinh(\delta sinh^{-1}(\boldsymbol{x}) - \epsilon)$$

where the parameters δ and ϵ control the kurtosis and skewness of the resulting *sinh-arcsinh* distribution, with $\delta = 1$ and $\epsilon = 0$ producing the identity function (i.e. no non-normality introduced).

Moreover, non-linearity can be introduced with the function g, which can be simply the identity function (no non-linearity is introduced in this case) or, for instance, a sigmoid-shaped function, in our case:

$$g(\boldsymbol{x}) = tanh(\boldsymbol{x})$$

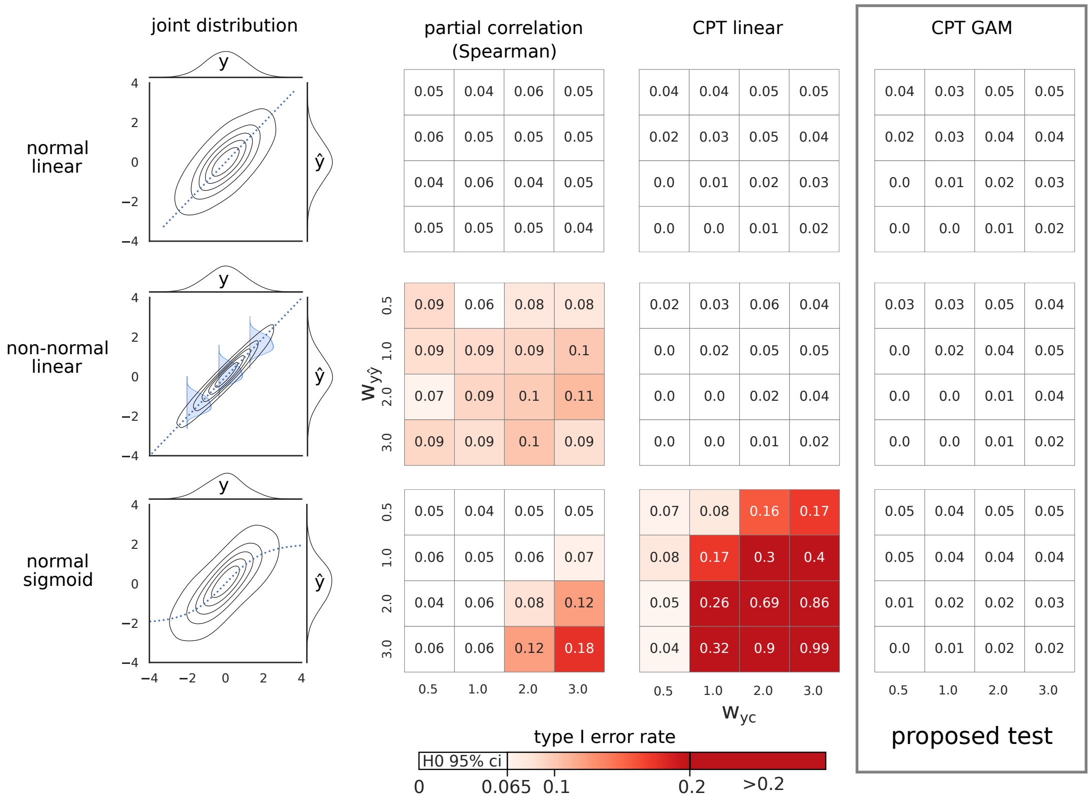

Figure 3. Type I error control of partial Spearman correlation, linear and GAM-based conditional permutation test.
Type I error control was investigated in three example cases: normal conditional distribution with linear dependency (first row), slightly non-normal conditional distribution with linear dependency (second row) and normal conditional distribution with non-normal (sigmoid) dependency (third row). Non-normal conditional distribution on the second plot is illustrated with blue density diagrams (kurtosis: -0.8, skewness: -0.1). False positive rates for confounder contributions (w_{yc}, ranging from 0.5 to 3.0) and predictive performances ($w_{y\hat{y}}$, ranging from 0.5 to 3.0) is shown in heatmaps. The upper limit for the binomial confidence interval corresponding to *alpha* = 0.05 is 0.065. Values below this threshold (colored white) indicate a valid type I error control.

The simulated predicted values are constructed in a similar fashion, but may depend on *c* as well:

$$\hat{y}|y_i, c_i \sim f_{\delta,\epsilon}(\mathcal{N}(0,1)) + w_{y\hat{y}}\, g(y_i) \;+\; w_{c\hat{y}}\, c_i$$

Note that simulations with $w_{c\hat{y}} = 0$ produce data under the null hypothesis of no confounding bias.

To test the implementation for categorical variables, simulated y, $\hat{y}$ and c variables are binarized by thresholding at 0.

Simulations for comparison with partial Spearman correlation and linear CPT

To demonstrate the need for the proposed GAM-based CPT approach for partial confounder testing (Fig. 3), its validity was contrasted to partial Spearman correlation and the linear variety of CPT (based on eq. 4, as described by [36]) with the following simulation parameters: sample size $n = 1000$, $w_{c\hat{y}} = 0$ (i.e. H0 simulations only), taking all combinations of $w_{yc} \in \{0.5, 1, 2, 3\}$ and $w_{y\hat{y}} \in \{0.5, 1, 2, 3\}$. Furthermore, simulations cases with non-normality ($f_{\delta=0.1,\epsilon=2}$) and non-linearity (sigmoid g) has also been investigated for all simulation cases.

For each parameter combination, 1000 repetitions were performed and false positive rates were calculated as the ratio of p-values smaller than $\alpha = 0.05$.

The simulation cases are exemplified (with $w_{yc} = w_{y\hat{y}} = 2$) on the left of Figure 3.

Simulations for evaluating power.

100 repetitions were performed of all combination of the following parameter values: $w_{yc} \in \{0.5, 1, 2, 3\}$, $w_{y\hat{y}} \in \{0.5, 1, 2, 3\}$, $w_{c\hat{y}} \in \{0, 0.2, 0.4, 0.6\}$, $n \in \{50, 100, 500, 1000\}$. All simulations were performed with both linear and sigmoid dependence as well as with normal and non-normal conditional distributions: $(\delta, \epsilon) = \{(0.1, 2), (1, 0), (1.05, -3), (1.5, -5), (5, -10)\}$.

The partial confounder tests, as implemented in version 0.20.0 of the package *'mlconfound'* was run with default parameters (1000 permutations and 50 Markov-chain Monte-Carlo steps to generate the conditioned permutations) and by implying categorical variables, where needed.

All code used for the simulations is available on github[2].

Application on functional brain connectivity data

The usefulness of the proposed confounder tests is demonstrated by applying them for predictive classification and regression models based on functional brain connectivity data, processed with different confound-mitigation approaches.

Partial confounder testing was performed with 10000 permutations and 50 Markov-chain Monte Carlo steps, as implemented in version 0.20.0 of the package *'mlconfound'*. Unconditional dependence across the involved variables was investigated with conventional permutation tests on the R^2 values, with 1000 permutations.

All empirical analyses are available as jupyter notebooks on

2 https://github.com/pni-lab/mlconfound-manuscript/tree/main/simulated

github[3].

HCP: testing age and acquisition batch bias in fluid intelligence prediction

The Human Connectome Project dataset contains imaging and behavioral data of approximately 1200 healthy subjects [55]. Preprocessed resting state fMRI connectivity data (partial correlation matrices) [56] as published with the HCP1200 release (N=999 participants with functional connectivity data) were used to build models that predict individual fluid intelligence scores (G_f), measured with Penn Progressive Matrices [57].

To ensure normality of the target variable for the partial correlation-based analyses, G_f was non-linearly transformed to normal distribution with the quantile transformation [58] as implemented in *scikit-learn* [59] (see Supplementary Figure S8 for details).

Features (functional connectivities across 100 group-independent component analysis based regions) were either (i) considered in their raw form or were subject to confound mitigation approaches by (ii) feature regression [9] or (iii) COMBAT [28, 60]. The feature mitigation strategies were separately applied for acquisition batch and age group as confounder variable.

Each of the 5 types of features (raw, regressing out acquisition batch, regressing out age group, COMBAT with acquisition batch, COMBAT with age group) was independently incorporated into a scikit-learn-based [59] machine learning procedure aiming to predict the individual fluid intelligence scores with a ridge regression [61]. The α parameter of the ridge model was considered as a hyperparameter ($\alpha \in \{0.00001, 0.0001, 0.001, 0.01, 0.1, 1, 10, 100, 1000, 10000, 100000\}$) and optimized in a nested cross-validation with 10 folds both in the inner and the outer loop and with mean squared error as optimization metric. Confound mitigation was performed inside of the outer cross-validation loop, to avoid leakage.

ABIDE: testing motion- and center-bias in predictive models of autism spectrum disorder diagnosis

The proposed tests were applied to provide evidence of center- and motion-bias in diagnostic predictive models of autism spectrum disorder (ASD), trained on the Autism Brain Imaging Data Exchange (ABIDE) dataset [62] involving 866 participants (ASD: 402, neurotypical control: 464). Preprocessed regional timeseries data was obtained as shared [4] with the by Dadi et al. [63] which was based on preprocessed image data provided by the Preprocessed Connectome Project [64].

Tangent correlation across the timeseries of the n=122 regions of the BASC [65] brain atlas was computed with nilearn[5] [66, 67].

The resulting functional connectivity estimates were considered as features either (i) in their raw form or after applying (ii) feature regression [9] or (iii) COMBAT [28, 60]. The investigated confounder variables were 'imaging center' and 'in-scanner motion', as measured by the mean framewise displacement (FD), as defined by [68]. Mean FD was non-linearly transformed to normal distribution with the quantile transformation [58] as implemented in *scikit-learn* [59] (see Supplementary Figure S9 for details).

As COMBAT is not able to handle continuous variables (since it was primarily designed to remove categorical "batch-effects"), motion was binned into 10 groups, based on equidistant data quantiles ranging from 0 to 1.

The total of five types (raw, feature regression of site, feature regression of motion, COMBAT with site, COMBAT with motion) of features were independently incorporated into a scikit-learn-based [59] machine learning procedure aiming to predict the diagnosis (DX: ASD vs. neurotypical controls) with a L2-regularized logistic regression, as previously recommended [63]. The C parameter of the model was considered as a hyperparameter ($C \in \{0.1, 1, 10\}$) and optimized in a nested cross-validation with 10 folds both in the inner and the outer cv-s and with area under the receiver operator curve (AUC under ROC) as optimization metric. Confound mitigation was performed inside of the outer cross-validation loop, to avoid leakage. Confounder testing was performed on the predicted class probabilities.

Results

The partial confounder tests

The proposed *partial confounder tests* has been implemented in the python package *mlconfound*[6] (biotools:mlconfound, RRID:SCR_022545).

Simulations

Type I error

As suggested by theory (see Methods for details) and shown by the simulations with a wide range of settings, both of the proposed tests provide a valid Type I error control (Fig. 4 and Supplementary Figures S1-3), even in case of non-linearity and non-normality (Figs. 3, 5 and Supplementary Figures S4-7), except when non-normality is extreme (purple distribution on Fig. 5, kurtosis: 42, skewness: -6).

Power

Estimates of statistical power for the partial confounder test (with normal and linear simulations, for a wide range of parameters) were found to be virtually identical to those of Pearson's partial correlation. (see Figure 4 and Supplementary Figure S12). Notably, with sample sizes as large as 1000, a confounder contributing only ~ 4% to the variance of the predictions ($w_{c\hat{y}}$ = 0.2) can already be robustly detected with a power of 94-100%. With a sample size of 500, the same confounding bias is still detected with a power greater than 84-100% in all of the simulation cases. A sample size of 100 requires a somewhat stronger bias with approximately 12% of explained variance ($w_{c\hat{y}}$ = 0.4) to achieve a reasonable level of power (75-98%). Finally, even with a relatively low sample size of 50, the same amount of confounder variance is detected with a power of at least 50%. If the confounder explains more than 25% of variance, it is almost certainly detected even with a low sample size of $n \geq 50$.

Simulations show that non-normality has minimal effect on the power of the tests, except in case of extreme non-normality. (Fig. 5). Simulations with sigmoid dependence resulted in an apparent loss of statistical power, however this is simply a consequence of the simulation methodology: with the same parameters, the sigmoid transformed confounder explains only approximately half the variance as compared to linear simulations. Type I error control was valid in case of categorical variables, as well (Supplementary Figures 1,3,5,7).

Neuroimaging data

To demonstrate the usefulness of the proposed tests in detecting various types of confounding bias, they have been deployed in two typical research scenarios - a regression and a classification problem - where confounder effects are known to hamper the development of biomedically useful predictive models. The empirical anal-

3 https://github.com/pni-lab/mlconfound-manuscript/tree/main/empirical
4 https://osf.io/hc4md
5 http://nilearn.github.io/
6 https://mlconfound.readthedocs.io

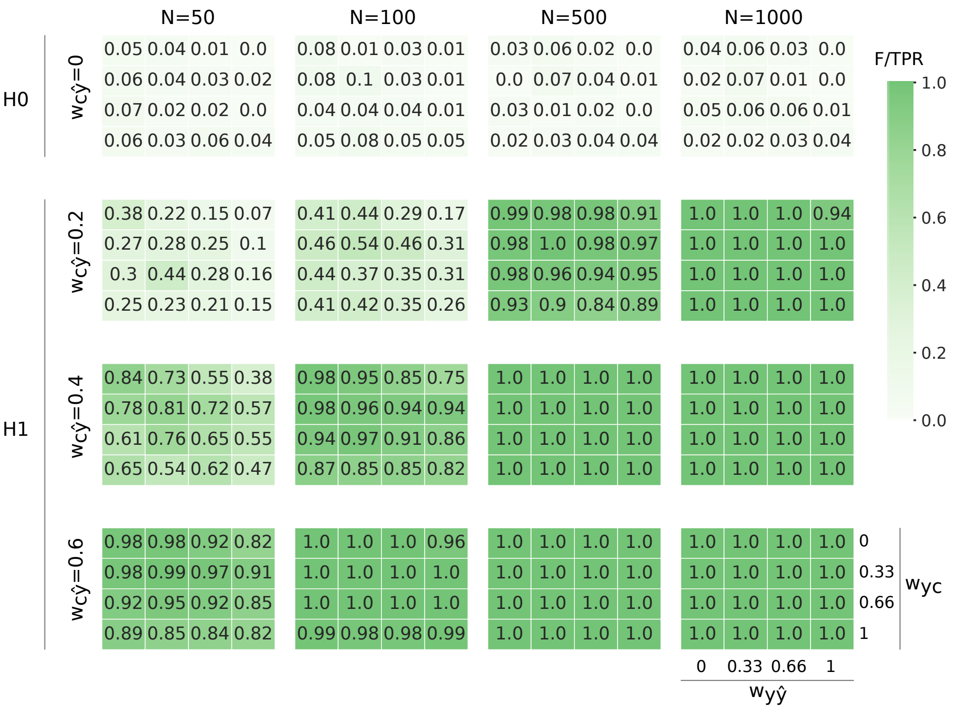

Figure 4. The partial confounder test provides a strict control for Type I errors and a high statistical power in simulated data.
Heatmaps depict positive rates (ratio of p-values lower than 0.05, color coded as shown by the palette on the right) in various simulations settings (100 simulations per tile) with different simulation weights $w_{y\hat{y}}$ (predictive performance; horizontal axis on each heatmap), w_{yc} (confounder-target association; vertical axis on each heatmap), $w_{c\hat{y}}$ (degree of confounder bias; rows) and for different sample sizes (N, columns). Weights 0.2, 0.33, 0.4, 0.6, 0.66, 1.0 can be assigned to the following approximate explained variance values: 4%, 10%, 12%, 25%, 30%, 50%, respectively. First row contains simulations under the null hypothesis (H0, no confounding bias), rows 2-4 represent simulations from the alternative hypothesis (H1, confounding bias). Positive rates for the simulations under the null and the alternative hypotheses can be interpreted as type I error rate and statistical power, respectively. The higher 95% confidence limit for a positive rate of *alpha* = 0.05 is 0.11 for each tile.

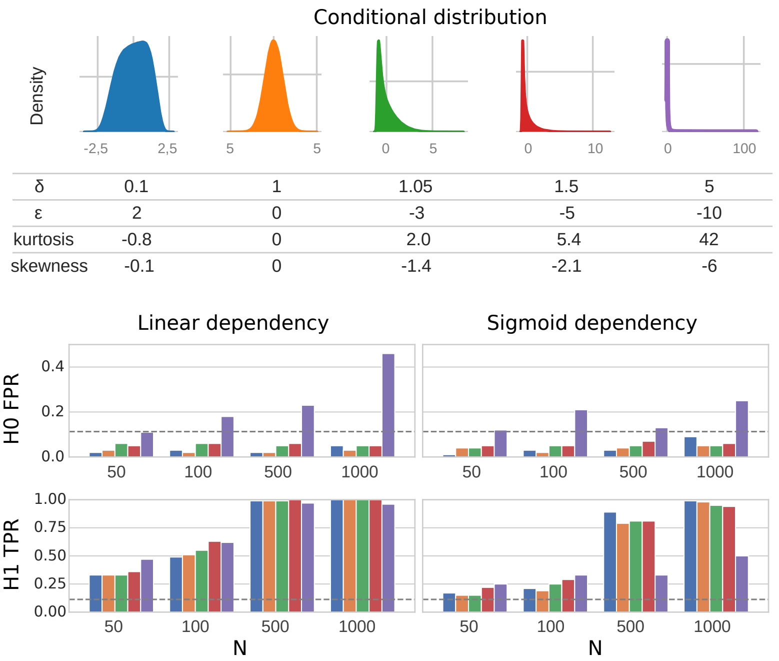

Figure 5. The partial confoudner test is robust to non-normality and non-linearity.
Simulations included variables with five different degrees of non-normality (top panel), as introduced with various δ and ϵ values of the *sinh-arcsinh* transformation (yellow: normally distributed). Fisher's kurtosis and skewness is given for each distribution. False and true positive rates in the simulations under H0 and H1, respectively, for each investigated sample size (N), are depicted by barplots for both linear and sigmoid dependency structure. Upper 95% binomial confidence limit corresponding to *alpha* = 0.05 is shown with a vertical dashed line.

yses confirmed the presence of non-linearity and non-normality in the output of the predictive models (see Supplementary Figure S11 for more details).

HCP dataset

Functional connectivity data from the Human Connectome Project [55] (HCP) was used to build predictive models of fluid intelligence

(G_f) and to test for the previously discussed confounding effect of age [19, 18] and, additionally, the - somewhat underdiscussed - batch-like effect of acquisition date of the data within the course of the data acquisition process.

Both acquisition batch and age group were statistically significantly associated with G_f (R^2 = 0.032 and 0.011 and $p < 0.001$ and $p = 0.001$, respectively, see also Table 2). The model trained on the raw (unadjusted) connectivity features predicted fluid intelligence with a medium effect size ($R^2 = 0.095$, $p < 0.001$).

The partial confounder test revealed that the 'raw' model (without confounder mitigation) was significantly biased both by age group and acquisition batch (both $p < 0.0001$, first column of Fig. 6) with later phases of the acquisition and lower age being associated to larger predicted values.

After applying confound mitigation approaches (feature regression or COMBAT) the partial confounder test did not provide evidence for confounding bias anymore ($p > 0.05$ for all; shown in the second and third columns of Fig. 6), neither for acquisition batch nor for age. Both feature regression and COMBAT increased the predictive performance, with COMBAT providing the overall best performances (R^2 = 0.122 and 0.121 when applied to remove the effect of acquisition and age, respectively).

ABIDE dataset

Functional connectivity data from the ABIDE [62] database was used to investigate the potential motion and center bias (as previously reported e.g. by [13, 14] or [12]) when training models that aim to predict ASD diagnosis.

Imaging center and in-scanner motion (normalized mean framewise displacement) were statistically significantly associated with ASD diagnosis (R^2 = 0.019 and 0.028, respectively, $p < 0.001$ for both, see also Table 2). The model trained on the raw (unadjusted) connectivity features predicted diagnosis with a medium effect size ($R^2 = 0.126$, $ROCAUC = 0.71$, $p < 0.001$).

The partial confounder test revealed that the raw model was significantly biased both for age group and acquisition batch (both $p < 0.0001$, see first column on Fig. 7). Predictions for several sites (e.g. Carnegine Mellon University, University of Leuven, Social Brain Lab UMC Groningen) were severely miscalibrated and higher motion was associated to a higher probability for ASD diagnosis.

Both feature regression and COMBAT seemed to significantly attenuate center bias, however, with COMBAT, the partial confounder test still provided evidence for a significant residual bias (0.009, third columns of the first row on Fig 7).

When trying to mitigate the effect of in-scanner motion (bottom row on Fig 7), both confounder mitigation approaches seemed to effectively mitigate motion-bias, as suggested by the partial confounder test ($p > 0.05$, middle and right panel in the bottom row of Fig 7).

Both feature regression and COMBAT considerably improved the predictive performance when mitigating center-effects (AUC = 0.71 without correction and 0.75 with both feature regression and combat). With both feature regression and COMBAT, however, the effort of mitigating motion effects happened at the cost of a drop in predictive performance (AUC = 0.69 and 0.70, for feature regression and COMBAT, respectively)

Discussion

The concept of conditional independence provides a straightforward framework for assessing confounding bias in predictive models , assuming that both the target variable and the potential confounder has been observed for the validation dataset. However, handling the non-normal and/or non-linear conditional dependencies often seen in predictive models [37, 38] (Supplementary Figures S10-11)

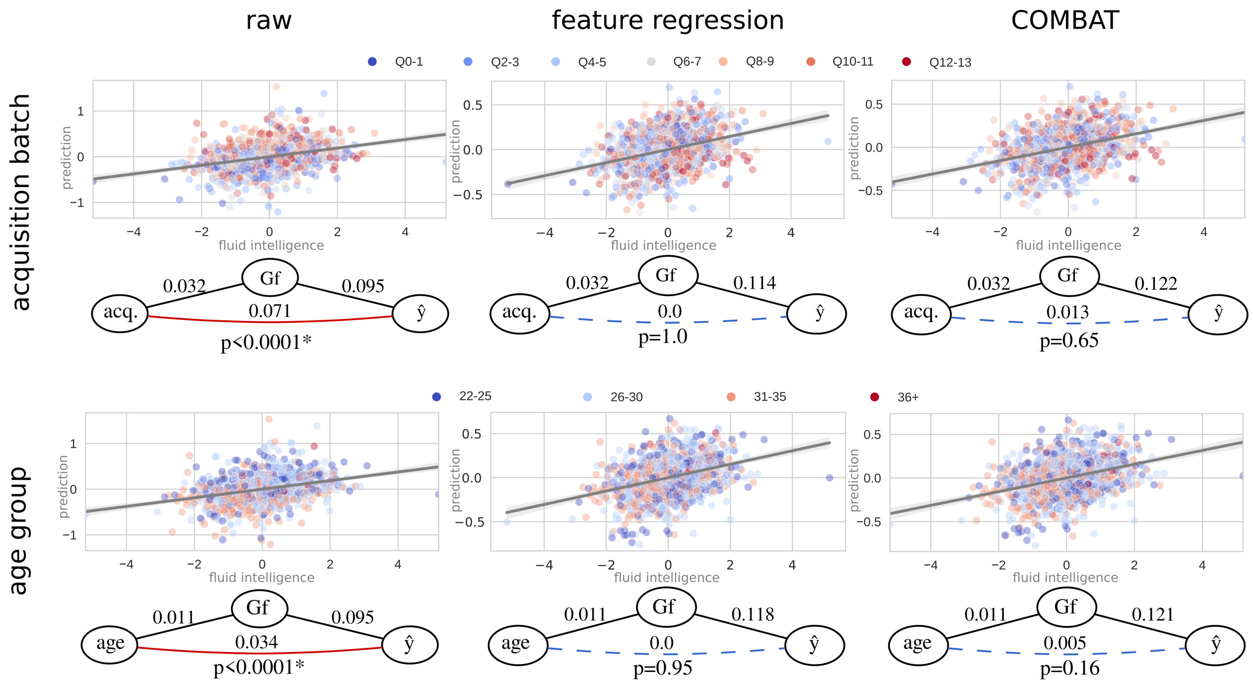

Figure 6. The partial confoudner test reveals that acquisition batch- and age-bias in predictive models of fluid intelligence can be effectively attenuated by confounder mitigation approaches in the HCP dataset.
Scatter plots and regression lines (with 95% confidence intervals) show the association of the observed (horizontals axis) and predicted (vertical axis) fluid intelligence scores with various confound regression strategies. Color-coding of the confounder variables (top: acquisition batch, bottom: age group, as shown by the corresponding legends) reveals confounding bias both for acquisition and age in the models trained on the raw data. This bias is robustly detected by the partial confounder test ($p < 0.0001$) and seems to be effectively mitigated by both feature regression and COMBAT. Relation between the observed (Gf) and predicted ($\hat{y}$) intelligence scores and the confounder variables is given on the graphs via R^2*values*. Both confound mitigation techniques, but especially COMBAT, improve the predictive performance. Solid red line between the confounder and the prediction means significant confounding bias, whereas blue dashed line denotes that confounder testing provided no evidence for bias. P-values are determined with the partial confounder test.

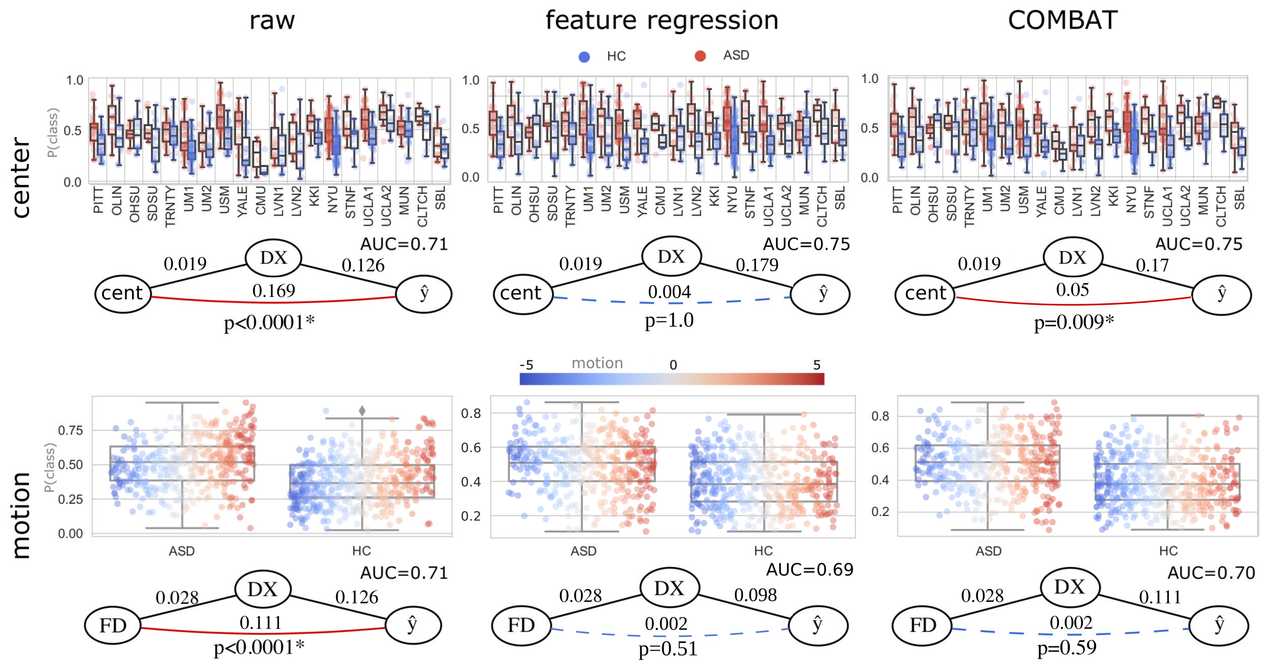

Figure 7. The partial confoudner test identifies an efficient mitigation strategy for motion-bias in predictive models of autism spectrum disorder and reveals residual center-bias after COMBAT in the ABIDE dataset.
Boxplots and points show the predicted class probabilities (0: HC, 1: ASD), separately for the HC and ASD groups. In the top panel, predictions are plotted for each center separately. Color indicates the true diagnosis (DX). AT the bottom plot, color indicates the normalized index of in-scanner motion (normalized FD). The proposed confounder test reveals significant center and motion bias in the model trained on the raw data (p<0.0001). While both motion and center bias was effectively mitigated by both feature regression and COMBAT, the proposed partial confounder test revealed COMBAT was not able to fully remove center bias and resulted in significant "residual bias" (p<0.05). Relation between the true ($\hat{y}$) and predicted diagnosis scores and the confounder variables is shown by the graphs as R^2 values. Solid red line between the confounder and the prediction means significant confounding bias, whereas blue dashed line denotes that confounder testing provided no evidence for bias. P-values are determined with the partial confounder test.

dataset	conf.	method	$R^2_{y,c}$	$p_{y,c}$	$R^2_{\hat{y},c}$	$p_{\hat{y},c}$	$R^2_{\hat{y},y}$	$p_{\hat{y},y}$	partial confounder test
HCP	acq.	raw	0.032	<0.001	0.071	<0.001	0.095	<0.001	**<0.0001**
		f.reg.			0.0	1.0	0.114	<0.001	1.0
		COMBAT			0.013	0.4	0.122	<0.001	0.65
	age	raw	0.011	0.001	0.034	<0.001	0.095	<0.001	**<0.0001**
		f.reg.			0.0	0.92	0.118	<0.001	0.95
		COMBAT			0.005	0.048	0.121	<0.001	0.16
ABIDE	center	raw	0.019	<0.001	0.169	<0.001	0.126	<0.001	**<0.0001**
		f.reg.			0.004	1.0	0.179	<0.001	1.0
		COMBAT			0.05	0.001	0.17	<0.001	**0.009**
	motion	raw	0.028	<0.001	0.111	<0.001	0.126	<0.001	**<0.0001**
		f.reg.			0.002	0.16	0.098	<0.001	0.51
		COMBAT			0.002	0.19	0.111	<0.001	0.59

Table 2. Coefficients-of-determination (R^2), the corresponding p-values and the p-values of the partial confounder tests, for all investigated datasets, confounders (conf.) and confounder-mitigation methods (method). Bold numbers denote significant confounding bias identified by the partial confounder test.

poses a great challenge. In fact, as recently shown by Shah and Peters in their 'no free lunch' theorem [35], it is effectively impossible to establish a *fully non-parametric* conditional independence test with a valid type I error control and a non-trivial power. Indeed, perhaps somewhat surprisingly, but not totally unexpectedly [31] - partial correlation-like analogs of widely used bivariate non-parametric test, like partial Spearman correlation, exhibit inflated type I errors even with slight violations of normality and/or linearity (as clearly demonstrated with simulated data on Fig. 3). While the magnitude of this problem may not be fully appreciated in case of predictive model diagnostics, such tests are, in general, poor choices for testing confounding bias in machine learning. Conditional independence-based confounding bias testing must, therefore, be designed so that its suitability for the particular problem may be judged easily.

These tests place no assumptions on the conditional distributions of the model output, ensuring valid model diagnostics even in cases of non-normally and non-linearly dependent predictions. This property distinguishes the approach from other alternatives as it guarantees a valid type-I error control even in cases of non-normally and non-linearly dependent predictions, i.e. in cases where Pearson and Spearman partial correlations, and many other methods fail.

The proposed tests are based on solid theoretical foundations, underpinned by mathematical proofs. The main purpose of the simulated and empirical experiments was, therefore, not to justify the validity of the approach but to (i) test the software implementation, (ii) estimate statistical power in various situations and (ii) exemplify how the partial confounder test can be used with real experimental data. The validity of the type I error control and was confirmed by our simulations, even if both the predictions and the confounder are non-normally and/or non-linearly dependent on the target variable (except by extreme non-normality). While different biomedical applications may consider different amounts of bias to be relevant, in most cases it is possible to set an upper bound for confounding bias that is still tolerable in certain applications. The simulation results can serve as a basis for power calculations in these cases, in order to identify the necessary sample size for proper model diagnostics.

A characteristic example for the potential areas of applications is the novel field of population neuroscience, where applying predictive modelling and machine learning on large-scale functional neuroimaging data holds great potential for both revolutionizing our understanding of the physical basis of mind and delivering clinically useful tools for diagnostics or therapeutic decision making [5, 23, 3, 25]. However, the presence of confounders that are typical for biomedical research (e.g. sample demographics, center-effects) or specific to the data acquisition and processing approach (e.g. imaging artifacts) presents a great challenge to these effort [29]. The usefulness of the proposed tests is demonstrated in two such examples, using the HCP [55] and the ABIDE [62] datasets.

In case of the HCP dataset, the statistically significant age bias of the 'raw' model for predicting fluid intelligence is in line with previous findings [18, 19] and could likely exaggerate to a serious bias when testing the model on data of participants outside of the - relatively narrow - age range of the HCP sample. In this case, the bias would likely significantly harm the out-of-sample generalizability of this model. The bias of the same model for acquisition batch can also be problematic, especially as it has not yet been thoroughly discussed in case of the HCP dataset. There can be manifold reasons for the observed acquisition bias. Fluid intelligence of the included participants might be, for instance, affected by a changing selection bias during participant recruitment (e.g. as a consequence of the human connectome project receiving an increasing degree of public interest during its course).

In the ABIDE dataset, neither the center bias nor the age bias is surprising in the case of the 'raw' model but both would be obviously severely problematic for a diagnostic biomarker candidate of ASD. For instance the model trained on the raw (unadjusted) features - depending on the calibration of the predicted class probabilities - might classify all participants form e.g. the CMU (Carnegie Mellon University) center as neurotypical control participants. Similarly, the models biased by motion - next to having questionable neuroscientific validity - might systematically fail in populations with a tendency for higher in-scanner motion (as known for many conditions, among others ADHD [10] or Alzheimer's disease [9]).

The partial confounder test provided quantitative, statistically rigorous metrics for assessing the effectiveness of the investigated confounder mitigation techniques. In the HCP data, it revealed that both the acquisition bias and the age bias was very effectively removed by both feature regression and COMBAT (p>0.05 for all). Given the high power of the test at the sample sizes of the HCP dataset (N = 999), any remaining confounding bias is most probably very safely negligible and well out of the range of practical relevance.

The confound mitigation approaches performed well in attenuating motion bias in the ABIDE dataset, as well, as no residual bias was detected by the proposed test. However, the success of COMBAT in eliminating motion bias is not to be taken without any objections. As COMBAT was originally developed for harmonizing effects of categorical variables (e.g. center or batch), its application for continuous confounder variables is not trivial. Inputting discretized versions of continuous variables into COMBAT might be sub-optimal and raises further questions e.g regarding the optimal number of bins used during the discretization.

Importantly, the partial confounder test revealed, that the center bias of the classification in the massively multi-center ABIDE dataset was, although mitigated, but not successfully removed by COMBAT. While determining the relevance of the remaining bias is out of the scope of this paper, the example demonstrates the need for checking confounder bias even if state-of-the-art confounder mitigation approaches have been applied. If the proposed test provides evidence for residual confounding bias, the researcher might consider the use of another mitigation approach (e.g. feature regression in the given case) or the evaluation of confound-free performance e.g. via 'confound-isolating cross-validation' [29].

In sum, the application of the partial confounder test on the real data examples suggests that confounding bias must be always carefully investigated and reported in studies utilizing predictive modelling and machine learning as (i) variables as trivial as the date of the acquisition can cause significant confounding bias and (ii) in certain situations, state-of-the-art confoudner mitigation techniques may not provide sufficient mitigation of confounding bias and (iii) unnecessary confounder correction may eliminate variance-of-interest. As the proposed test is a model-agnostic post-hoc test, it can be used to benchmark different machine learning models and to further characterize already trained models in external validation samples, where a larger set of potential confounder variables is available (Supplementary Figure 13). The partial confounder test can be considered as a useful, objective benchmark to guide the search for a suitable confounder mitigation approach for every dataset.

Conclusion

The lack of rigorous statistical tests for confounding bias significantly hampers the development of predictive models in many fields of research, including population neuroscience, where handling confounding effects is especially challenging [23].

To fill this critical gap in predictive model development, here I proposed two novel tests, the *partial* and the *full confounder tests*, which probe the null hypotheses of 'no confounding bias' and 'full confounding bias', respectively. The tests are distinguished from alternative approaches by their robustness to non-normally and non-linearly dependent predictions, rendering them applicable

with a wide variety of machine learning models. The tests have, moreover, a minimal computational overhead, as re-fitting the model is not required.

As demonstrated on functional brain connectivity-based predictive models of fluid intelligence and autism spectrum disorder, the tests can guide the optimization of confound mitigation strategies and allow quantitative statistical assessment of the robustness, generalizability and neurobiological validity of predictive models in biomedical research. Given their simplicity, robustness, wide applicability, high statistical power and computationally effective implementation (available in the python package *mlconfound*[7]), the partial and full confounder tests emerge as novel tools in the methodological arsenal of predictive modelling and may largely accelerate the development of clinically useful machine learning biomarkers.

Declarations

Data Availability

Empirical analysis was based on preprocessed data provided by the Human Connectome Project, WU-Minn Consortium [55] (principal investigators: D. Van Essen and K. Ugurbil; 1U54MH091657) funded by the 16 NIH institutes and centers that support the NIH Blueprint for Neuroscience Research; and by the McDonnell Center for Systems Neuroscience at Washington University and the Autism Brain Imaging Data Exchange (ABIDE) consortium [62].

All data used in the present study are available for download from the Human Connectome Project (www.humanconnectome.org). Users must agree to data use terms for the HCP before being allowed access to the data and ConnectomeDB; details are provided at https://www.humanconnectome.org/study/hcp-young-adult/data-use-terms. Python implementation of the 'mlconfound' package is available on github. All analysis code is available at github and via the GigaScience database GigaDB [69].

Funding

This research was supported by the Deutsche Forschungsgemeinschaft (DFG, German Research Foundation) – Projektnummer 316803389 – SFB 1280 and TRR 289 Treatment Expectation - Projektnummer 422744262.

Competing Interests

The authors declare that they have no competing interests.

Acknowledgement

I am thankful to Ulrike Bingel (University Hospital Essen, Germany) and Robert Englert (University Hospital Essen, Germany) for their valuable insights and comments on the manuscript. I show appreciation to the contributors of Human Connectome Project and the Autism Brain Imaging Exchange study for collecting and sharing the quality data to researchers.

List of abbreviations

- ABIDE: Autism Brain Imaging Data Exchange
- ASD: Autism Spectrum Disorder
- AUC: Area under the curve
- COMBAT: "Combatting batch effects" data harmonization approach
- CPT: conditional permutation testing
- DX: diagnosis
- FD: framewise displacement
- GAM: generalized additive model
- Gf: fluid intelligence
- HCP: Human Connectome Project
- MCMC: Markov-chain Monte-Carlo
- ROC: receiver operator curve

7 https://mlconfound.readthedocs.io

References

1. Vogt N. Machine learning in neuroscience. Nature Methods 2018;15(1):33–33.
2. Kent DM, Steyerberg E, van Klaveren D. Personalized evidence based medicine: predictive approaches to heterogeneous treatment effects. Bmj 2018;363.
3. Spisak T, Kincses B, Schlitt F, Zunhammer M, Schmidt-Wilcke T, Kincses ZT, et al. Pain-free resting-state functional brain connectivity predicts individual pain sensitivity. Nature communications 2020;11(1):1–12.
4. Walsh I, Fishman D, Garcia-Gasulla D, Titma T, Pollastri G, Harrow J, et al. DOME: recommendations for supervised machine learning validation in biology. Nature methods 2021;p. 1–6.
5. Woo CW, Chang LJ, Lindquist MA, Wager TD. Building better biomarkers: brain models in translational neuroimaging. Nature neuroscience 2017;20(3):365–377.
6. Obermeyer Z, Powers B, Vogeli C, Mullainathan S. Dissecting racial bias in an algorithm used to manage the health of populations. Science 2019;366(6464):447–453.
7. Mehrabi N, Morstatter F, Saxena N, Lerman K, Galstyan A. A survey on bias and fairness in machine learning. ACM Computing Surveys (CSUR) 2021;54(6):1–35.
8. Prosperi M, Guo Y, Sperrin M, Koopman JS, Min JS, He X, et al. Causal inference and counterfactual prediction in machine learning for actionable healthcare. Nature Machine Intelligence 2020;2(7):369–375.
9. Rao A, Monteiro JM, Mourao-Miranda J, Initiative AD, et al. Predictive modelling using neuroimaging data in the presence of confounds. NeuroImage 2017;150:23–49.
10. Eloyan A, Muschelli J, Nebel MB, Liu H, Han F, Zhao T, et al. Automated diagnoses of attention deficit hyperactive disorder using magnetic resonance imaging. Frontiers in systems neuroscience 2012;6:61.
11. Couvy-Duchesne B, Ebejer JL, Gillespie NA, Duffy DL, Hickie IB, Thompson PM, et al. Head motion and inattention/hyperactivity share common genetic influences: implications for fMRI studies of ADHD. PloS one 2016;11(1):e0146271.
12. Gotts SJ, Saad ZS, Jo HJ, Wallace GL, Cox RW, Martin A. The perils of global signal regression for group comparisons: a case study of Autism Spectrum Disorders. Frontiers in human neuroscience 2013;7:356.
13. Spisak T, Jakab A, Kis SA, Opposits G, Aranyi C, Berenyi E, et al. Voxel-wise motion artifacts in population-level whole-brain connectivity analysis of resting-state FMRI. PloS one 2014;9(9):e104947.
14. Spisak T, Kincses B, Bingel U. Optimal choice of parameters in functional connectome-based predictive modelling might be biased by motion: comment on Dadi et al. bioRxiv 2019;p. 710731.
15. Orban C, Kong R, Li J, Chee MW, Yeo BT. Time of day is associated with paradoxical reductions in global signal fluctuation and functional connectivity. PLoS biology 2020;18(2):e3000602.
16. Cole MW, Yarkoni T, Repovš G, Anticevic A, Braver TS. Global

connectivity of prefrontal cortex predicts cognitive control and intelligence. Journal of Neuroscience 2012;32(26):8988–8999.
17. He T, Kong R, Holmes AJ, Nguyen M, Sabuncu MR, Eickhoff SB, et al. Deep neural networks and kernel regression achieve comparable accuracies for functional connectivity prediction of behavior and demographics. NeuroImage 2020;206:116276.
18. Dubois J, Galdi P, Paul LK, Adolphs R. A distributed brain network predicts general intelligence from resting-state human neuroimaging data. Philosophical Transactions of the Royal Society B: Biological Sciences 2018;373(1756):20170284.
19. Lohmann G, Lacosse E, Ethofer T, Kumar VJ, Scheffler K, Jost J. Predicting intelligence from fMRI data of the human brain in a few minutes of scan time. bioRxiv 2021;.
20. Lwowski B, Rios A. The risk of racial bias while tracking influenza-related content on social media using machine learning. Journal of the American Medical Informatics Association 2021;28(4):839–849.
21. Li J, Bzdok D, Holmes A, Yeo T, Genon S. Not one model fits all: unfairness in RSFC-based prediction of behavioral data in African American. Helmholtz AI kick-off meeting 2020;.
22. Paulus MP, Thompson WK. Computational approaches and machine learning for individual-level treatment predictions. Psychopharmacology 2021;238(5):1231–1239.
23. Smith SM, Nichols TE. Statistical challenges in "big data" human neuroimaging. Neuron 2018;97(2):263–268.
24. Wachinger C, Rieckmann A, Pölsterl S, Initiative ADN, et al. Detect and correct bias in multi-site neuroimaging datasets. Medical Image Analysis 2021;67:101879.
25. Nunes A, Schnack HG, Ching CR, Agartz I, Akudjedu TN, Alda M, et al. Using structural MRI to identify bipolar disorders–13 site machine learning study in 3020 individuals from the ENIGMA Bipolar Disorders Working Group. Molecular psychiatry 2020;25(9):2130–2143.
26. Dukart J, Schroeter ML, Mueller K, Initiative ADN. Age correction in dementia–matching to a healthy brain. PloS one 2011;6(7):e22193.
27. Abdulkadir A, Ronneberger O, Tabrizi SJ, Klöppel S. Reduction of confounding effects with voxel-wise Gaussian process regression in structural MRI. In: 2014 International Workshop on Pattern Recognition in Neuroimaging IEEE; 2014. p. 1–4.
28. Johnson WE, Li C, Rabinovic A. Adjusting batch effects in microarray expression data using empirical Bayes methods. Biostatistics 2007;8(1):118–127.
29. Chyzhyk D, Varoquaux G, Milham M, Thirion B. How to remove or control confounds in predictive models, with applications to brain biomarkers. GigaScience 2022;11.
30. Dockès J, Varoquaux G, Poline JB. Preventing dataset shift from breaking machine-learning biomarkers. GigaScience 2021;10(9):giab055.
31. Korn EL. The ranges of limiting values of some partial correlations under conditional independence. The American Statistician 1984;38(1):61–62.
32. Bergsma W. Nonparametric testing of conditional independence by means of the partial copula. Available at SSRN 1702981 2010;.
33. Candès E, Fan Y, Janson L, Lv J. Panning for gold: Model-X knockoffs for high-dimensional controlled variable selection. arXiv preprint arXiv:161002351 2016;.
34. Peters J, Bühlmann P, Meinshausen N. Causal inference by using invariant prediction: identification and confidence intervals. Journal of the Royal Statistical Society Series B (Statistical Methodology) 2016;p. 947–1012.
35. Shah RD, Peters J. The hardness of conditional independence testing and the generalised covariance measure. The Annals of Statistics 2020;48(3):1514–1538.
36. Berrett TB, Wang Y, Barber RF, Samworth RJ. The conditional permutation test for independence while controlling for confounders. Journal of the Royal Statistical Society: Series B (Statistical Methodology) 2020;82(1):175–197.
37. García S, Fernández A, Luengo J, Herrera F. A study of statistical techniques and performance measures for genetics-based machine learning: accuracy and interpretability. Soft Computing 2009;13(10):959.
38. Kristensen SB, Sandberg K. Is whole-brain functional connectivity a neuromarker of sustained attention? Comment on Rosenberg & al.(2016). bioRxiv 2017;p. 216697.
39. Neto CE, Pratap A, Perumal TM, Tummalacherla M, Bot BM, Mangravite L, et al. A permutation approach to assess confounding in machine learning applications for digital health. In: Proceedings of the 25th ACM SIGKDD International Conference on Knowledge Discovery & Data Mining; 2019. p. 54–64.
40. Ferrari E, Retico A, Bacciu D. Measuring the effects of confounders in medical supervised classification problems: the Confounding Index (CI). Artificial intelligence in medicine 2020;103:101804.
41. Southworth LK, Kim SK, Owen AB. Properties of balanced permutations. Journal of Computational Biology 2009;16(4):625–638.
42. Hemerik J, Goeman J. Exact testing with random permutations. Test 2018;27(4):811–825.
43. Dawid AP. Conditional independence in statistical theory. Journal of the Royal Statistical Society: Series B (Methodological) 1979;41(1):1–15.
44. Spirtes P, Glymour CN, Scheines R, Heckerman D. Causation, prediction, and search. MIT press; 2000.
45. Fiedler K, Schott M, Meiser T. What mediation analysis can (not) do. Journal of Experimental Social Psychology 2011;47(6):1231–1236.
46. Pitman EJ. Significance tests which may be applied to samples from any populations. Supplement to the Journal of the Royal Statistical Society 1937;4(1):119–130.
47. Fisher R. The Theory of Confounding in Factorial Experiments in Relation to the Theory of Groups. Contributions to Mathematical Statistics 1942;.
48. Hastie T, Tibshirani R. Generalized additive models: some applications. Journal of the American Statistical Association 1987;82(398):371–386.
49. Bennett B. Multiple Regression Analysis of Binary and Multinomial Variates. Sankhyā: The Indian Journal of Statistics, Series A 1966;p. 301–304.
50. Jones RH. Proability estimation usind a multinominal logistic function. Journal of Statistical Computation and Simulation 1975;3(4):315–329.
51. Chambers M, Dinsmore TW. Advanced analytics methodologies: Driving business value with analytics. Pearson Education; 2014.
52. Servén D, Brummitt C, Abedi H. pyGAM: Generalized Additive Models in Python. Zenodo 2018;.
53. Starkweather J, Moske AK, Multinomial logistic regression; 2011.
54. Jones MC, Pewsey A. Sinh-arcsinh distributions. Biometrika 2009;96(4):761–780.
55. Van Essen DC, Smith SM, Barch DM, Behrens TE, Yacoub E, Ugurbil K, et al. The WU-Minn human connectome project: an overview. Neuroimage 2013;80:62–79.
56. Glasser MF, Sotiropoulos SN, Wilson JA, Coalson TS, Fischl B, Andersson JL, et al. The minimal preprocessing pipelines for the Human Connectome Project. Neuroimage 2013;80:105–124.
57. Duncan J, Seitz RJ, Kolodny J, Bor D, Herzog H, Ahmed A, et al. A neural basis for general intelligence. Science 2000;289(5478):457–460.
58. Beasley TM, Erickson S, Allison DB. Rank-based inverse normal transformations are increasingly used, but are they merited? Behavior genetics 2009;39(5):580–595.
59. Pedregosa F, Varoquaux G, Gramfort A, Michel V, Thirion B,

Grisel O, et al. Scikit-learn: Machine learning in Python. the Journal of machine Learning research 2011;12:2825–2830.
60. Fortin JP, Cullen N, Sheline YI, Taylor WD, Aselcioglu I, Cook PA, et al. Harmonization of cortical thickness measurements across scanners and sites. Neuroimage 2018;167:104–120.
61. Hoerl AE, Kennard RW. Ridge regression: applications to nonorthogonal problems. Technometrics 1970;12(1):69–82.
62. Di Martino A, Yan CG, Li Q, Denio E, Castellanos FX, Alaerts K, et al. The autism brain imaging data exchange: towards a large-scale evaluation of the intrinsic brain architecture in autism. Molecular psychiatry 2014;19(6):659–667.
63. Dadi K, Rahim M, Abraham A, Chyzhyk D, Milham M, Thirion B, et al. Benchmarking functional connectome-based predictive models for resting-state fMRI. NeuroImage 2019;192:115–134.
64. Craddock C, Benhajali Y, Chu C, Chouinard F, Evans A, Jakab A, et al. The neuro bureau preprocessing initiative: open sharing of preprocessed neuroimaging data and derivatives. Frontiers in Neuroinformatics 2013;7.
65. Bellec P, Rosa-Neto P, Lyttelton OC, Benali H, Evans AC. Multi-level bootstrap analysis of stable clusters in resting-state fMRI. Neuroimage 2010;51(3):1126–1139.
66. Huntenburg J, Abraham A, Loula J, Liem F, Dadi K, Varoquaux G. Loading and plotting of cortical surface representations in Nilearn. Research Ideas and Outcomes 2017;3:e12342.
67. Estève L. Big data in practice: the example of nilearn for mining brain imaging data. In: Scipy 2015; 2015. .
68. Power JD, Mitra A, Laumann TO, Snyder AZ, Schlaggar BL, Petersen SE. Methods to detect, characterize, and remove motion artifact in resting state fMRI. Neuroimage 2014;84:320–341.
69. Spisak T. Supporting data for "Statistical quantification of confounding bias in machine learning models". GigaScience Database 2022;http://dx.doi.org/10.5524/102244.

Click here to access/download
Supplementary Material
supplementary_material_rev1.pdf
