# Supplementary material for: Statistical quantification of confounding bias in machine learning models
Source: Gigascience. 2022 Aug 26;11:giac082. doi: 10.1093/gigascience/giac082 (PMC9412867; doi:10.1093/gigascience/giac082)

Statistical quantification of confounding bias in machine learning models

Supplementary material

Tamas Spisak
Institute for Diagnostic and Interventional Radiology and Neuroradiology
University Hospital Essen
Hufelandstrasse 55, 45147 Essen
tamas.spisak@uk-essen.de

July 7, 2022

Supplementary Material
for the manuscript entitled "Statistical quantification of confounding bias in machine learning models"
by Tamas Spisak, 2022.

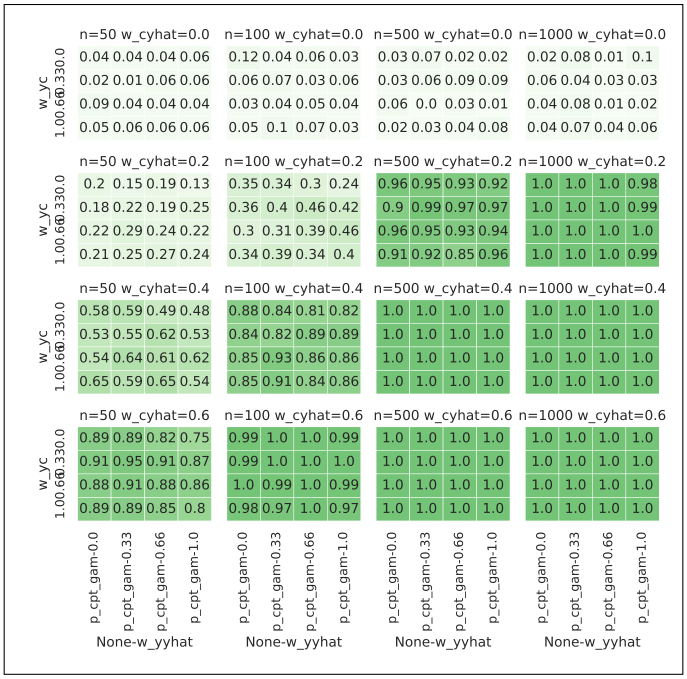

Figure S1: Heatmaps showing the positive rates of the 'partial' confounder test, with categorical variables, normal conditional distribution and linear dependence.

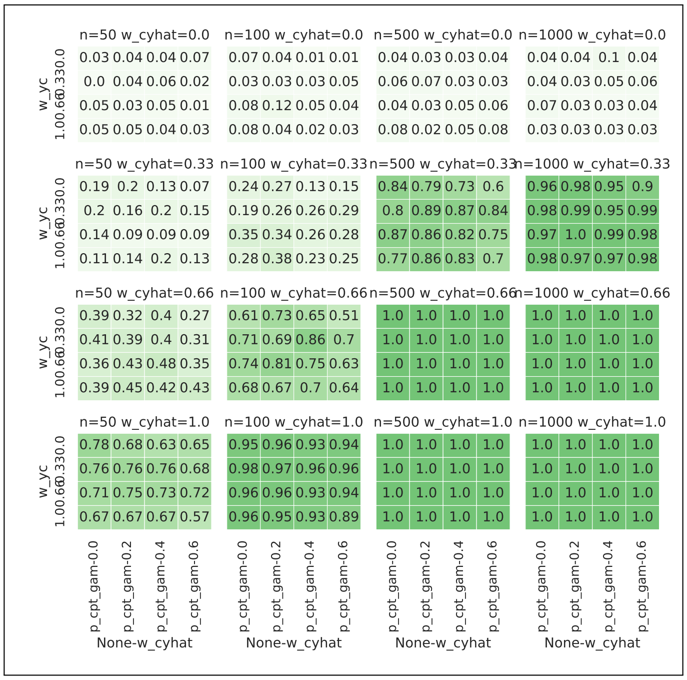

Figure S2: Heatmaps showing the positive rates of the 'full' confounder test, with numerical variables, normal conditional distribution and linear dependence.

Supplementary Material
for the manuscript entitled "Statistical quantification of confounding bias in machine learning models"
by Tamas Spisak, 2022.

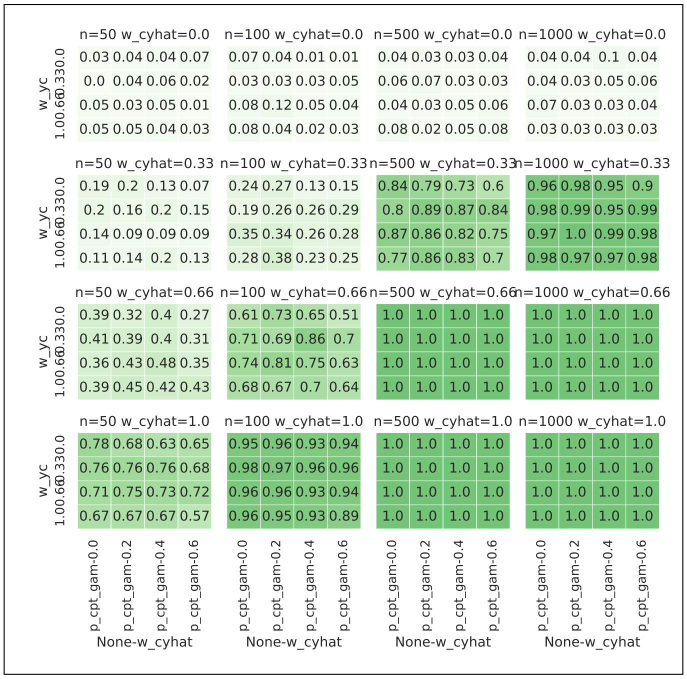

Figure S3: Heatmaps showing the positive rates of the 'full' confounder test, with categorical variables, normal conditional distribution and linear dependence.

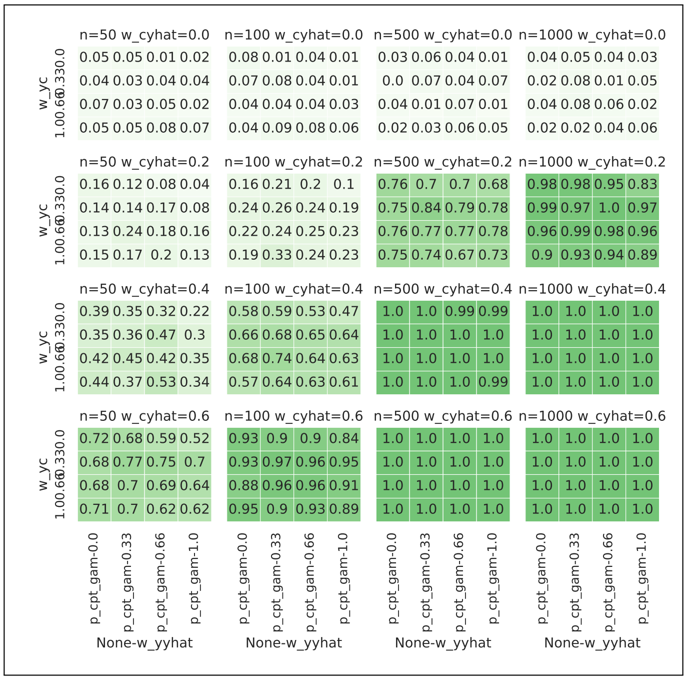

Figure S4: Heatmaps showing the positive rates of the 'partial' confounder test, with numerical variables, normal conditional distribution and sigmoid dependence.

Supplementary Material
for the manuscript entitled "Statistical quantification of confounding bias in machine learning models"
by Tamas Spisak, 2022.

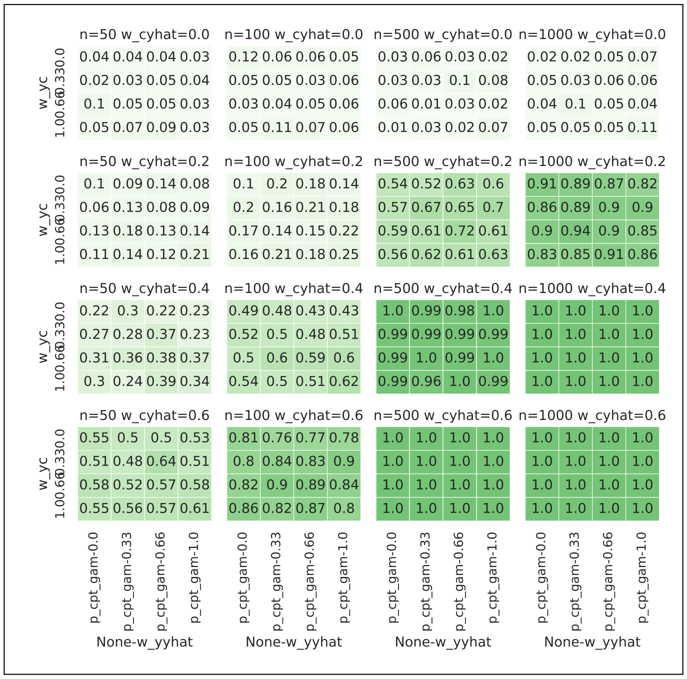

Figure S5: Heatmaps showing the positive rates of the 'partial' confounder test, with categorical variables, normal conditional distribution and sigmoid dependence.

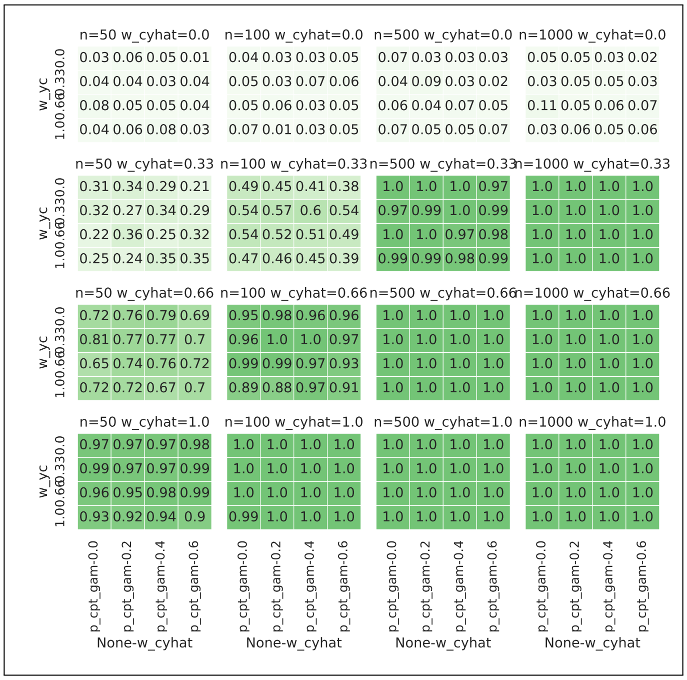

Figure S6: Heatmaps showing the positive rates of the 'full' confounder test, with numerical variables, normal conditional distribution and sigmoid dependence.

Supplementary Material
for the manuscript entitled "Statistical quantification of confounding bias in machine learning models"
by Tamas Spisak, 2022.

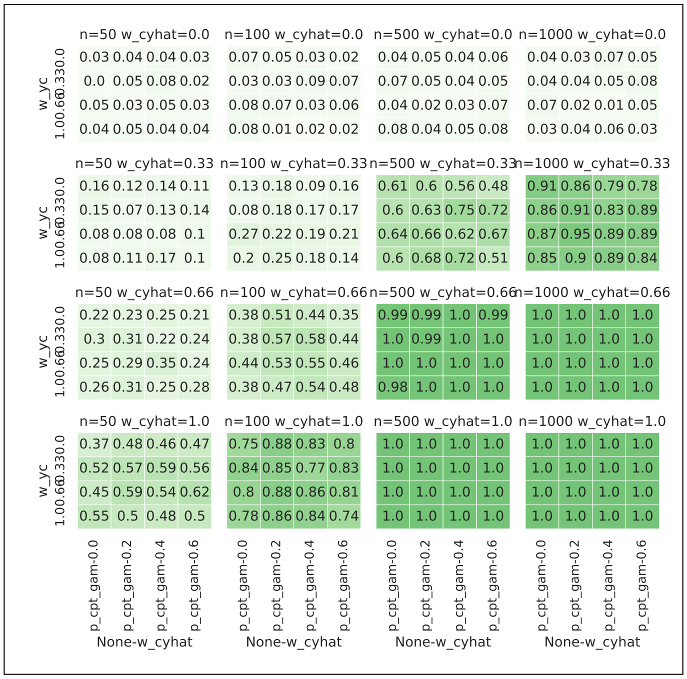

Figure S7: Heatmaps showing the positive rates of the 'full' confounder test, with categorical variables, normal conditional distribution and sigmoid dependence.

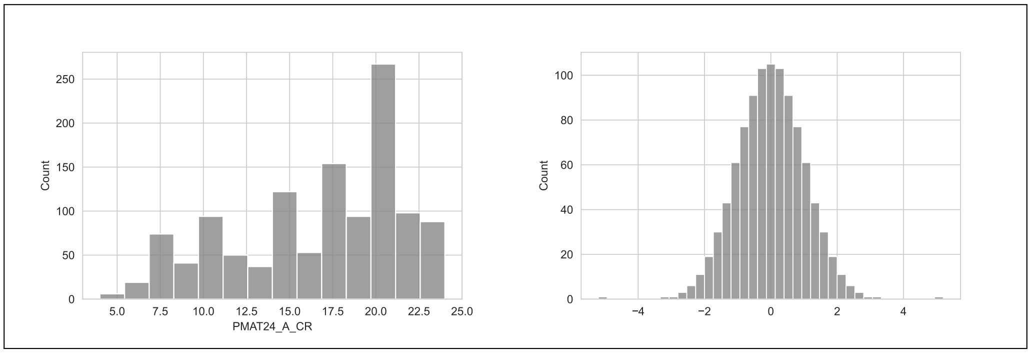

Figure S8: Histogram of fluid intelligence score in the HPC dataset, before (left) and after (right) quantile transformation.

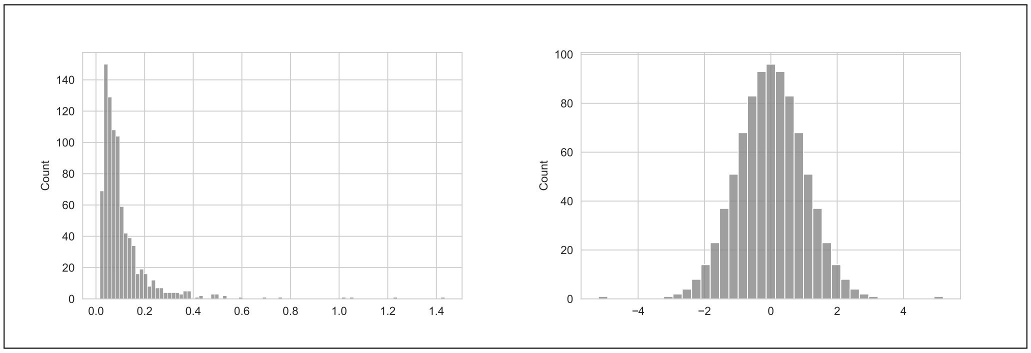

Figure S9: Histogram of mean framewise displacement in the ABIDE dataset, before (left) and after (right) quantile transformation.

Supplementary Material
for the manuscript entitled "Statistical quantification of confounding bias in machine learning models"
by Tamas Spisak, 2022.

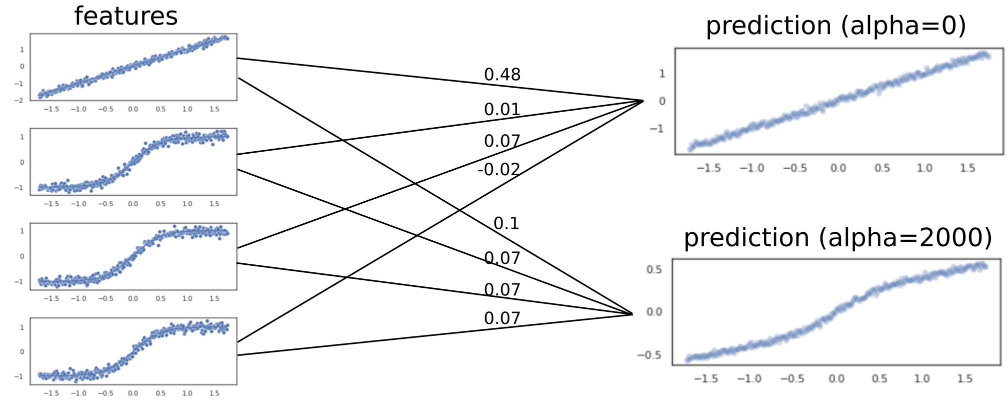

Figure S10: ***Example of non-linearity of model predictions as a consequence of regularization.*** *The same 4 (simulated) features may result in non-linear predictions as the regularization (alpha) of the Ridge model is increased. Model coefficients are shown above the lines connecting the features and the prediction. The full analysis is available at: https://github.com/pni-lab/mlconfound-manuscript/blob/main/simulated/normality*$_a$*nd*$_l$*inearity*$_v$*iolation.ipynb*

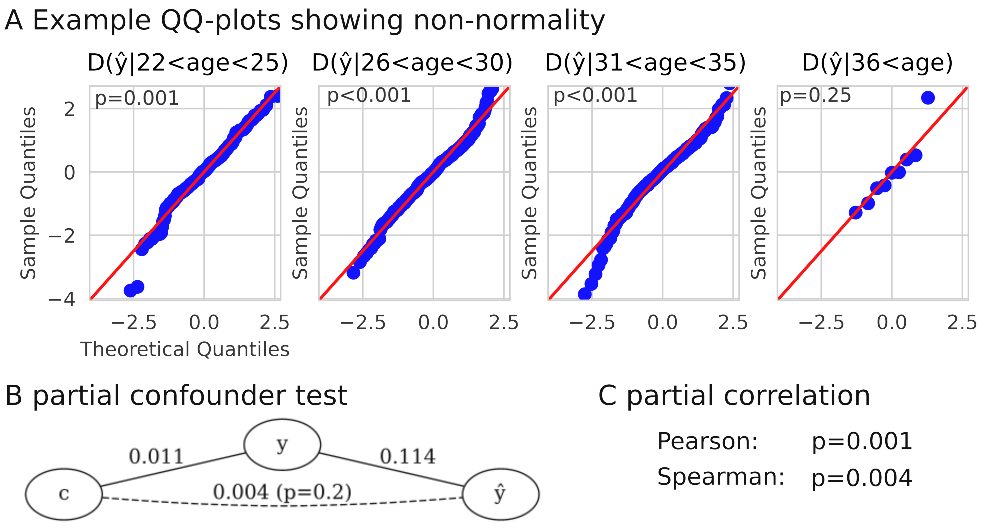

Figure S11: ***Example of non-normality of the conditional distributions*** $\hat{y}|y$ ***and*** $\hat{y}|c$.
(A) Example from the analysis of the HCP dataset, as presented in the previous version of the manuscript. (B) No evidence of confounder bias with the partial confounder test. (C) Presumably false positive observations by Pearson's and Spearman's partial correlations, due to invalid p-values with non-normal conditional distributions. Prediction target: age. Confounder: age. Confound mitigation: age regression. Non-normality was frequently observed in the other cases, as well. The full analysis is available at: https://github.com/pni-lab/mlconfound-manuscript/blob/main/empirical/supplement/check_assumptions.ipynb

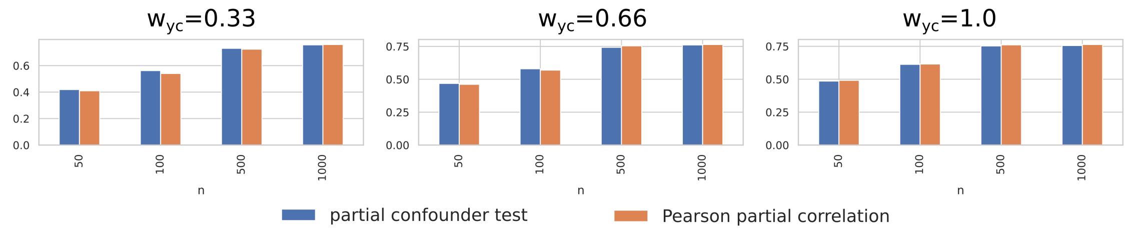

Figure S12: ***In case of linearity and normality, the power of the proposed test is virtually equal to that of Pearson's partial correlation.***
Blue: partial confoudner test; orange: Pearson's partial correlation. Boxplots are based on the simulation cases from Figure 4 of the manuscript.

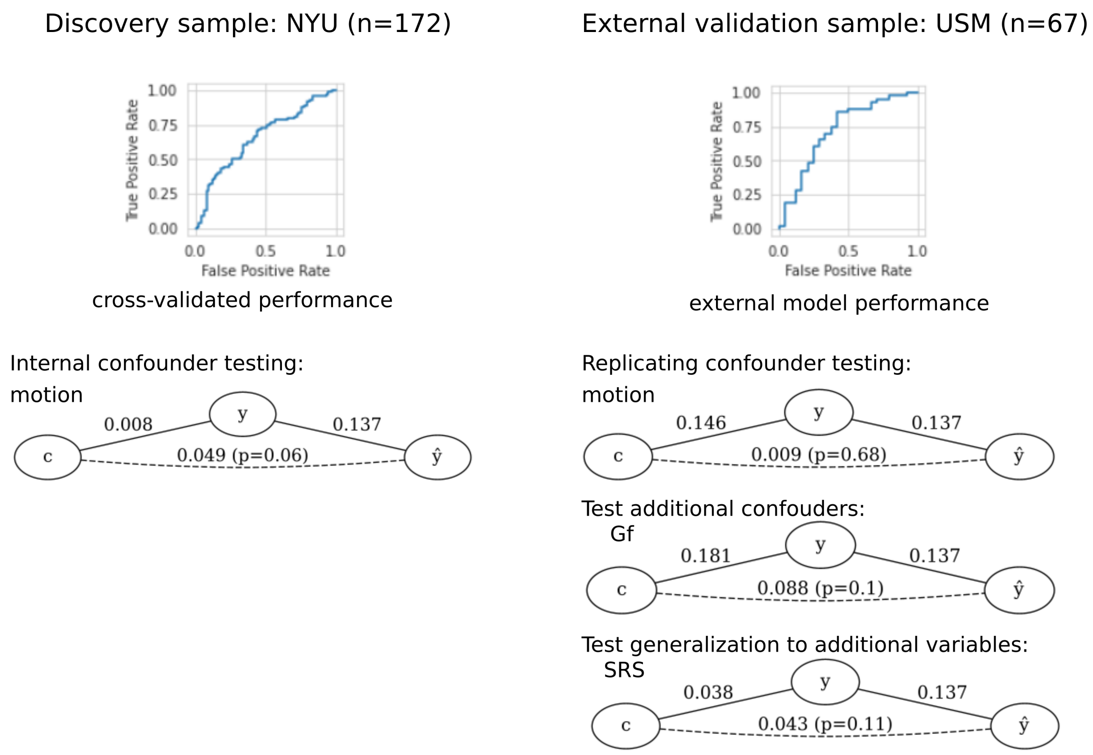

Figure S13: ***The partial confounder test can be used at any phase of model validation.***
The NYU site from the ABIDE dataset has been used as "discovery sample", to train a model predicting ASD diagnosis. The partial confounder test found no evidence for motion bias. The finalized model has been externally validated in data from the USM site. Next to the repeated testing of motion bias, the proposed test is used here for testing another potential confounder (fluid intelligence, Gf) and, additionally, to test if the model generalizes to the SRS (Social Response Scale). Source code available: https://github.com/pni-lab/mlconfound-manuscript/blob/main/empirical/supplement/external_validation.ipynb

Supplementary Analyses

April 22, 2022

1 Supplementary Analysis Notebook 1: Benchmarking Causal Forests with the partial confounder test

Also available on github.

https://github.com/pni-lab/mlconfound-manuscript/blob/main/empirical/supplement/causal_forest.ipynb

[2]:
```
#!pip install causalml econml
import numpy as np
from causalml.dataset import simulate_nuisance_and_easy_treatment
import pandas as pd
from sklearn.model_selection import train_test_split
from sklearn.tree import DecisionTreeRegressor
from  econml.grf import CausalForest
from sklearn.model_selection import KFold
from sklearn.preprocessing import quantile_transform
from sklearn.metrics import mean_squared_error
import seaborn as sns
import matplotlib.pyplot as plt
from mlconfound.stats import partial_confound_test
from mlconfound.plot import plot_graph
```

2 Causal Forest on simulated data

[3]:
```
# Generate synthetic data for single simulation
np.random.seed(42)
y, X, treatment, tau, b, e = simulate_nuisance_and_easy_treatment(n=5000)
```

[4]:
```
# Estimate a baseline RandomForest and a causal forest
base_model = DecisionTreeRegressor(random_state=42)
base_model.fit(y=y[:4000], # crime rate
              X=X[:4000] # features
             )

cf_model = CausalForest(random_state=42)
cf_model.fit(y=y[:4000], # crime rate
              T=treatment[:4000], # percent male
```

```
                X=X[:4000] # features
               )

predicted_base = base_model.predict(X[4000:])
predicted_cf = cf_model.predict_full(X[4000:])[:,1]
```

[5]:

```
sns.regplot(x=y[4000:], y=predicted_base, scatter=False)
sns.scatterplot(x=y[4000:], y=predicted_base.flatten(), hue=treatment[4000:])
```

[5]:

```
<AxesSubplot:>
```

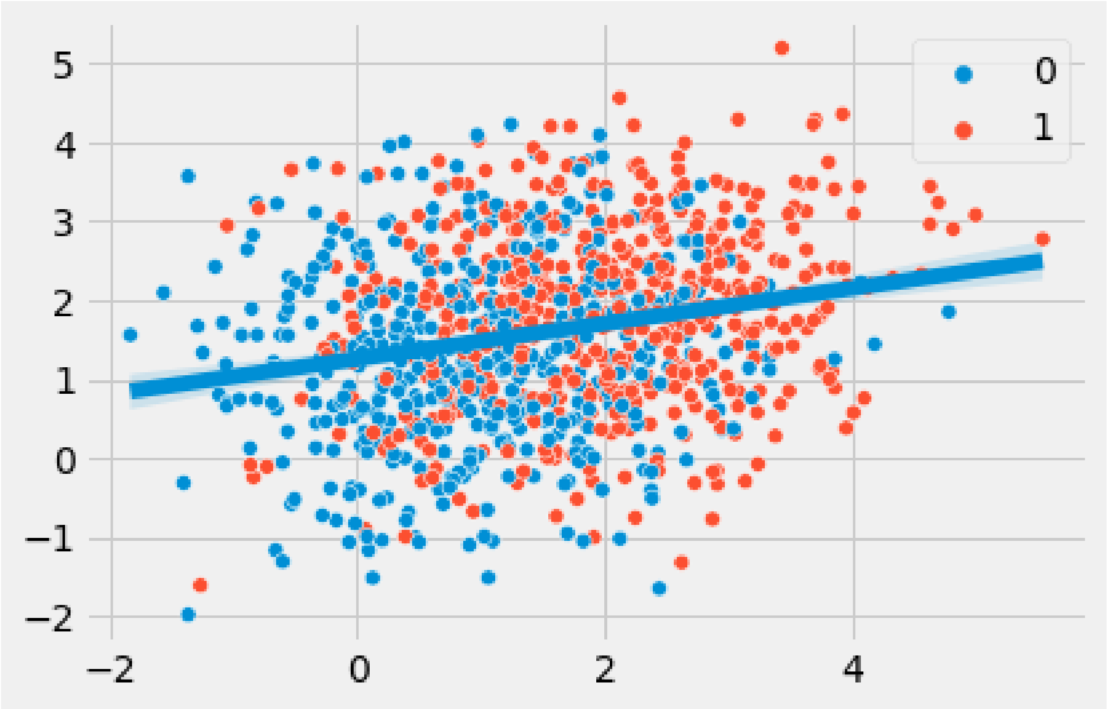

[6]:

```
plot_graph(partial_confound_test(y=y[4000:], yhat=predicted_base.flatten(),␣
↪c=treatment[4000:], cat_c=True))
```

```
Permuting: 100%|

                    | 1000/1000 [00:02<00:00, 364.38it/s]
```

[6]:

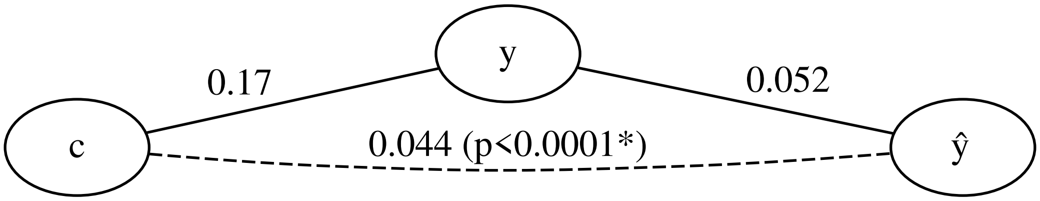

[7]:
```
sns.regplot(x=y[4000:], y=predicted_cf, scatter=False)
sns.scatterplot(x=y[4000:], y=predicted_cf.flatten(), hue=treatment[4000:])
```

[7]: <AxesSubplot:>

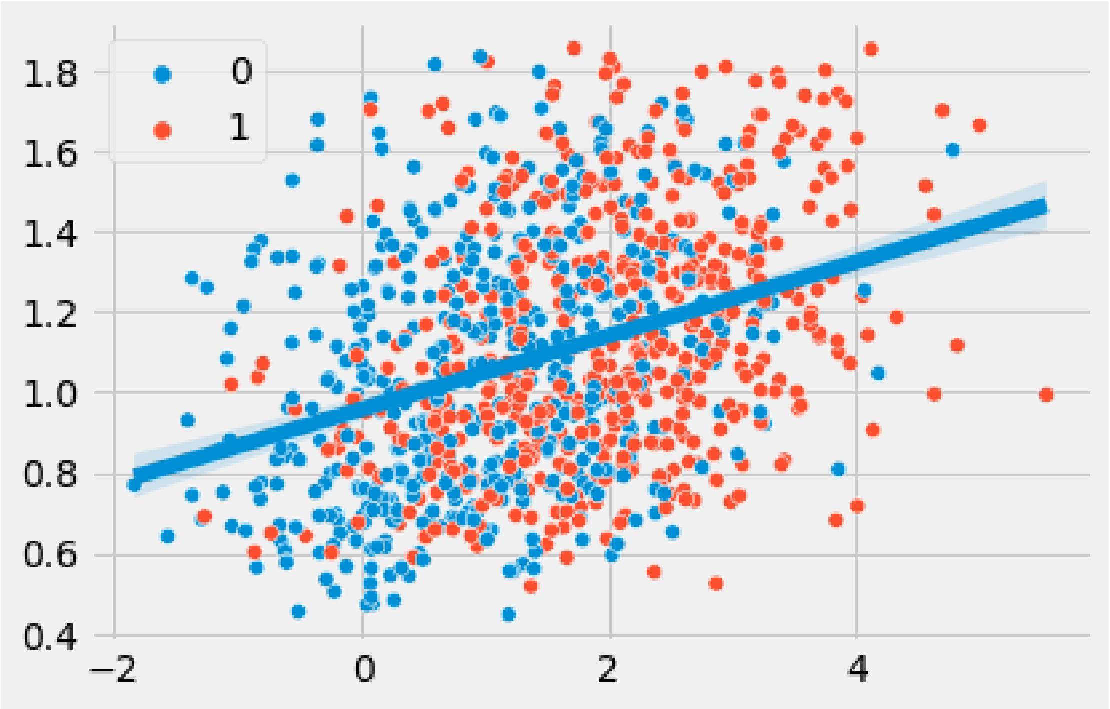

[8]:
```
plot_graph(partial_confound_test(y=y[4000:], yhat=predicted_cf.flatten(),␣
↪c=treatment[4000:], cat_c=True))
```

```
Permuting: 100%|

                | 1000/1000 [00:01<00:00, 504.11it/s]
```

[8]:

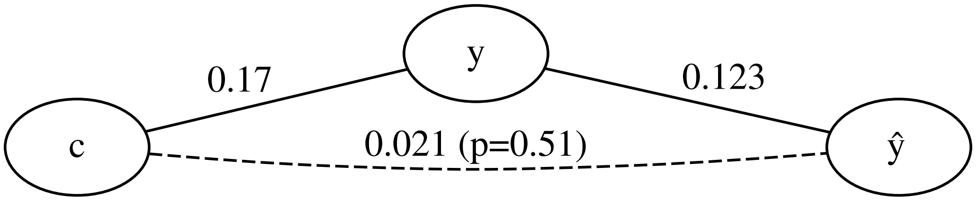

4 ## 3 Now we try it for the HCP dataset

[9]:
```
# HCP data can be obtained from the connectomeDB with special license
# data is not part of this repository
subjectIDs = pd.read_csv('../data/hcp/subjectIDs.txt', header=None)

netmats_pearson = pd.read_csv('../data/hcp/netmats1_correlationZ.txt',
                             sep=' ',
                             header=None)
netmats_pearson['ID'] = subjectIDs[0]
netmats_pearson.set_index('ID', drop=True, inplace=True)


netmats_parcor = pd.read_csv('../data/hcp/netmats2_partial-correlation.txt',
                             sep=' ',
                             header=None)
netmats_parcor['ID'] = subjectIDs[0]
netmats_parcor.set_index('ID', drop=True, inplace=True)

behavior = pd.read_csv('../data/hcp/hcp1200_behavioral_data.csv')
behavior = behavior.set_index('Subject', drop=True)

# convert age to numeric
age = []
for s in behavior['Age']:
    if s == '36+':
        age.append(36)
    else:
        split = s.split(sep='-')
        age.append(np.mean((float(split[0]), float(split[1]))))

behavior['age'] = age
```

[10]:
```
##########################################################
# change these
target = 'PMAT24_A_CR' # fluid intelligence
feature_data = netmats_parcor
########################################################### it's a good␣
 ↪practice to use pandas for merging, messing up subject order can be painful
features = feature_data.columns
df = behavior
df = df.merge(feature_data, left_index=True, right_index=True, how='left')
df = df.dropna(subset = [target] + features.values.tolist())
y = df[target].values
X = df[features].values
rng = np.random.default_rng(42)
```

```
y_trf = quantile_transform(np.array([y+rng.uniform(0,1,len(y))-0.5]).T,␣
↪output_distribution='normal', n_quantiles=1000).flatten()
y=y_trf
```

[11]:
```
model = CausalForest()


# nested cv
outer_cv = KFold(10)
predicted = np.zeros(len(y))

for train, test in outer_cv.split(X, y):

    model.fit(X=X[train], y=y[train], T=df.Acquisition.astype("category").cat.
↪codes.values[train])
    predicted[test] = model.predict_full(X[test])[:,1].flatten()
```

[12]:
```
print("*** Score on mean as model:\t" + str(-mean_squared_error(np.repeat(y.
↪mean(), len(y)), y)))
print("Explained Variance: " +  str( 1- -mean_squared_error(predicted, y)/
↪-mean_squared_error(np.repeat(y.mean(), len(y)), y) ))
print("Correlation: " + str(np.corrcoef(y, predicted)[0,1]))

plt.figure(figsize=(5,2))
sns.regplot(x=y, y=predicted, scatter=False, color='gray')
sns.scatterplot(x=y, y=predicted, hue=df.Acquisition.astype("category").cat.
↪codes.values,
                palette=sns.color_palette("coolwarm", as_cmap=True), alpha=0.4)
```

```
*** Score on mean as model:     -1.0401469064852684
Explained Variance: -0.028955156207362664
Correlation: 0.050185351954835575
```

[12]: <AxesSubplot:>

6

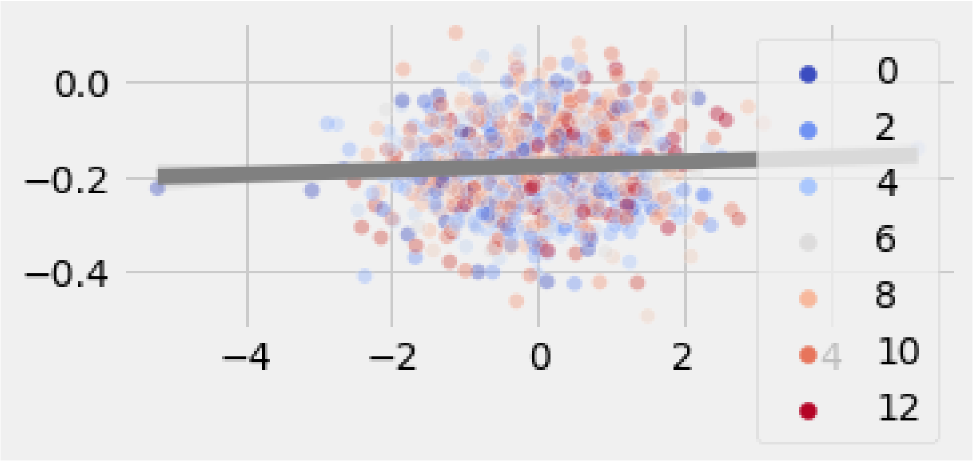

[13]:
```
plot_graph(partial_confound_test(y, predicted, df.Acquisition.
↪astype("category").cat.codes.values,
                                 random_state=42))
```

```
Permuting: 100%|

                    | 1000/1000 [00:01<00:00, 822.76it/s]
```

[13]:

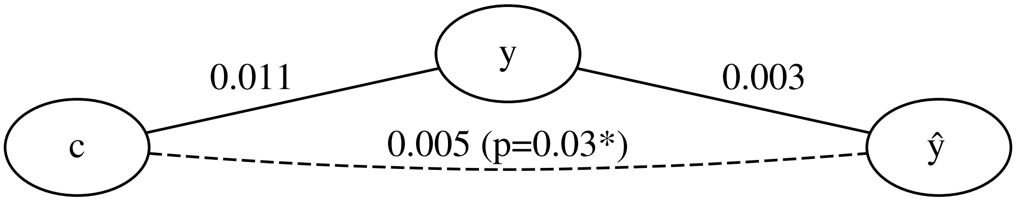

Supplement: giac082_Supplemental_File [file giac082_supplemental_file.pdf]
