# Supplementary material for: Statistical quantification of confounding bias in machine learning models
Source: Gigascience. 2022 Aug 26;11:giac082. doi: 10.1093/gigascience/giac082 (PMC9412867; doi:10.1093/gigascience/giac082)
Supplement: giac082_Response_to_Reviewer_Comments_Original_Submission [file giac082_response_to_reviewer_comments_original_submission.pdf]

Author's Response To Reviewer Comments

Close

Please see the attached pdf file (rebuttal_letter.pdf).

Close
