# Supplementary material for: Statistical quantification of confounding bias in machine learning models
Source: Gigascience. 2022 Aug 26;11:giac082. doi: 10.1093/gigascience/giac082 (PMC9412867; doi:10.1093/gigascience/giac082)
Supplement: giac082_Response_to_Reviewer_Comments_Revision_1 [file giac082_response_to_reviewer_comments_revision_1.pdf]

Author's Response To Reviewer Comments

Close

Dear Dr. Nogoy,

Thank you for considering my manuscript "Statistical quantification of confounding bias in machine learning models" (GIGA-D-22-00097R1) for publication in GigaScience.

This revision includes the following changes:
- I have added the GigaDB DOI to the references and cited it under "availability of supporting data" (l. 730).
- I have included the RRID (Research Resource Identification Initiative ID) and biotoolsID identifiers in the manuscript (l 455).
- I have uploaded the latest latex files into the system

Please don't hesitate to contact me with any further questions/remarks.

Best wishes,
Tamas Spisak

Close
