# Supplementary material for: Statistical quantification of confounding bias in machine learning models
Source: Gigascience. 2022 Aug 26;11:giac082. doi: 10.1093/gigascience/giac082 (PMC9412867; doi:10.1093/gigascience/giac082)
Supplement: giac082_Reviewer_1_Report_Original_Submission — Bertrand Thirion -- 5/30/2022 Reviewed [file giac082_reviewer_1_report_original_submission.pdf]

Reviewer Report

Title: Statistical quantification of confounding bias in machine learning models

Version: Original Submission **Date:** 5/30/2022

Reviewer name: Bertrand Thirion

Reviewer Comments to Author:

The manuscript GIG1-D-22-000197 entitled "Statistical quantification of confounding bias in machine learning models" presents a method to detect with due statistical significance the confoudedness of a given predictor, given an observed confounding factor. The test is based on measuring the partial association of the confound and predicted outcome, given the outcome. This is new, as far as I can tell. Practical algorithms are proposed, based on state-of-the art learning and statistical methods. Validations are performed on simulated data to assess error rate control and good power. Illustrations are provided on two neuroimaging datasets.
Overall, I enjoyed reading the paper, which makes a useful and well-grounded contribution to the developing field of large scale medical studies. I would only link to brain a couple of nuances here and there, but I certainly think that the paper deserves publication in Gigascience.
AFAICT the paper also covers the stet-of-the-art pretty well, which contributed to making it a pleasant reading.
0. A general comment is that there is strong emphasis on the deviation from the linear/Gaussian case. While I can easily understand the concern on linearity, I am less so when it comes to the Gaussian hypothesis. My bet is that in many cases, deviation from normality is mild enough that it should barely make a difference.
To put it differently, the paper could be over-emphasizing high-order statistical aspects, while we should start with second-order statistics, at least for the sake of pedagogy. For instance, on the two datasets considered, does it *really* make a difference whether one relies on (Pearson or Spearman) partial correlation or the proposed test ? If the difference is mild, the concern on non-Gaussianity should be de-emphasized.
1. For the sake of pedagogy, I would like it to be explicitly stated somewhere (introduction or discussion) that the confound has to be observed. This is indeed trivial, but people keep dreaming of somewhat generic or unspecific confoundedness tests.
2. I would remove "Neurobiological" from the abstract as there is no reason to limit the contribution to this field.
3. l.49 "given the target variable." -> I think it is called "output" in the text. I propose to use a fixed terminology throughout the paper.
4. l.103 "whether c **is** independent"
5. The PCT is not very easy to understand at first sight. In particular, when the authors describe a "permutation of the confounder" is it not really clear whether the variable is permuted or whether the residual of a regression model is permuted. At least what I got from Fig. 2 is the the residuals of the regression model are permuted. What I am missing then is why there is a need to rely on MH sampling,

and not simple permutation, under the hypothesis that residuals are exchangeable ?
As a side not, I think that such explanations should aim at pedagogy before technical accuracy, and that details are easier to understand if they are well motivated.
6. Eq (3) has an issue, maybe a missing log on LHS.
7. Why I understand the arguments given in lines 250-260, I am not entirely sure that the use of GAM is really the most natural ore reasonable choice. For instance a random Forest is a relatively elementary, parameter-free approach, that seamlessly handles continuous and discrete variables. I would at least compare it with the proposed GAM in on a simulation setup.
8. Fig.4 only displays power for the proposed method. I would like to see a comparison with an alternative, at least on one column, to assess whether the power of the proposed approach is as good as can be.
9. l.385 it remains unclear for me why y should be Gaussian ? Maybe this is related to the Metropolis sampler ? More deeply, imposing such a constraint may bias the results of the analysis. In some sense, it defeats the purpose of having a test that adapts to all types of noise and relations between variables !
10. l.486 "obtained as shared with the by Dadi et al." you mean that the time-series were directly obtained from Dadi et al ?
11. l. 427-430 that COMBAT cannot handle continuous variables, so that workarounds need to be used is actually quite bad. I feel uneasy when a paper makes a strong point about the respect of statistical properties of the data and then relies on ugly workarounds to work with actual data.
12. l.453 missing references.
13. The caption of Fig.6 should clearly indicate what dataset is used.
14. Fig.7 I don't understand how deconfounding can **increase** the impact of motion, especially to an R^2 of .359 (instead of .111). This hints at a bug or a problem in the experiments. A more detailed explanation should be provided --- or a fix ?
15. l.573-575 "Exactly this can be achieved" -> "This can be achieved exactly"
16. l.598-602: performing sample size estimation requires knowing effect size, which is never known, especially for confounding effects... Maybe I misunderstood the point ?

Methods

Are the methods appropriate to the aims of the study, are they well described, and are necessary controls included? Choose an item.

Conclusions

Are the conclusions adequately supported by the data shown? Choose an item.

Reporting Standards

Does the manuscript adhere to the journal's guidelines on [minimum standards of reporting?](#) Choose an item.

Choose an item.

Statistics

Are you able to assess all statistics in the manuscript, including the appropriateness of statistical tests used? Choose an item.

Quality of Written English

Please indicate the quality of language in the manuscript: Choose an item.

Declaration of Competing Interests

Please complete a declaration of competing interests, considering the following questions:

- Have you in the past five years received reimbursements, fees, funding, or salary from an organisation that may in any way gain or lose financially from the publication of this manuscript, either now or in the future?
- Do you hold any stocks or shares in an organisation that may in any way gain or lose financially from the publication of this manuscript, either now or in the future?
- Do you hold or are you currently applying for any patents relating to the content of the manuscript?
- Have you received reimbursements, fees, funding, or salary from an organization that holds or has applied for patents relating to the content of the manuscript?
- Do you have any other financial competing interests?
- Do you have any non-financial competing interests in relation to this paper?

If you can answer no to all of the above, write 'I declare that I have no competing interests' below. If your reply is yes to any, please give details below.

I declare that I have no competing interests

I agree to the open peer review policy of the journal. I understand that my name will be included on my report to the authors and, if the manuscript is accepted for publication, my named report including any attachments I upload will be posted on the website along with the authors' responses. I agree for my report to be made available under an Open Access Creative Commons CC-BY license (http://creativecommons.org/licenses/by/4.0/). I understand that any comments which I do not wish to be included in my named report can be included as confidential comments to the editors, which will not be published.

Choose an item.

To further support our reviewers, we have joined with Publons, where you can gain additional credit to further highlight your hard work (see: https://publons.com/journal/530/gigascience). On publication of this paper, your review will be automatically added to Publons, you can then choose whether or not to claim your Publons credit. I understand this statement.

Yes Choose an item.
