# Supplementary material for: Statistical quantification of confounding bias in machine learning models
Source: Gigascience. 2022 Aug 26;11:giac082. doi: 10.1093/gigascience/giac082 (PMC9412867; doi:10.1093/gigascience/giac082)
Supplement: giac082_Reviewer_2_Report_Original_Submission — Jean-Baptiste Poline -- 7/2/2022 Reviewed [file giac082_reviewer_2_report_original_submission.pdf]

Reviewer Report

Title: Statistical quantification of confounding bias in machine learning models

Version: Original Submission **Date:** 7/2/2022

Reviewer name: Jean-Baptiste Poline

Reviewer Comments to Author:

Main remarks
The article by T Spisak develops a statistical test to rule out the implication of a confounder in the model prediction. The work is based mostly on Berrett TB et al, JRSS, 2020, and expand on it to test the partial confounding effect.
The article presents both simulations and results on the HCP and ABIDE datasets. This is a solid article that deserves publication.
One key aspect however is that the status of confounder in predictive model should be better described. This relate to the goal of the predictive model and its interpretation. In a number of cases, removing or controling for the confounder can harm the prediction and not all prediction task require interpretation (see for instance Fig 1. of Dockes et al, 2021, Gigascience).
I recommend that this point is further clarified in the introduction.
The release of the python package mlconfound is very commendable and deserves praise.
Details
Could the author clarify if the test is on
\hat{y} \ind c|y
or on
\hat{y}|y \ind c|y
Notations: confounder is denoted c - but then the notation seems to move to Z
Grid search are very greedy to chose the hyperparameters when regularizing the GAM - this could offset some computational advantages described in the article.
Since predictive models are designed to be used on out of sample, it would be interesting to illustrate how the designed test relates to confounding effect on out of sample and out of study predictive model results.

Methods

Are the methods appropriate to the aims of the study, are they well described, and are necessary controls included? Choose an item.

Conclusions

Are the conclusions adequately supported by the data shown? Choose an item.

Reporting Standards

Does the manuscript adhere to the journal's guidelines on [minimum standards of reporting?]() Choose an item.

Choose an item.

Statistics

Are you able to assess all statistics in the manuscript, including the appropriateness of statistical tests used? Choose an item.

Quality of Written English

Please indicate the quality of language in the manuscript: Choose an item.

Declaration of Competing Interests

Please complete a declaration of competing interests, considering the following questions:

- Have you in the past five years received reimbursements, fees, funding, or salary from an organisation that may in any way gain or lose financially from the publication of this manuscript, either now or in the future?
- Do you hold any stocks or shares in an organisation that may in any way gain or lose financially from the publication of this manuscript, either now or in the future?
- Do you hold or are you currently applying for any patents relating to the content of the manuscript?
- Have you received reimbursements, fees, funding, or salary from an organization that holds or has applied for patents relating to the content of the manuscript?
- Do you have any other financial competing interests?
- Do you have any non-financial competing interests in relation to this paper?

If you can answer no to all of the above, write 'I declare that I have no competing interests' below. If your reply is yes to any, please give details below.

I declare that I have no competing interests

I agree to the open peer review policy of the journal. I understand that my name will be included on my report to the authors and, if the manuscript is accepted for publication, my named report including any attachments I upload will be posted on the website along with the authors' responses. I agree for my report to be made available under an Open Access Creative Commons CC-BY license (http://creativecommons.org/licenses/by/4.0/). I understand that any comments which I do not wish to be included in my named report can be included as confidential comments to the editors, which will not be published.

Choose an item.

To further support our reviewers, we have joined with Publons, where you can gain additional credit to further highlight your hard work (see: https://publons.com/journal/530/gigascience). On publication of this paper, your review will be automatically added to Publons, you can then choose whether or not to claim your Publons credit. I understand this statement.

Yes Choose an item.
